# Cognitively defined Alzheimer's dementia subgroups have distinct atrophy patterns

**DOI:** 10.1002/alz.13567

**Published:** 2023-12-13

**Authors:** Paul K. Crane, Colin Groot, Rik Ossenkoppele, Shubhabrata Mukherjee, Seo‐Eun Choi, Michael Lee, Phoebe Scollard, Laura E. Gibbons, R. Elizabeth Sanders, Emily Trittschuh, Andrew J. Saykin, Jesse Mez, Connie Nakano, Christine Mac Donald, Harkirat Sohi, for the Alzheimer's Disease Neuroimaging Initiative, Shannon Risacher

**Affiliations:** ^1^ Department of Medicine University of Washington Seattle Washington USA; ^2^ Clinical Memory Research Unit Lund University Lund Sweden; ^3^ Alzheimer center Amsterdam UMC ‐ VU Medical Center Amsterdam Netherlands; ^4^ Department of Psychiatry and Behavioral Sciences University of Washington, and Geriatrics Research Education, and Clinical Center VA Puget Sound Health Care System Seattle USA; ^5^ Indiana Alzheimer's Disease Research Center Indiana University School of Medicine Indianapolis USA; ^6^ Department of Radiology and Imaging Sciences Indiana University School of Medicine Indianapolis USA; ^7^ Department of Neurology Boston University Boston Massachusetts USA; ^8^ Department of Neurosurgery University of Washington Seattle USA; ^9^ Department of Biomedical Informatics and Medical Education University of Washington Seattle USA; ^10^ Now Pacific Northwest National Laboratory Richland USA

**Keywords:** Alzheimer's disease dementia, asymmetry, atrophy, cognitive heterogeneity, neurodegeneration, subgroups

## Abstract

**INTRODUCTION:**

We sought to determine structural magnetic resonance imaging (MRI) characteristics across subgroups defined based on relative cognitive domain impairments using data from the Alzheimer's Disease Neuroimaging Initiative (ADNI) and to compare cognitively defined to imaging‐defined subgroups.

**METHODS:**

We used data from 584 people with Alzheimer's disease (AD) (461 amyloid positive, 123 unknown amyloid status) and 118 amyloid‐negative controls. We used voxel‐based morphometry to compare gray matter volume (GMV) for each group compared to controls and to AD‐Memory.

**RESULTS:**

There was pronounced bilateral lower medial temporal lobe atrophy with relative cortical sparing for AD‐Memory, lower left hemisphere GMV for AD‐Language, anterior lower GMV for AD‐Executive, and posterior lower GMV for AD‐Visuospatial. Formal asymmetry comparisons showed substantially more asymmetry in the AD‐Language group than any other group (*p* = 1.15 × 10^−10^). For overlap between imaging‐defined and cognitively defined subgroups, AD‐Memory matched up with an imaging‐defined limbic predominant group.

**DISCUSSION:**

MRI findings differ across cognitively defined AD subgroups.

## 1 BACKGROUND

A personalized medicine approach has been recommended for Alzheimer's disease (AD).^1^, ^2^ One success story for personalized medicine is breast cancer. Separating people with “breast cancer” into groups based on receptors is commonplace today; subtype‐specific treatments have reduced mortality.^3^

A similar strategy may be applicable to AD dementia. Heterogeneity among people with AD dementia may impede research progress into prevention and treatment.^4^ Identifying more homogeneous subgroups of people with AD dementia may be important in advancing research and may ultimately have therapeutic implications.

We previously developed an approach to subtyping people with typical late‐onset AD dementia based on relative impairments across cognitive domains.^5^ Neuropsychologists use relative impairments to help determine dementia type.^6^ We extend this paradigm to further characterize relative impairments across domains within the AD dementia spectrum.

We have found differences between cognitively defined subgroups in terms of genetic markers (multiple cohorts),^7^ associations with depression (three cohorts),^8^ trajectories of glucose metabolism in Alzheimer's Disease Neuroimaging Initiative (ADNI) participants with FDG‐PET scans,^9^ and regional gray matter volumes (GMVs) from a tertiary memory clinic cohort.^10^

In this paper, we use ADNI's structural magnetic resonance imaging (MRI) data to compare GMV patterns across cognitively defined subgroups. We identified amyloid‐positive or ‐unknown individuals who enrolled in ADNI with AD dementia or who developed AD dementia while enrolled. We compared GMVs across subgroups and with those from stable amyloid‐negative cognitively normal (CN) older adults. We used an atlas‐based approach to formally evaluate asymmetry. We stratified each group into quartiles based on overall atrophy to evaluate pseudo‐progression within subgroups. Finally, we compared our cognitively defined subgroups to anatomically defined subgroups^11^, ^12^ and to subgroups identified based on data‐driven differences in structural MRI findings.^12^

## 2 METHODS

### 2.1 Participants

Data are from ADNI. ADNI detailed methods are published.^13^, ^14^ ADNI was launched in 2003 as a public–private partnership, led by Principal Investigator Michael W. Weiner, MD. ADNI's primary goal has been to test whether serial MRI, positron emission tomography (PET), other biological markers, and clinical and neuropsychological assessment can be combined to measure the progression of mild cognitive impairment (MCI) and early AD dementia. For up‐to‐date information, see www.adni‐info.org.

We included individuals who were enrolled with AD dementia (prevalent) or who progressed to AD dementia (incident) in any phase of ADNI up to ADNI3. A flow chart detailing exclusions and the final sample is in Figure S1. We included those with onset age ≥ 65 years. We considered data from the first dementia visit: the first study visit (prevalent cases) and the visit AD dementia was diagnosed (incident cases). We used MRI data closest to the first dementia visit and excluded participants without a scan that passed quality control (see below) within 1.5 years of that visit (20 participants excluded). All included AD dementia participants had a stable diagnosis following the first dementia visit (13 people excluded for MCI reversion). CN controls were those who had normal cognition at enrollment and who continued without converting to MCI or to dementia during ADNI.

We used established cut‐offs to determine amyloid positivity. We included people with AD dementia who were known to be amyloid positive (see below) or for whom amyloid status was unknown (*n* = 123); we excluded 56 people defined by ADNI as having AD dementia who were amyloid negative.

Amyloid levels change slowly,^15^ so we allowed amyloid data from PET or cerebrospinal fluid (CSF) biomarkers up to 3 years from the first dementia visit. There were 200 people with AD dementia who were amyloid positive based on CSF assessment only (Elecsys Aβ42 < 1098 pg/mL^16^); 17 based on [^11^C]PiB (global cortical standardized uptake value ratio [SUVR] ≥ 1.5^17^), 66 based on [^18^F]florbetapir (global cortical SUVR ≥ 1.10^18^), 12 based on [^18^_F_]florbetaben (global cortical SUVR ≥ 1.11^19^), and 166 using both CSF and PET; in all there were 461 amyloid‐positive cases. There were 17 people with AD dementia who had discrepant amyloid status between CSF and PET; all were included as amyloid‐positive individuals here. Of these, 14 were amyloid positive based on CSF but not PET, and three were amyoid positive based on PET but not CSF.

Amyloid negative CN controls used the same cut‐offs. There were 30 based on CSF, 27 based on [^18^F]florbetapir, and 61 with both CSF and PET, totaling 118 controls.

### 2.2 Diversity, equity, and inclusion (DEI)

ADNI had no exclusion criteria for any group defined on the basis of ethnicity or race. ADNI enrollment was characterized by overrepresentation of people with European ancestry.  Current ADNI funding focuses specifically on enhancing diversity in new enrollees.  The present analyses are of data from the earlier parts of the ADNI study.

### 2.3 Cognitive measures and subgrouping

We obtained cognitive domain scores^7^ and determined cognitively defined subgroups^5^ as described.^5^, ^7^, ^8^, ^9^, ^10^ As previously and as discussed in those prior publications, we did not include the attention domain. ADNI assessed cognition with a full neuropsychological battery using measures of memory, executive function, language, and visuospatial abilities. An expert panel (ET, JM, AS, PC) considered each item administered and assigned it to one domain (memory, executive function, language, and visuospatial function) or “other.” We used bifactor confirmatory factor analysis approaches using Mplus^20^ to generate composite scores for each domain. We co‐calibrated ADNI data with those from other studies. We used scores from 825 people with incident AD dementia from Adult Changes in Thought (ACT) to define the mean at 0 and standard deviation (SD) at 1, as described.^7^ We excluded eight cases due to insufficient cognitive data for all four domain scores (Figure S1).

**RESEARCH IN CONTEXT**

1. **Systematic review**: The authors reviewed the literature using traditional (eg, PubMed) sources for AD subgroups and symmetry. Relevant papers are cited.
2. **Interpretation**: We found structural imaging differences across cognitively defined subgroups, with disparate atrophy patterns at AD dementia diagnosis. This paper presents side‐by‐side comparisons with very similar findings from a prior publication. Our results showed concordance between the AD‐Memory subgroup and the limbic‐predominant subgroup defined based on imaging. The AD‐Language subgroup had much greater left‐ than right‐sided atrophy. Usually, AD is thought to have symmetrical imaging and neuropathology findings.
3. **Future directions**: This manuscript provides additional data supporting the notion that typical late‐onset AD dementia may represent multiple biologically distinct subgroups. Such a conclusion would have important implications for a personalized medicine approach to AD dementia, as risk factors, biological mechanisms, responses to therapy, and natural history may all vary across different subgroups.

Our approach to subgrouping is schematically illustrated in Figure S2. For each case, we determined the average of memory, executive functioning, language, and visuospatial scores. We then determined the difference between each domain score and that average. As published, we used a difference of 0.80 units to identify domains with scores substantially lower than the individual average. We considered the number of domains substantially lower than the individual average. Those with no such domains (ie, all scores similar) were AD‐No Domain. Those with a single such domain were categorized as AD‐Memory, AD‐Language, AD‐Visuospatial, or AD‐Executive. Those with multiple such domains were categorized as AD‐Multiple Domains.

### 2.4 MRI processing

Structural MRI data were downloaded from www.adni.loni.usc.edu. Scans were corrected prior to download as described^21^ ^22^ for ADNI‐1 and ADNIGO/2 scans. For ADNI‐3 ADNI is no longer generating corrected scans due to improved scan quality. Scans were processed using voxel‐based morphometry (VBM) in SPM12 with DARTEL. Briefly, using a standard DARTEL‐based SPM12 processing pipeline, scans were segmented into CSF, white matter volumes, and GMVs. Segmented scans were rigidly aligned to a T1 template to ensure overlap in Montreal Neurological Institute (MNI) space. These aligned scans were then co‐registered using non‐linear and high‐dimensional warping, smoothed with an 8‐mm full‐width‐at‐half‐maximum isotropic Gaussian kernel, modulated to preserve tissue volume signal, and spatially normalized to MNI space.^10^ Quality control via visual inspection was done after every pre‐processing stage; 36 people with AD dementia were excluded based on these checks.

### 2.5 W‐scores

Regional and global atrophy is operationalized by W‐scores, which represent covariate‐adjusted Z‐scores normalized against CN controls.^23^ For each voxel we determined gray matter (GM) density distribution controlling for age, sex, field strength, and intracranial volume. We used these findings to determine W‐scores for each voxel for each included participant. We used mean W‐scores to account for overall progression in subsequent models.

### 2.6 Statistical analyses

#### 2.6.1 Voxel‐based morphometry

We compared demographic, neuropsychological, and clinical variables with linear regression and chi‐squared tests. We compared normalized GM volume images on a voxel‐by‐voxel basis across groups using a one‐way analysis of covariance covaried for age at scan, sex, years of education, total intracranial volume, field strength (1.5T vs 3T), and global mean W‐score.^23^ Statistical maps were generated at a *p* < 0.05 threshold with cluster‐wise multiple comparison correction (voxel‐wise threshold *p* < 0.001, minimum cluster size 840 voxels). We displayed beta maps using MRIcronGL (https://www.nitrc.org/plugins/mwiki/index.php/mricrogl:MainPage). Spatial maps representing CN control/AD subgroup differences were displayed at the same minimum and maximum threshold (β = 0 to 0.063); all AD subgroup differences used β = 0 to 0.035.

### 2.7 Asymmetry analyses

We used FreeSurfer version 5.1 to create regions of interest (ROIs) based on the Desikan‐Killiany atlas.^24^ We extracted ROI GMVs for each subgroup and for 30 randomly selected CN controls. We determined adjusted mean ROI GMV with linear regression, controlling for age, sex, handedness, total intracranial volume, field strength, and global mean W‐score. We used adjusted mean ROI GMVs to calculate asymmetry metrics for each region: (left GMV − right GMV)/(left GMV + right GMV). This formula is negative when the left‐side volume is smaller than the right, and positive when the left is larger. For each region, we fit regression models with robust standard errors using a subgroup indicator, with CN controls as reference. Tabulated values are standardized coefficients; bold indicates *p* < 0.05. All the findings reported represent differences compared to the amount of asymmetry found in the CN controls. Any differences in the number of voxels in a region between the right and left sides would be reflected by the finding for the CN controls and would not explain differences across subgroups with respect to that reference category. We compared the number of statistically significantly regional differences by subgroup using Fisher's exact test.

### 2.8 Pseudo‐progression

Within each group we determined quartiles of overall atrophy based on W‐score voxel count, the number of voxels with W ← 1.5. We plotted mean GMV maps for each quartile of each subgroup using MRIcroGL.

### 2.9 Comparison of cognitively defined subgroups to other systems of subgrouping

Murray et al. proposed to differentiate people with AD based on neuropathology data.^25^ They quantified tau tangles in hippocampus and neocortex and defined a “limbic‐predominant” subtype with high hippocampal and low neocortical tau loads, a “hippocampal sparing” subtype with low hippocampal and high neocortical tau loads, and a “typical” group with similar hippocampal and neocortical tau loads.^25^ Several investigators have applied this framework to imaging data.^11^, ^25^, ^26^, ^27^, ^28^, ^29^, ^30^, ^31^, ^32^, ^33^, ^34^, ^35^, ^36^, ^37^

We used the Risacher et al. approach to categorize people into anatomically defined subgroups.^11^ We considered the same scanning occasion discussed previously. We used multinomial logistic regression models with AD‐No Domain as reference. We excluded AD‐Executive (the smallest cognitively defined subgroup); no individual with AD‐Executive was categorized as limbic predominant.

We were curious as to the stability of anatomically defined subtypes over time in ADNI. We evaluated first and most recent ADNI scans and considered subgroup stability from enrollment to the first dementia visit and from first dementia visit to the most recent study visit.

Finally, we obtained group assignments from Poulakis et al.^12^ They used a Bayesian clustering approach with longitudinal structural imaging data. We used multinomial logistic regression to compare cognitively defined subgroups to the approach used by Poulakis et al.

### 2.10 Standard protocol approvals and patient consents

All data are from ADNI. All ADNI participants signed informed consent forms. University of Washington Institutional Review Board approval is STUDY00008205.

## 3 RESULTS

### 3.1 Demographic and clinical characteristics

There were 584 people with AD dementia and 118 CN controls included. Table 1 summarizes demographic and clinical characteristics. People with AD dementia were older than CN controls, though there was substantial overlap in age. Educational attainment was lower for people with AD dementia (*p* = 0.016); educational differences across subgroups were not statistically significant (*p* = 0.29).

**TABLE 1:** Demographic and clinical characteristics.

Characteristic	Cognitively normal controls	All people with AD	*P* value	AD‐No Domain	AD‐Memory	AD‐Language	AD‐Visuospatial	AD‐Executive	AD‐Multiple Domains	*P* value
*N*	118	584		244	192	42	66	20	20	
Age, mean (SD)	73.5 (6.0)	76.9 (5.9)	2.5 × 10^−8^	76.9 (5.7)	77.0 (6.1)	78.3 (6.3)	76.8 (5.6)	73.6 (7.2)	75.8 (4.5)	0.09
Female sex, *n* (%)	59 (50%)	264 (45%)	0.34	117 (48%)	91 (47%)	16 (38%)	25 (38%)	7 (35%)	8 (40%)	0.49
Education										
Up to high school graduation	14 (12%)	124 (21%)	0.016	62 (25%)	36 (19%)	9 (21%)	13 (20%)	2 (10%)	2 (10%)	0.29
College to college graduation	51 (43%)	266 (46%)		96 (39%)	99 (52%)	22 (50%)	32 (48%)	9 (45%)	9 (45%)	
After college	53 (45%)	194 (33%)		86 (35%)	57 (30%)	12 (29%)	21 (32%)	9 (45%)	9 (45%)	
Incident vs prevalent AD										
Incident (MCI 1st visit)	0	290 (50%)	n/a	120 (49%)	103 (54%)	20 (48%)	32 (48%)	5 (25%)	10 (50%)	0.29
Prevalent (AD 1st visit)	0	294 (50%)		124 (51%)	89 (46%)	22 (52%)	34 (52%)	15 (75%)	10 (50%)	
Race/Ethnicity										
Non‐Hispanic White	106 (90%)	526 (90%)	0.94	221 (91%)	174 (91%)	34 (81%)	62 (94%)	18 (90%)	17 (85%)	0.31^a^
Hispanic or non‐White	12 (10%)	58 (10%)		23 (9%)	18 (9%)	8 (19%)	4 (6%)	2 (10%)	3 (15%)	
Left‐handed	13 (11%)	40 (7%)	0.12	12 (5%)	16 (8%)	5 (12%)	2 (3%)	4 (20%)	1 (5%)	0.056^a^
*APOE* genotype										
0 ε4 alleles	98 (84%)	167 (30%)	9.5 × 10^−28^	68 (29%)	53 (28%)	15 (37%)	20 (32%)	4 (22%)	7 (35%)	0.85
≥ 1 ε4 alleles	19 (16%)	396 (70%)		166 (71%)	134 (72%)	26 (63%)	43 (68%)	14 (78%)	13 (65%)	
MMSE, mean (SD)	29.1 (1.2)	23.3 (2.9)	8.6 × 10^−78^	23.7 (3.0)	23.1 (2.7)	22.8 (3.2)	23.6 (2.9)	22.6 (2.6)	22.9 (2.3)	0.12
CDR sum of boxes, mean (SD)	0.01 (0.1)	4.3 (1.7)	1.6 × 10^−108^	4.2 (1.8)	4.3 (1.7)	4.7 (1.6)	4.3 (1.7)	4.5 (2.0)	4.7 (1.8)	0.95
Cognitive domain scores										
ADNI‐Mem score	1.1 (0.5)	−0.8 (0.5)	8.1 × 10^−148^	−0.7 (0.5)	−1.0 (0.5)	−0.9 (0.6)	−0.6 (0.5)	−0.6 (0.7)	−0.8 (0.5)	4.9 × 10^−8^
ADNI‐EF score	1.0 (0.8)	−0.8 (0.9)	1.7 × 10^−63^	−0.9 (0.9)	−0.4 (0.8)	−1.0 (0.9)	−0.8 (1.1)	−1.8 (0.7)	−0.9 (0.9)	1.6 × 10^−13^
ADNI‐Lan score	0.9 (0.7)	−0.7 (0.9)	5.7 × 10^−63^	−0.8 (0.8)	−0.4 (09.8)	−1.9 (0.9)	−0.4 (0.8)	−0.7 (0.7)	−0.8 (0.8)	1.1 × 10^−24^
ADNI‐VS score	0.3 (0.6)	−0.5 (09)	6.6 × 10^−16^	−0.5 (0.8)	0.0 (0.7)	−0.3 (0.8)	−1.6 (0.9)	−0.4 (0.8)	−0.5 (0.8)	1.2 × 10^−35^
Missing amyloid status	0	127 (22%)	–	54 (22%)	47 (24%)	9 (21%)	9 (14%)	4 (20%)	4 (20%)	0.63

^a^ *P* values are from *t* tests or *F* tests except for those marked with an asterisk, which are from Fisher's exact tests.

Almost half of those with AD dementia had prevalent dementia at ADNI enrollment, and the other half developed incident dementia. Proportions of prevalent versus incident dementia were similar across subgroups (*p* = 0.29). The apolipoprotein E (*APOE*) ε4 allele was more common among people with AD dementia than CN but did not vary significantly across subgroups (*p* = 0.85).

Table 1 also summarizes cognitive data. People with AD dementia had lower Mini‐Mental State Examination (MMSE), Clinical Dementia Rating (CDR) sum of boxes, ADNI‐Memory, ADNI‐Executive, ADNI‐Language, and ADNI‐Visuospatial scores than CN controls (all *p* < 0.0001). MMSE (*p* = 0.12) and CDR sum of boxes (*p* = 0.95) did not differ across subgroups. As expected, mean domain scores were closely matched for people in the AD‐No Domain and AD‐Multiple Domains groups, while individual domain scores were substantially lower for the index domain (the single domain with relative impairment) for the other groups (all *p* < 0.0001).

### 3.2 Subgroup voxel‐based morphometry analyses compared with cognitively normal controls

**FIGURE 1:**
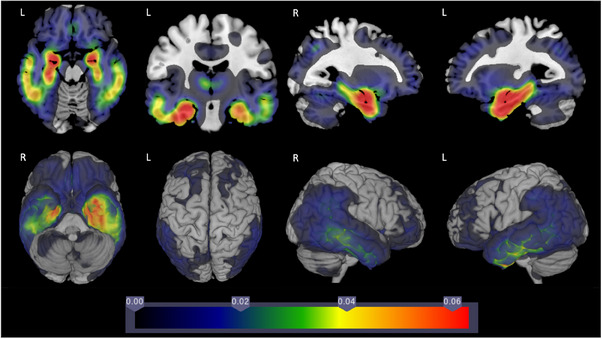
Comparison of gray matter volume β coefficients for AD‐No Domains group and cognitively normal elderly controls.

Several subgroups’ VBM findings compared with CN controls appeared similar. Figure 1 shows β coefficient findings for AD‐No Domains. Colors indicate voxels where the AD‐No Domain group had lower GMV than CN controls. Medial temporal lobe and symmetrical bilateral temporal cortex involvement is apparent. Figure S3 shows *p* value results.

Findings were similar for AD‐Memory, AD‐Visuospatial, AD‐Executive, and AD‐Multiple Domains subgroups; β coefficient and *p* value findings are in Figures S4 to S11.

**FIGURE 2:**
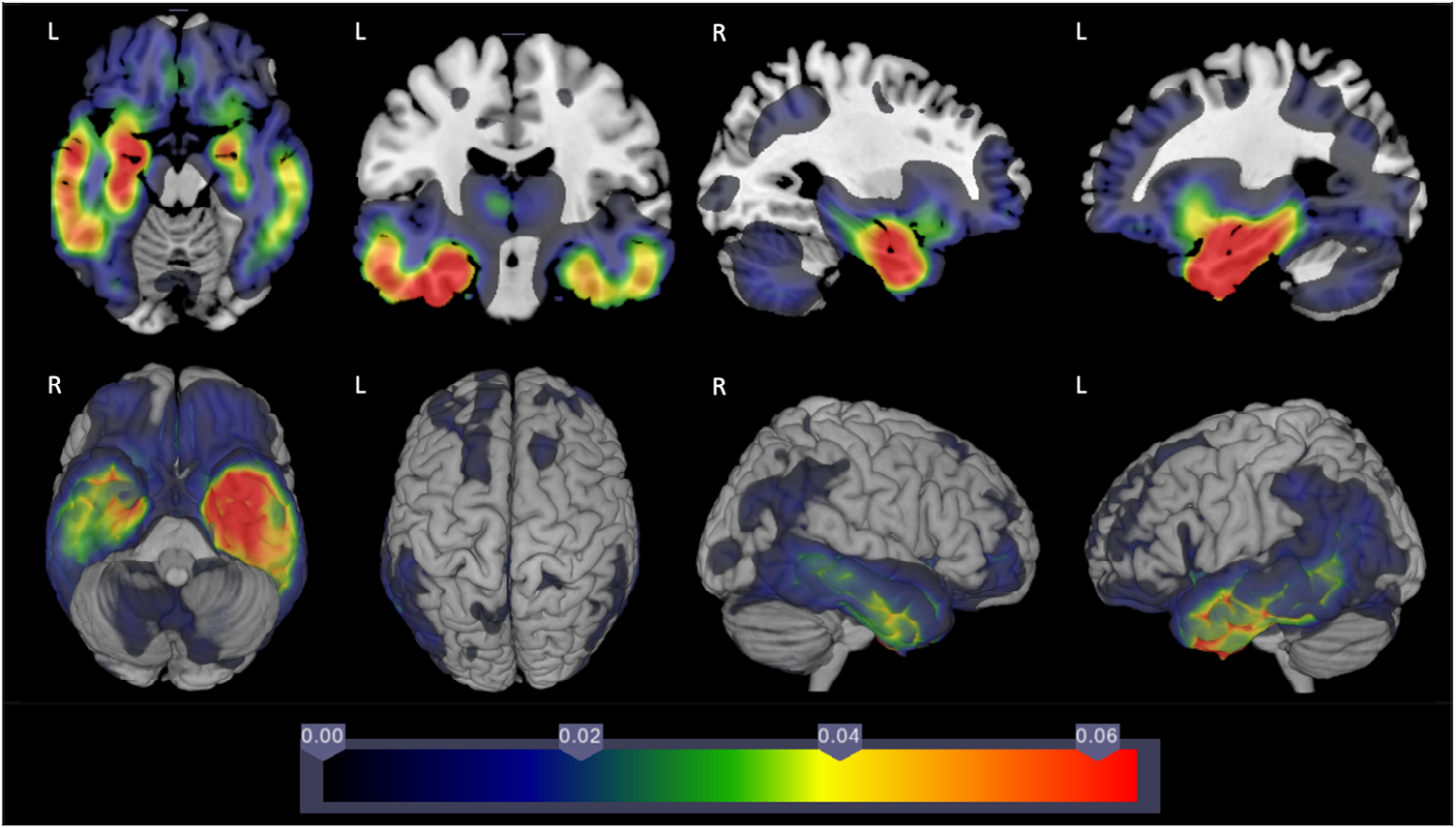
Comparison of gray matter volume for AD‐Language group and cognitively normal elderly controls.

The AD‐Language subgroup differed from this pattern (Figure 2). We noted asymmetrical GMV differences. *P* values are in Figure S12. β coefficient comparisons for all groups are shown in Figure S13, and *p* values for all groups are shown in Figure S14.

### 3.3 Voxel‐based morphometry analyses of subgroups compared with AD‐Memory subgroup

**FIGURE 3:**
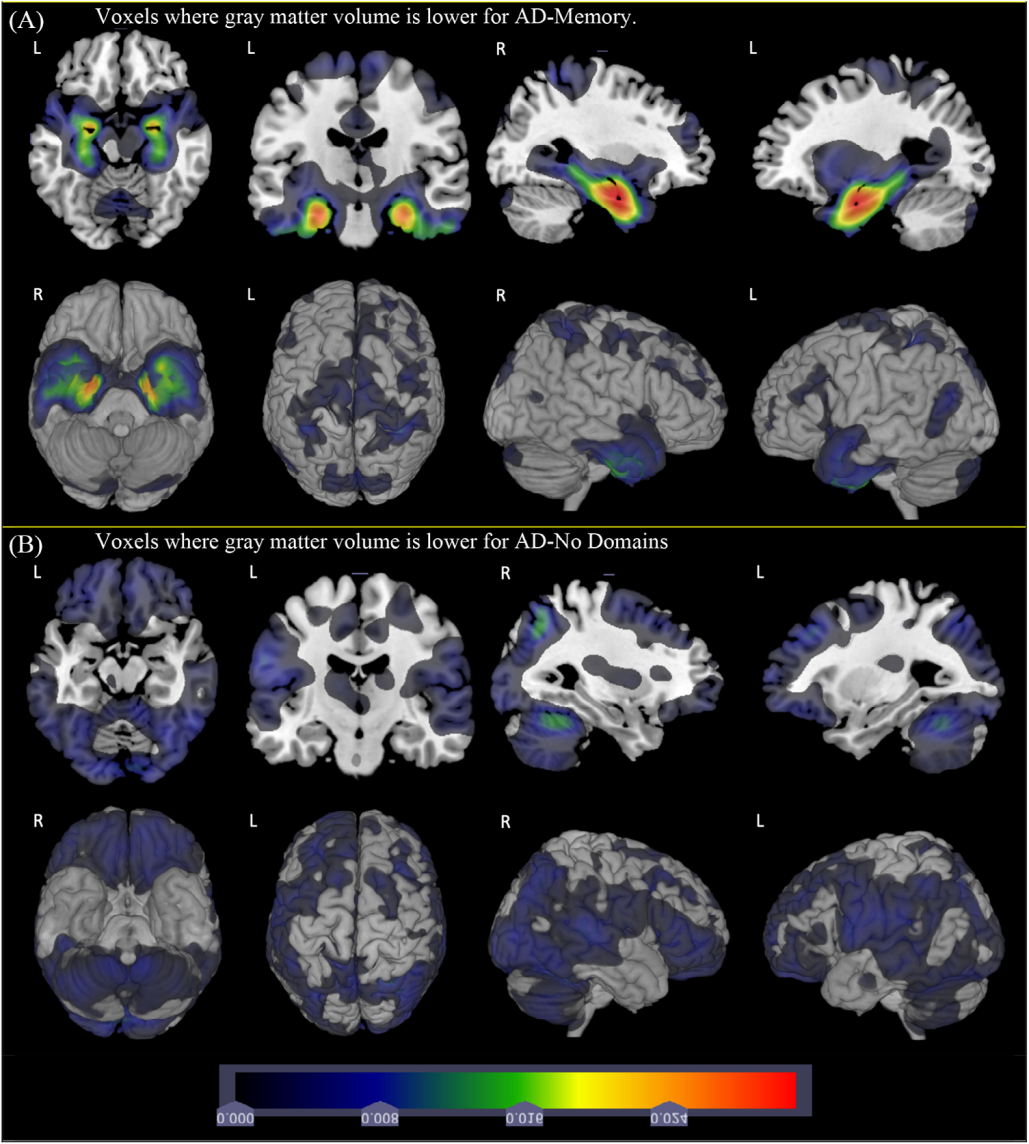
Comparison of gray matter volume for AD‐Memory group with AD‐No Domain group.

Contrasts between subgroups were apparent. Figure 3 shows AD‐No Domain versus AD‐Memory groups. Figure 3A shows voxels where the AD‐Memory group has lower GMV than AD‐No Domain; Figure 3B shows voxels where the AD‐No Domain group has lower GMV than the AD‐Memory group.

Medial temporal involvement compared to controls was evident for both these groups (Figures 1 and S4), and Figure 3A shows even greater involvement for the AD‐Memory group. Figure 3B shows broad cortical involvement outside medial temporal lobes where AD‐No Domain has lower GMV than AD‐Memory. Figure S15 shows corresponding *p* value comparisons.

**FIGURE 4:**
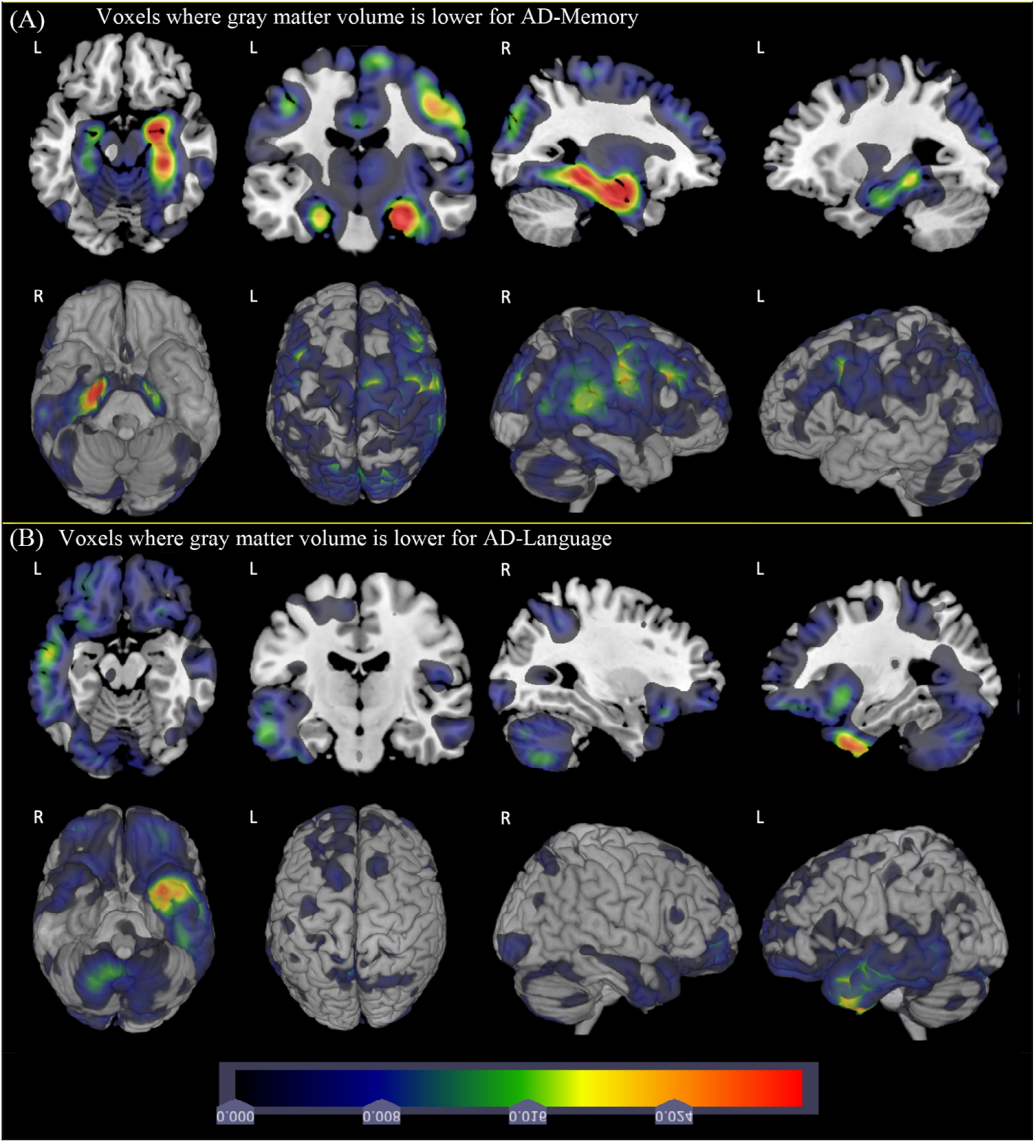
Comparison of gray matter volume for AD‐Memory group with AD‐Language group. *Marginal totals for each subgroup are shown at bottom; for example, 17% of the people with Alzheimer's disease (AD) dementia who met the inclusion criteria were defined as having limbic‐predominant AD. We used the scan at the first AD dementia visit to determine subtypes. These data are also tabulated in Table S2.

Figure 4 shows comparisons between AD‐Memory and AD‐Language. We observed greater hippocampal involvement for AD‐Memory bilaterally, but more pronounced on the right. Large portions of the right hemisphere have lower GMV in AD‐Memory, while there are left temporal cortical areas with lower GMV in AD‐Language. Figure S16 shows enlargements of left temporal cortex, medial temporal lobe, and hippocampus. This figure shows more atrophy in the lateral temporal cortex in AD‐Language and more atrophy in the medial temporal lobe and hippocampus in AD‐Memory.

Figure S17 shows comparisons of AD‐Memory and AD‐Visuospatial. There was lower GMV in the bilateral hippocampi and medial temporal lobes in AD‐Memory and lower GMV in bilateral cortical regions for AD‐Visuospatial. Figure S18 shows the corresponding *p* values.

Figure S19 shows comparisons of AD‐Memory and AD‐Executive. There was lower GMV in bilateral hippocampi in AD‐Memory and lower cortical GMV anteriorly in AD‐Executive. Figure S20 shows comparisons of AD‐Memory and AD‐Multiple Domains. While not as stark as contrasts with other subgroups, there was greater hippocampal involvement in AD‐Memory. There were scattered areas of cortex with greater involvement in AD‐Multiple Domains.

### 3.4 Comparison with previously published findings

All these findings closely replicate those previously reported from a distinct study population.^10^ Figures S21 to S25 show prior and current findings side by side. VBM uses proximity‐based smoothing procedures but is agnostic in terms of anatomical relationships beyond distance. Brain regions that differ most from controls show clear replication.

### 3.5 Regional asymmetry comparisons

Distinct asymmetry for the AD‐Language group compared with CN controls (Figure 2) and compared with AD‐Memory (Figure 4) led us to consider formal asymmetry tests. Selected results from these analyses for right‐handed individuals are shown in Table 2. The full results of all of the asymmetry analyses performed are in Tables S1 and S2. AD‐Language had four regions with statistically significant differences when limited to right‐handed people, and there were no such regions in any of the other groups (Fisher's exact test *p* = 0.004).

**TABLE 2:** Asymmetry results for right‐handed people in Z‐score units. Negative numbers occur with lower volume on left compared with right.

	AD‐No Domain	AD‐Memory	AD‐Language	AD‐Visuospatial	AD‐Executive	AD‐Multiple Domains
Global cortex	−0.23	−0.23	−**2.51**	−0.27	−0.69	0.08
Frontal						
Frontal lobe	−0.21	−0.18	−1.13	−0.28	0.25	−0.17
Regional measures						
Medial orbitofrontal	0.00	−0.46	−0.46	−0.29	0.40	−0.64
Middle frontal	−0.26	−0.12	−0.71	−0.29	0.08	0.14
Pars opercularis	−0.08	−0.33	−0.58	−0.27	0.18	−0.22
Pars orbitalis	−0.07	−0.13	−0.22	0.06	0.10	−0.61
Pars triangularis	−0.16	−0.32	−0.52	−0.24	−0.10	−0.45
Precentral	−0.12	0.00	−0.68	−0.10	−0.12	−0.11
Rostral middle frontal	−0.15	−0.09	−0.52	−0.25	0.23	−0.03
Parietal						
Parietal lobe	−0.10	−0.09	−1.19	−0.06	−0.41	0.15
Regional measures						
Isthmus of cingulate	−0.12	−0.19	−0.51	−0.41	−0.22	−0.27
Precuneus	−0.11	−0.13	−0.68	−0.01	0.01	0.30
Supramarginal	−0.09	0.07	−0.65	−0.05	−0.48	−0.03
Temporal						
Temporal lobe	−0.05	−0.17	−**2.35**	−0.27	−1.11	0.16
Medial temporal lobe	−0.01	−0.10	−1.67	−0.19	−0.89	0.01
Lateral temporal lobe	−0.03	−0.12	−**2.05**	−0.24	−0.83	0.20
Regional measures						
Banks super temp sulcus	0.05	−0.08	−0.88	0.01	−0.28	0.43
Entorhinal cortex	−0.06	−0.06	−0.95	−0.29	−0.54	−0.10
Fusiform	0.06	−0.04	−1.29	−0.04	−0.70	0.02
Middle temporal	−0.11	−0.18	−1.59	−0.24	−0.58	−0.07
Parahippocampal	−0.40	−0.38	−1.54	−0.35	−1.25	−0.33
Superior temporal	0.02	−0.03	−1.31	−0.14	−0.95	0.57
Temporal pole	0.03	−0.03	−0.47	0.09	−0.18	0.47
Occipital						
Occipital lobe	0.02	−0.10	−0.71	0.11	−0.32	0.46
Regional measures						
Cuneus	0.06	0.04	−0.22	0.10	−0.01	0.28
Lateral occipital	−0.02	−0.09	−0.50	0.19	−0.23	0.31
Lingual	−0.02	0.00	−0.61	−0.04	−0.53	0.33
Pericalcarine	0.03	0.02	−0.24	0.17	−0.05	0.08
Insula	0.24	0.00	−0.96	−0.09	0.00	−0.42
Sensorimotor	−0.16	0.04	−0.80	−0.01	−0.15	−0.16
Cerebellum						
Cerebellar white matter	−0.21	−0.21	−**2.54**	−0.23	−0.71	0.07
Cerebellar gray mater	0.09	−0.02	−0.10	0.13	−0.29	−0.30
Deep structures						
Accumbens	0.15	0.16	−0.18	0.06	0.58	0.16
Amygdala	−0.07	−0.15	−0.99	−0.22	−0.41	−0.16
Caudate	0.00	−0.03	−0.73	0.14	0.38	0.52
Hippocampus	−0.30	−0.30	−1.53	−0.25	−1.55	−0.51
Pallidum	0.13	0.10	−0.77	−0.01	0.38	−0.14
Putamen	0.30	0.10	−0.77	−0.01	0.38	−0.02
Thalamus	−0.24	−0.21	−0.62	−0.11	−0.24	−0.61

*Note*: Bold font indicates observations with *p* < 0.05.

### 3.6 Mean β coefficient maps for quartiles of each subgroup

**FIGURE 5:**
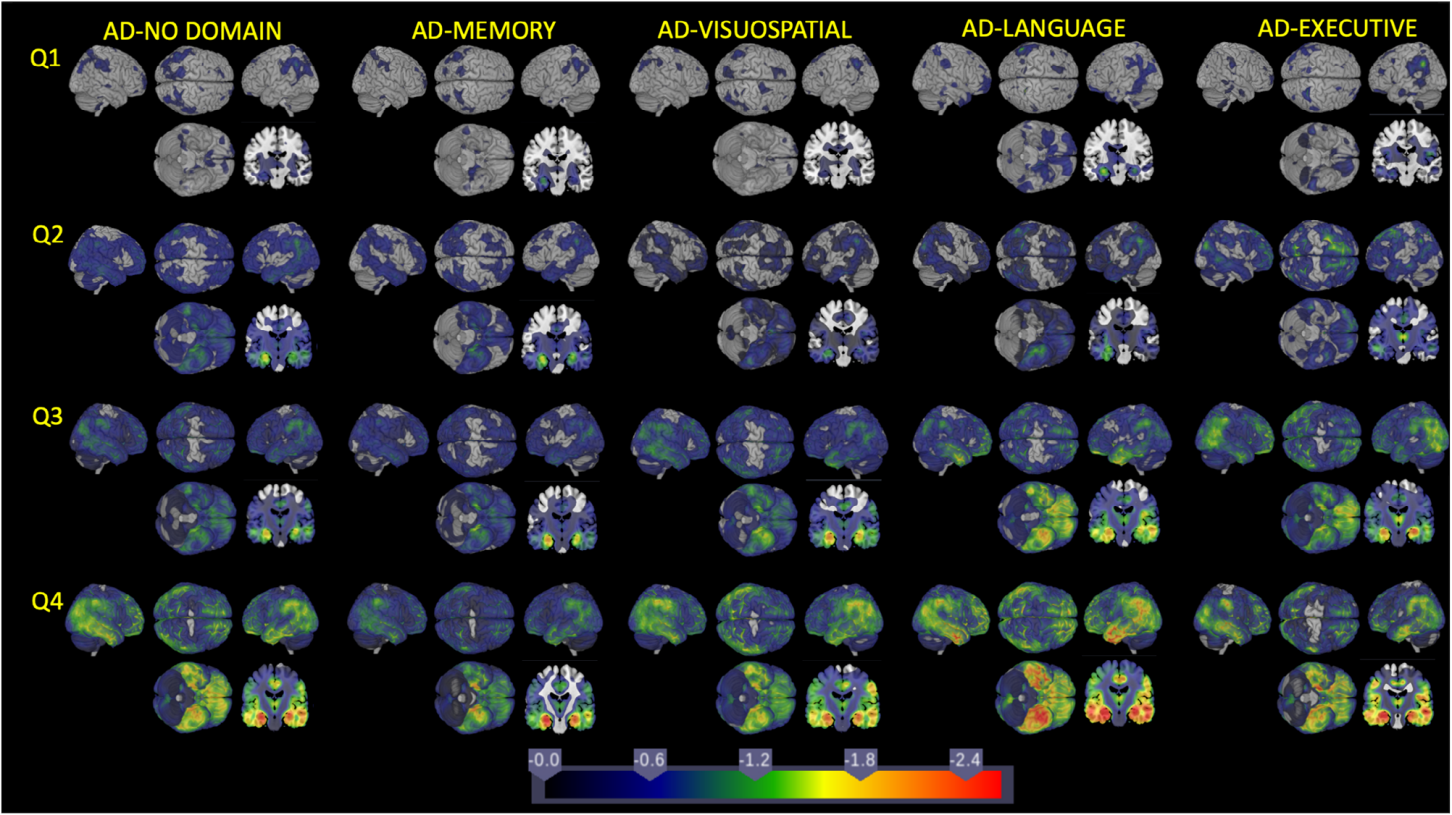
Mean gray matter findings compared to controls for each quartile defined by W‐score voxel counts for AD‐No Domain, AD‐Memory, AD‐Visuospatial, AD‐Language, and AD‐Executive.

We sorted each group by quartiles of voxels with W‐scores < −1.5. Table S3 shows thresholds between quartiles for each group. There was considerable overlap between the bottom quartiles in each group. In each AD subgroup, the 25th percentile ranged from 19,000 to 34,000 voxels, comparable to the controls’ 75th percentile (21,000 voxels). AD‐Memory had a restricted range of atrophy compared to other groups; the median (35,000 voxels) and 75th percentile (65,000 voxels) were lower than those of any other subgroup (medians 44,000–77,000, and 75th percentiles 78,000–122,000). Figure 5 shows β coefficient maps for each quartile for each subgroup compared to controls. In Figure 5, the left side of the coronal slices is to the left.

### 3.7 Comparison of cognitively defined and atrophy‐defined subgroups

We used MRI scans at the dementia visit to determine anatomically defined subgroups, as published.^11^ Table S4 and Figure S26 show comparisons with cognitively defined subgroups.

We used multinomial logistic regression to evaluate associations across groupings. We used relationships of anatomically defined subgroups for AD‐No Domains as reference. For each cognitively defined subgroup there are comparisons of the ratio of people with hippocampal sparing subtype (and limbic predominant subtype) to those for the typical AD subtype to those for the AD‐No Domain subgroup (Table S5). Compared to AD‐No Domain, the limbic‐predominant pattern was associated with higher AD‐Memory risk (relative risk ratio [RRR] 2.6, *p* = 4.6 × 10^−4^), and the hippocampal sparing pattern was associated with lower AD‐Memory risk (RRR 0.34, *p* = 0.0034).

Regarding the stability of anatomically defined subgroups over time, for people with incident dementia, we considered the enrollment and dementia visit scans (Figure S27). In all, 21% had a different anatomically defined subgroup at enrollment than at the dementia visit. In this subset, 18 of 47 (37%) with the hippocampal sparing subtype at enrollment had the typical AD subtype at dementia diagnosis, and 17 of 46 (37%) with the hippocampal sparing subtype at dementia diagnosis had the typical AD subtype at the first study visit (Figure S27). Similarly, 11 of 41 with the limbic‐predominant subtype at enrollment had the typical AD subtype at dementia diagnosis (27%), and 13 of 43 with the limbic‐predominant subtype at dementia diagnosis had the typical Alzheimer's subtype at enrollment (30%; Figure S27). Similarly, considering subgroups beginning at dementia diagnosis, there was considerable movement across subtypes from the dementia visit to the most recent follow‐up (Figure S28). In all, 19% had a different anatomically defined subgroup at the dementia visit and the most recent visit.

There were 248 people from our sample who had a subgroup defined by Poulakis et al.’s hierarchical Bayesian clustering approach applied to longitudinal imaging data.^12^ Tables S6 and S7 show that there were no significant associations between Poulakis et al.’s subgroups and cognitively defined subgroups.

## 4 DISCUSSION

We found different GMV patterns across cognitively defined subgroups. AD‐Memory had relative cortical sparing and severe medial temporal atrophy compared to other subgroups. AD‐Language had notable left‐greater‐than‐right atrophy, with statistically greater atrophy across much of the brain. These findings strongly replicate findings from a distinct study population.

Our prior study from Vrije Universiteit Medical Center (VUMC) Amsterdam also evaluated people with posterior cortical atrophy and with logopenic variant primary progressive aphasia. AD‐Language's regional findings resembled those of people with logopenic primary progressive aphasia, though there was more medial temporal lobe involvement in AD‐Language. In a formal voxel‐level comparison, AD‐Language's atrophy patterns were more highly correlated with logopenic primary progressive aphasia than with any other AD dementia subgroup. Similarly, AD‐Visuospatial's findings resembled those of posterior cortical atrophy, though there was more medial temporal involvement in AD‐Visuospatial. At the voxel level, AD‐Visuospatial had high correlations with posterior cortical atrophy.^10^

AD‐Language's left‐predominant atrophy pattern was consistent between ADNI and VUMC Amsterdam (Figure S23). We performed formal asymmetry analyses using average values over atlas‐defined ROIs. We found strong statistical support for asymmetry (Table 2).

Identifying ways to subdivide AD is an important recent area of research; the theory‐based cognitive approach we use is one of several candidate approaches. Notably, several anatomy‐based approaches – including the Poulakis et al. approach^12^ – in a first step average data from the left and right sides. While this reduces the number of regions considered, it makes it impossible to discern a subgroup with pronounced asymmetry.

Sarica et al.^38^ reviewed studies addressing asymmetry in people with AD compared with controls, including cortical thickness, cortical volumes, and cortical surface area, as well as white matter properties and functional connectivity. The details of these studies along with citations are provided in Supplementary Text 1. Most of these studies showed that structures on the left side had more atrophy than the right. Almost all of these studies have considered people with AD as a single group in their analyses. Several investigators suggest that findings of left lateralized AD pathology may be due to selection factors induced by cognitive assessments that emphasize language.^39^, ^40^, ^41^ While this could explain differences between people with AD dementia and controls, it cannot explain differences across cognitively defined subgroups of people with AD dementia.

A few studies have considered asymmetry and particular cognitive domains. Keilp et al. evaluated perfusion deficits and performance in specific cognitive domains.^42^ Derflinger et al. found that faster left hemisphere degeneration was associated with worse performance in language‐based cognitive tests across MCI and AD dementia.^39^ Frings et al. evaluated a sample of referred patients at a German specialty center and found that people with AD dementia with predominant language deficits exhibited more left‐lateralized Aβ burden based on [^11^C]PiB PET scans and hypometabolism based on [^18^F]FDG PET scans compared to people with AD dementia with predominant visuospatial impairment.^43^ Frings et al. did not have MRI scans on most of the people in their sample and highly recommended similar analyses in the ADNI dataset.^43^ The approaches to categorizing people with AD in Frings et al. is similar to that performed here and in our previous analyses of the Amsterdam University Medical Center cohort. The pattern of findings for those with relative language impairments reported in Frings et al. is similar to our findings. Intriguingly, in their analyses of tau deposition using tau PET scans, Vogel et al. identified one group with prominent left greater than right tau involvement in their discovery sample but not in a replication analysis.^36^

One implication of prominent asymmetric findings in a subset of people with typical late‐onset AD dementia is the possibility that neuropathology findings at autopsy may differ on the right and left sides. This has been evaluated with autopsy data. King et al. found asymmetric pTDP‐43 and plaque and tangle pathology in some people with clinical diagnoses of typical late‐onset AD dementia.^44^ Similarly, Stefanitis et al. found some left/right asymmetry in tau staining in some cases of AD dementia.^45^ There were insufficient cognitive data reported in those papers to comment on whether asymmetrical findings were associated with relative language impairments.

Both Vogel and colleagues^36^ and Poulakis and colleagues^12^ stressed the importance of addressing both stage and severity in subgrouping AD dementia. We do both. Like Poulakis and colleagues, we identify conversion to dementia as the anchoring time point. We used a different approach for severity with W‐scores to reflect overall atrophy. This is similar to approaches taken by Ossenkoppele and colleagues in other settings to compare typical late‐onset AD dementia with atypical AD including posterior cortical atrophy and logopenic primary progressive aphasia.^23^

Theory‐driven approaches differ in their treatment of minimal atrophy, or minimal levels of plaques and tangles. Murray et al. compared tangles in cortex and limbic regions among people with high Braak stages and classified people with minimal tangles in both as “typical AD.”^25^ Similarly, Risacher et al. did not separately consider individuals with minimal atrophy.^11^ Our approaches to pseudo‐progression, like those of Vogel et al. and the SuStaIn model approach applied to regional tau findings,^36^ suggest there may be overlap across subgroups at lower levels of overall atrophy, where it may be difficult to differentiate across subgroups based on imaging data. However, for quartiles with greater overall atrophy, subgroups differ in specific regions involved (Figure 5). We are analyzing ADNI's longitudinal imaging data to determine whether the pseudo‐progression suggested here, which by design represents between‐person differences, is confirmed by within‐person changes using longitudinal imaging data.

While we did not find overlap with Poulakis et al.’s subgroups (Tables S6 and S7), we found associations between AD‐Memory and the limbic‐predominant group defined using a theory‐driven imaging data approach (Table S5 and Figure S26).

Our findings should be considered along with the study's limitations. ADNI is a very large imaging study, but when we divided the cohort of people who developed AD into subgroups, some of those were small. ADNI excluded people with higher Hachinski ischemic scale scores, limiting the spectrum of vascular disease burden in the cohort. Whether these relationships hold in a less stringently selected population is uncertain, though the strong replication with a clinic‐based cohort without the vascular disease exclusion is reassuring. For people who enrolled in ADNI with MCI, ADNI required a memory deficit, possibly tipping the scales to higher proportions with AD‐Memory among those who convert to AD dementia. Indeed, rates in that subgroup were higher in ADNI than in other studies where we used the same approach for subtyping.^7^ ADNI has limited ethnic and racial diversity, and it will be important to replicate these analyses in more diverse samples. We took different approaches to those of Vogel et al. to account for differential disease severity across subgroups. We used W‐scores. This approach may actually overcorrect disease severity for AD‐Memory as that group has relative cortical sparing, meaning there are fewer cortical voxels at risk for atrophy in AD‐Memory compared to other groups (Table S3). Global atrophy as defined by W‐scores may not capture important levels of hippocampal involvement that may define different severity levels for AD‐Memory better than differential cortical involvement. We also limited these evaluations to cross‐sectional relationships. We are eager to learn whether patterns of progression suggested by Figure 5 are seen with individual‐level longitudinal data.

In conclusion, cognitively defined subgroups of people with late‐onset AD dementia have distinct atrophy patterns on structural MRI at the time of dementia diagnosis. Findings from the present analyses in ADNI are very similar to those we previously published in a different cohort. We found that the AD‐Memory group was characterized by lower GMV in the medial temporal lobe and less involvement elsewhere in the cortex compared to other subgroups. This description is analogous to the limbic‐predominant subgroup defined on the basis of structural imaging, and there was significant overlap between the AD‐Memory subgroup defined on the basis of relative cognitive impairments and the limbic‐predominant subgroup defined on the basis of structural imaging. There was distinct asymmetry with a left greater than right atrophy pattern in the AD‐Language group. Taken together, across different cohorts, we have found important contrasts between cognitively defined AD subgroups in terms of genetic findings,^7^ clinical findings,^8^ FDG‐PET findings,^9^ and now structural MRI differences in ADNI that replicate those we published from an independent cohort.^10^ These data add to the evidence base that suggests that typical late‐onset AD could reasonably be considered to be made up of distinct subgroups on the basis of relative impairments in cognition. Subsequent studies should determine whether this approach to categorizing people with AD may lead to important insights that result in personalized medicine approaches and/or discovery of therapeutics that may ameliorate the deleterious impacts of AD.

## CONFLICT OF INTEREST STATEMENT

Dr. Saykin receives support from multiple NIH grants (P30 AG010133, P30 AG072976, R01 AG019771, R01 AG057739, U19 AG024904, R01 LM013463, R01 AG068193, T32 AG071444, U01 AG068057, U01 AG072177, and U19 AG074879). He has also received support from Avid Radiopharmaceuticals, a subsidiary of Eli Lilly (in‐kind contribution of PET tracer precursor); Bayer Oncology (Scientific Advisory Board); Eisai (Scientific Advisory Board); Siemens Medical Solutions USA, Inc. (Dementia Advisory Board); NIH National Hearth, Lung, and Blood Institute (MESA Observational Study Monitoring Board); Springer‐Nature Publishing (Editorial Office Support as Editor‐in‐Chief, Brain Imaging and Behavior). No other authors have declarations of interest. Author disclosures are available in the Supporting information.

## CONSENT STATEMENT

All participants signed consent forms indicating their informed consent to participate in the study.

## Supporting information

Supporting Information

Supporting Information

## Data Availability

Data analyzed in this paper are available from the ADNI website (www.adni.loni.usc.edu). Analytic code for all steps is available on request, as are lists of participants assigned to each subgroup.
