# Supplementary material for: Cognitively defined Alzheimer's dementia subgroups have distinct atrophy patterns
Source: Alzheimers Dement. 2023 Dec 13;20(3):1739–52. doi: 10.1002/alz.13567 (PMC10984445; doi:10.1002/alz.13567)
Supplement: Supplementary file 1 — Supporting Information [file ALZ-20-1739-s001.docx]

**Supplemental materials**

**to accompany:**

**Cognitively-defined Alzheimer’s dementia subgroups have distinct atrophy patterns**

Paul K. Crane^1^*, Colin Groot,^2,3^ Rik Ossenkoppele,^2,3^ Shubhabrata Mukherjee,^1^ Seo-Eun Choi,^1^ Michael Lee,^1^ Phoebe Scollard,^1^ Laura E. Gibbons,^1^ R. Elizabeth Sanders,^1^ Emily Trittschuh,^4^ Andrew J. Saykin,^5,6^ Jesse Mez,^7^ Connie Nakano,^1^ Christine Mac Donald,^8^ Harkirat Sohi,^9^ for the Alzheimer’s Disease Neuroimaging Initiative**, and Shannon L. Risacher^5,6^

Table of Contents:

| Supplementary Fig. 1 | Effects of applying sample inclusion and exclusion criteria on samples available for analyses | 3 |
| --- | --- | --- |
| Supplementary Fig. 2 | Schematic of subgrouping approach | 4 |
| ***Comparisons of each cognitively defined subgroup with controls (Supplementary Fig. 3-14)*** | | |
| Supplementary Fig. 3 | Voxel based morphometry (VBM) p value findings for AD-No Domain compared with cognitively normal elderly controls | 5 |
| Supplementary Fig. 4 | VBM β coefficient findings for AD-Memory compared with cognitively normal elderly controls | 6 |
| Supplementary Fig. 5 | VBM p value findings for AD-No Domains compared with cognitively normal elderly controls | 6 |
| Supplementary Fig. 6 | VBM β coefficient findings for AD-Visuospatial compared with cognitively normal elderly controls | 7 |
| Supplementary Fig. 7 | VBM p value findings for AD-Visuospatial compared with cognitively normal elderly controls | 7 |
| Supplementary Fig. 8 | VBM β coefficient findings for AD-Executive compared with cognitively normal elderly controls | 8 |
| Supplementary Fig. 9 | VBM p value findings for AD-Executive compared with cognitively normal elderly controls | 8 |
| Supplementary Fig. 10 | VBM β coefficient findings for AD-Multiple domains compared with cognitively normal elderly controls | 9 |
| Supplementary Fig. 11 | VBM p value findings for AD-Multiple domains compared with cognitively normal elderly controls | 9 |
| Supplementary Fig. 12 | VBP p value findings for AD-Language compared with cognitively normal elderly controls | 10 |
| Supplementary Fig. 13 | VBM β coefficient findings for all subgroups compared with cognitively normal elderly controls | 10 |
| Supplementary Fig. 14 | VBM p value findings for all subgroups compared with cognitively normal elderly controls | 11 |
| ***Comparisons between AD-Memory and each of the other subgroups (Supplementary Fig. 15-20)*** | | |
| Supplementary Fig. 15 | VBM p value findings for AD-Memory compared with AD-No Domains | 11 |
| Supplementary Fig. 16 | Left sided temporal and hippocampal regions for AD-Memory compared with AD-Language | 12 |
| Supplementary Fig. 17 | VBM β coefficient findings for AD-Memory compared with AD-Visuospatial | 13 |
| Supplementary Fig. 18 | VBP p value findings for AD-Memory compared with AD-Visuospatial | 14 |
| Supplementary Fig. 19 | VBM β coefficient findings for AD-Memory compared with AD-Executive | 15 |
| Supplementary Fig. 20 | VBM β coefficient findings for AD-Memory compared with AD-Multiple Domains | 16 |
| ***Comparisons of findings from ADNI and VUMC-Amsterdam (Supplementary Fig. 21-25)*** | | |
| Supplementary Fig. 21 | VBM findings for AD-No Domains compared to cognitively normal elderly controls for ADNI and VUMC-Amsterdam late-onset AD | 17 |
| Supplementary Fig. 22 | VBM findings for AD-Memory compared to cognitively normal elderly controls for ADNI and VUMC-Amsterdam late-onset AD | 18 |
| Supplementary Fig. 23 | VBM findings for AD-Language compared to cognitively normal elderly controls for ADNI and VUMC-Amsterdam late-onset AD | 19 |
| Supplementary Fig. 24 | VBM findings for AD-Visuospatial compared to cognitively normal elderly controls for ADNI and VUMC-Amsterdam late-onset AD | 20 |
| Supplementary Fig. 25 | VBM findings for AD-Executive compared to cognitively normal elderly controls for ADNI and VUMC-Amsterdam late-onset AD | 21 |
| ***Full results of asymmetry analyses (Supplementary Tables 1-2)*** | | |
| Supplementary Table 1 | Supplementary Table 1: Asymmetry results in z-score units, including left-handed individuals. | 22 |
| Supplementary Table 2 | Supplementary Table 2: Asymmetry results in z-score units, limited to right-handed people. | 23 |
| Supplementary Table 3 | Thresholds between quartiles of overall atrophy defined by the count of voxels with W-scores less than -1.5 for each subgroup | 25 |
| ***Comparisons of cognitively-defined subgroups to anatomically-defined subgroups (Supplementary Tables 4 and 5, Supplementary Fig. 26)*** | | |
| Supplementary Table 4 | Cognitively-defined and anatomically-defined subgroups as in Risacher et al. [1] | 25 |
| Supplementary Fig. 26 | Comparison of cognitively-defined subgroups and anatomically-defined subgroups. | 26 |
| Supplementary Table 5 | Multinomial logistic regression results of relative risk ratios associated with limbic predominant and hippocampal sparing vs. typical AD | 27 |
| ***Stability of atrophy-defined subgroups over time (Supplementary Fig. 27-28 and Supplementary Tables 6 and 7)*** | | |
| Supplementary Fig. 27 | Stability of atrophy-defined subgroups from study entry to Alzheimer’s dementia diagnosis for people with incident Alzheimer’s dementia (n=287) | 28 |
| Supplementary Fig. 28 | Stability of atrophy-defined subgroups from Alzheimer’s dementia diagnosis to the most recent scan for people with prevalent or incident Alzheimer’s dementia (n=416) | 29 |
| Supplementary Table 6 | Cognitively defined subgroups compared with subgroups from Poulakis et al. [2] | 30 |
| Supplementary Table 7 | Multinomial logistic regression results of relative risk ratios associated with minimal atrophy vs. limbic predominant | 30 |
| Supplementary Text 1 | More detailed discussion of asymmetry literature | 30 |
| **References** |  | 31 |

Supplementary Fig. 1. Effects of applying sample inclusion and exclusion criteria on samples available for analyses

| 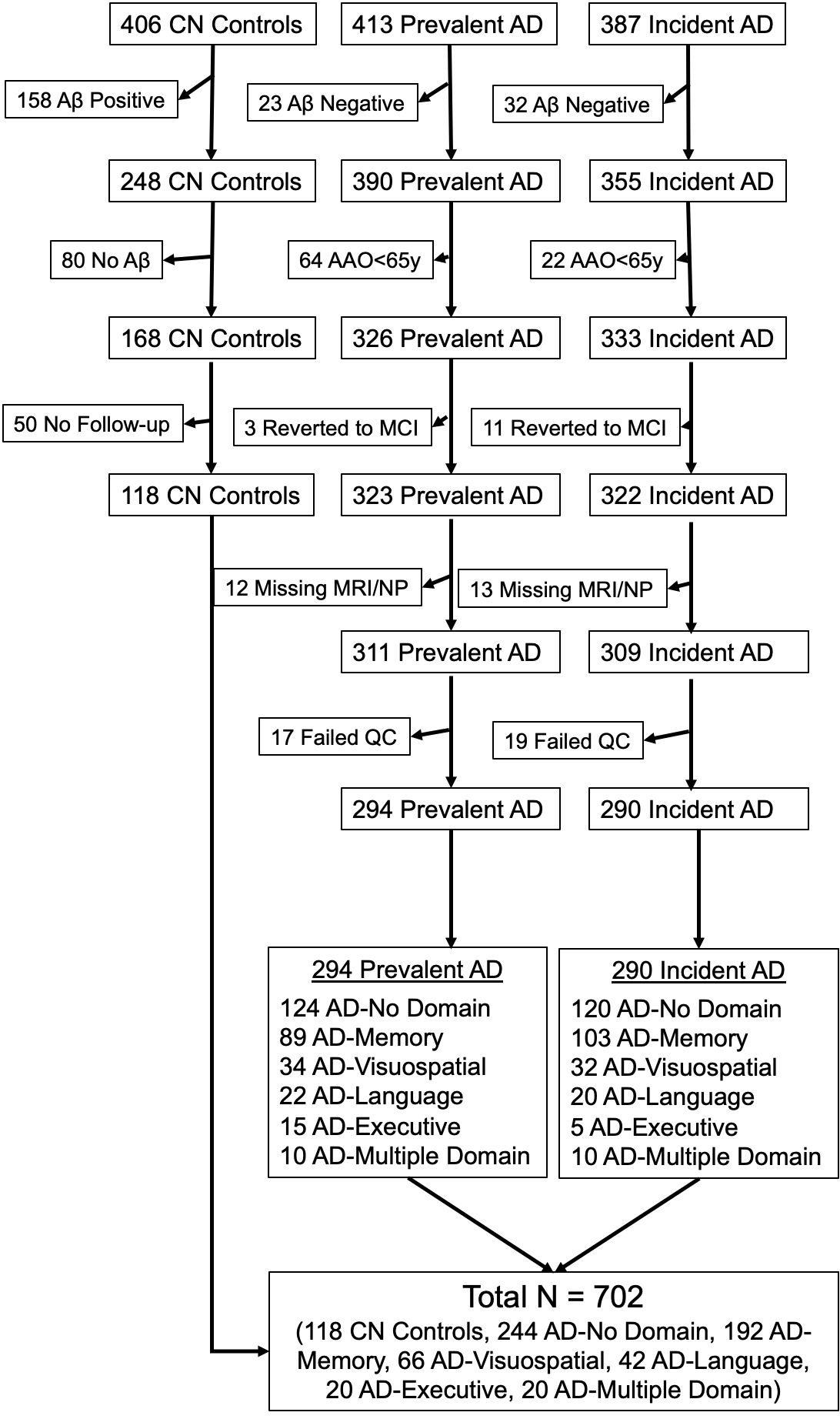 |
| --- |

* Abbreviations: AD = Alzheimer’s disease dementia; CN = cognitively normal; AAO = age at onset; MCI = mild cognitive impairment; NP = neuropsychological testing data; QC = quality control.

Supplementary Fig. 2 – Schematic of subgrouping approach

| **Person 1** | **Person 2** |
| --- | --- |
| 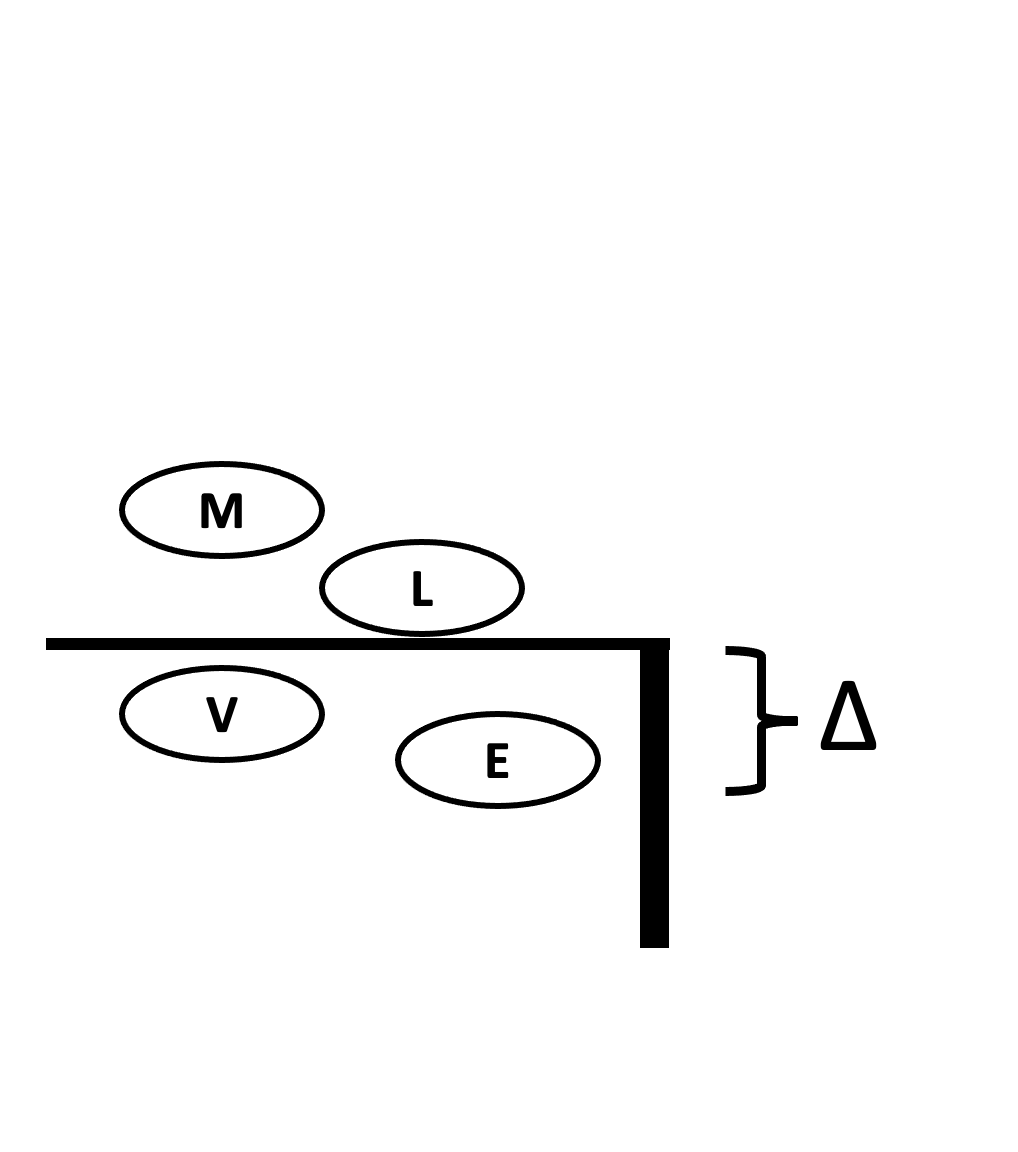 | 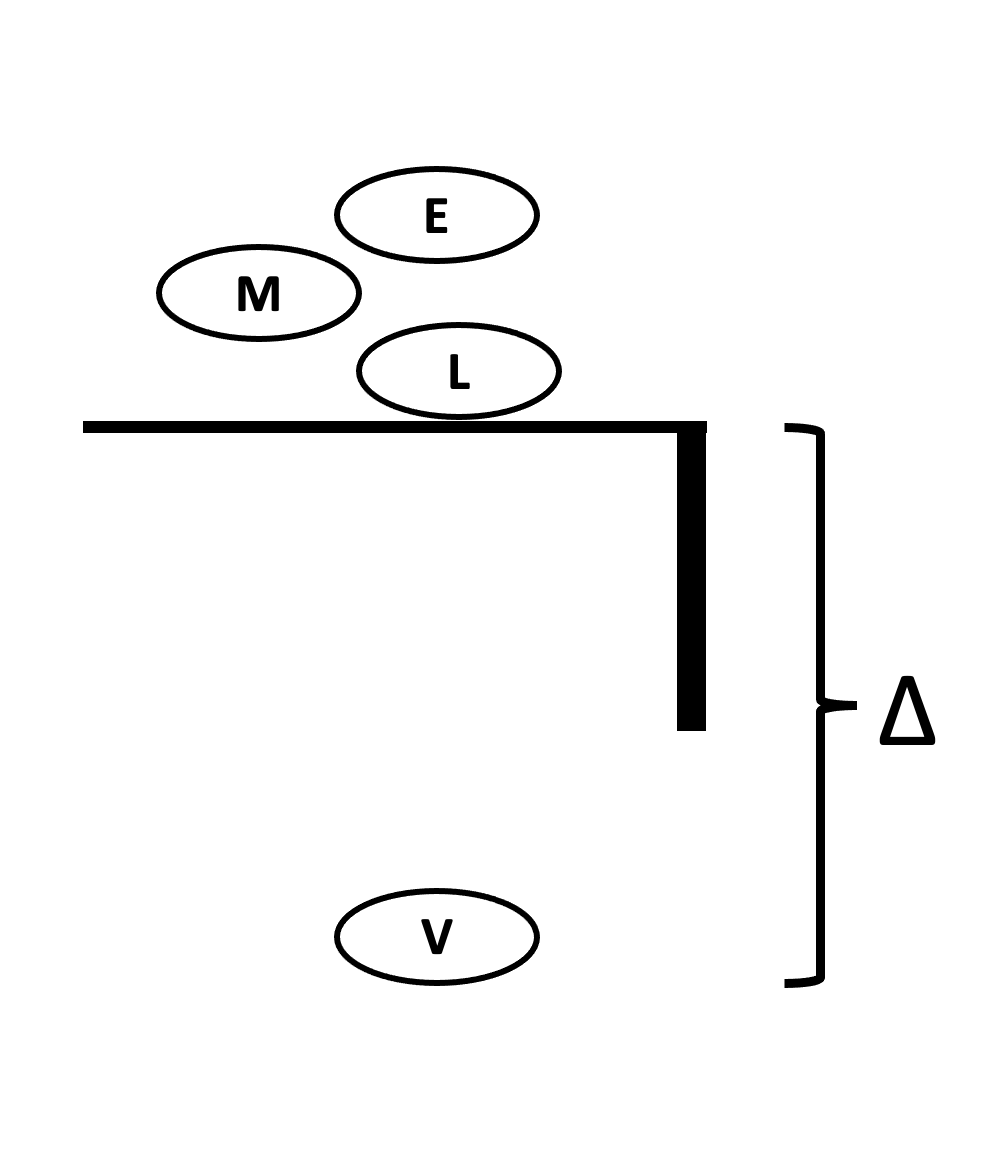 |

Ovals depict scores ranging from low (bottom) to high (top) for memory (M), executive functioning (E), language (L) and visuospatial (V) at the time of Alzheimer’s dementia diagnosis, derived from ADNI’s cognitive battery. The horizontal line depicts each person’s average across M, E, L and V. The vertical line indicates the critical value. The delta indicates the difference between the person’s average score and their lowest score. Person 1’s lowest score is E (executive functioning) but the difference between their average score and their executive functioning score is less than the critical value, so they are classified as AD-No Domains. Person 2’s lowest score is V (visuospatial), which is quite a bit lower than their average score, and that difference is larger than the critical value, so they are classified as AD-Visuospatial.

Supplementary Fig. 3. Voxel based morphometry (VBM) p value findings for AD-No Domain compared with cognitively normal elderly controls

| 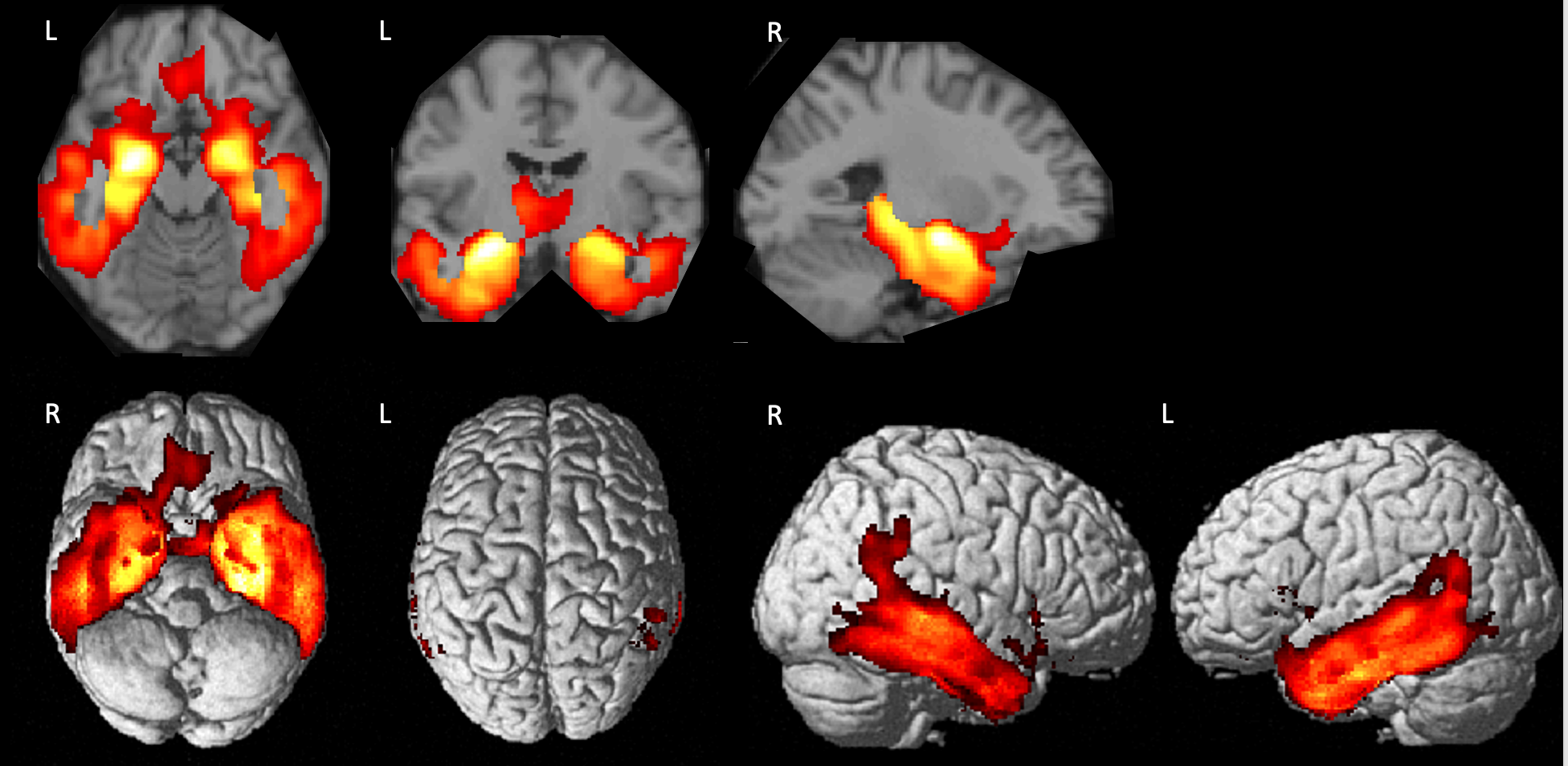 |
| --- |
| 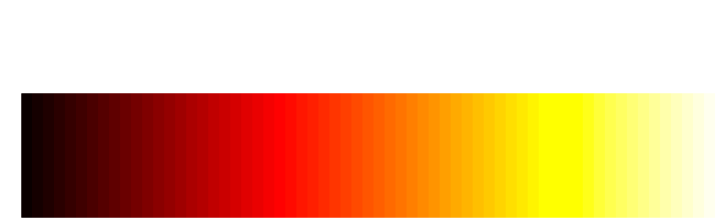 |

Supplementary Fig. 4. VBM β coefficient findings for AD-Memory compared with cognitively normal elderly controls

| 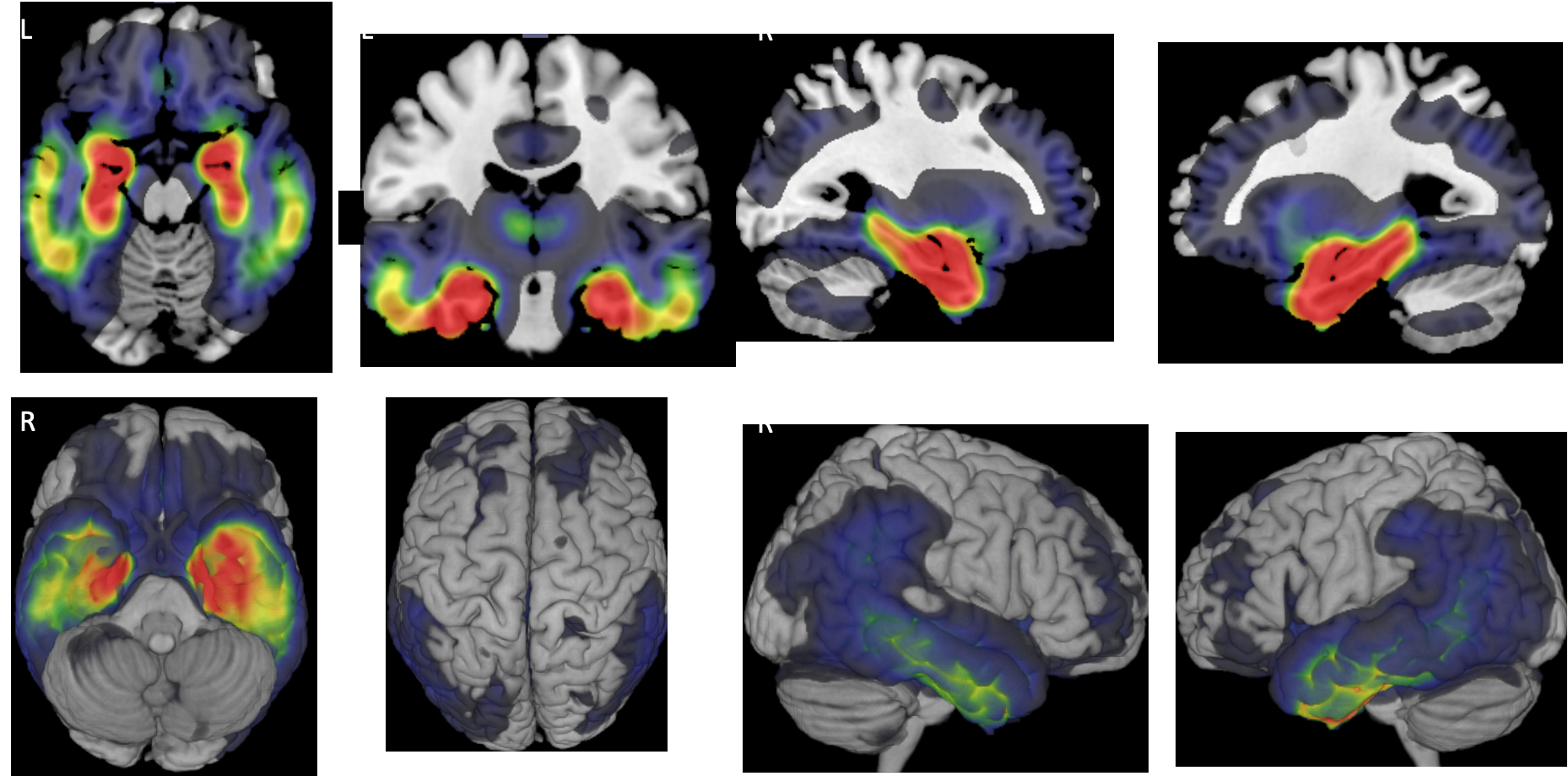 |
| --- |
| 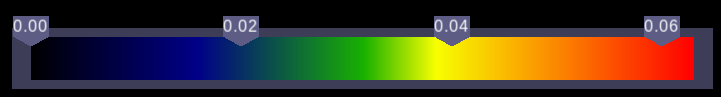 |

Supplementary Fig. 5. VBM p value findings for AD-Memory compared with cognitively normal elderly controls

| 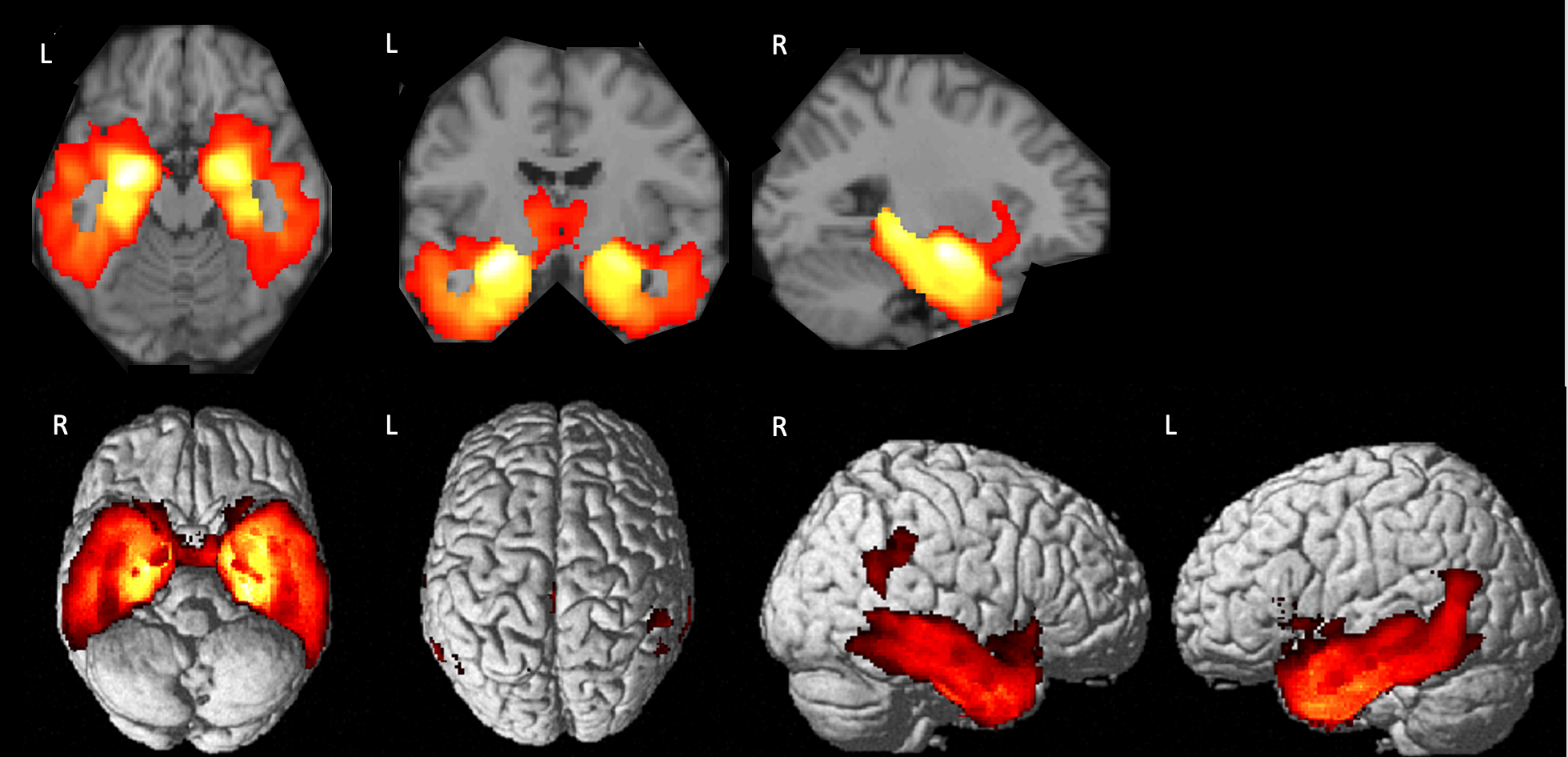 |
| --- |
| 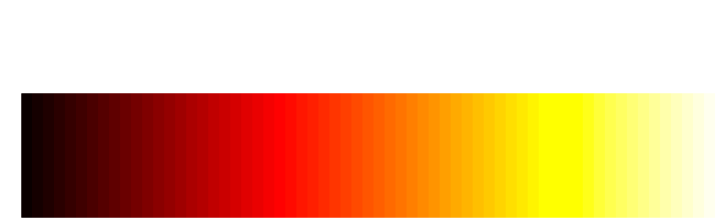 |

Supplementary Fig. 6. VBM β coefficient findings for AD-Visuospatial compared with cognitively normal elderly controls

| 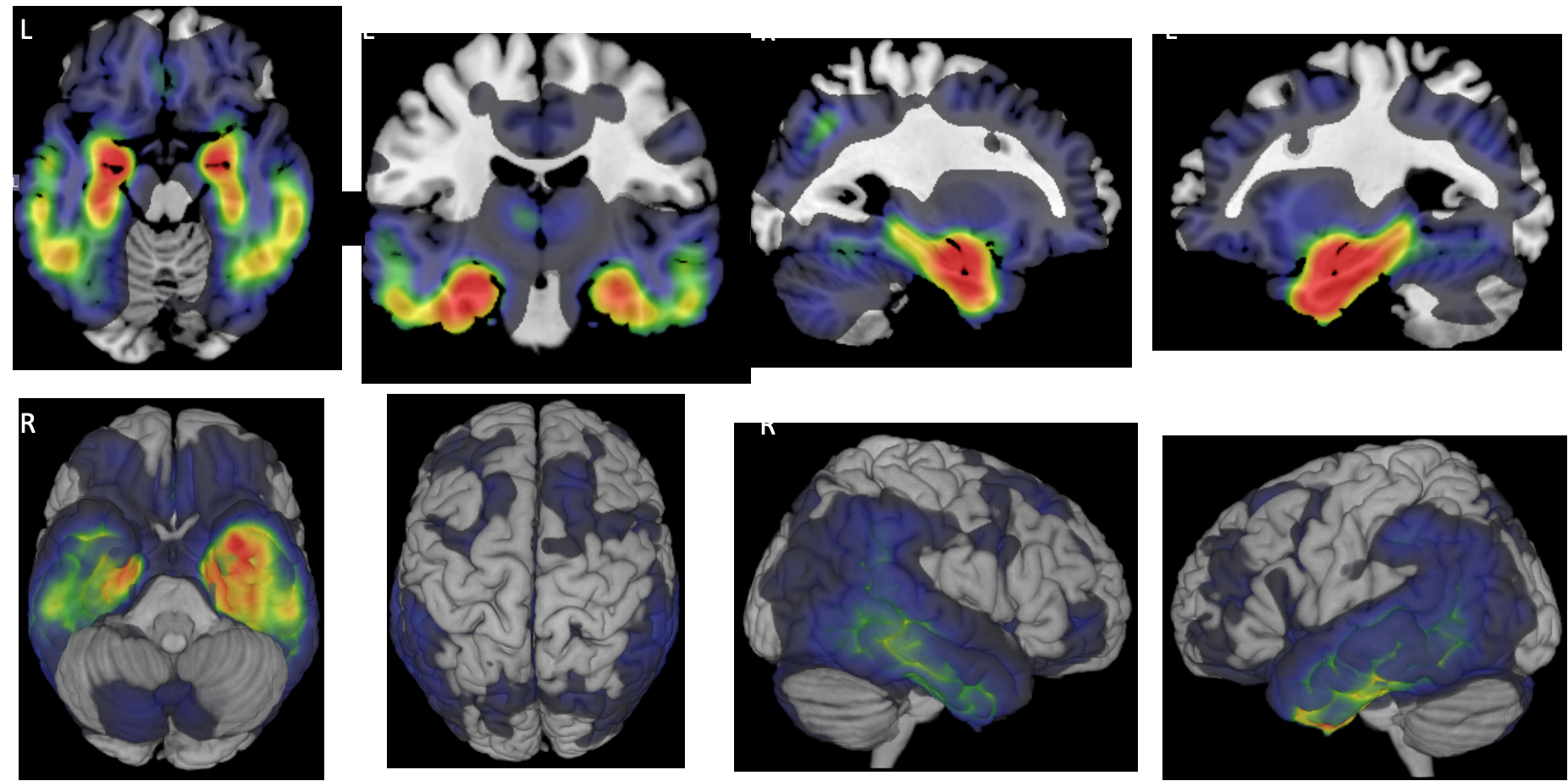 |
| --- |
| 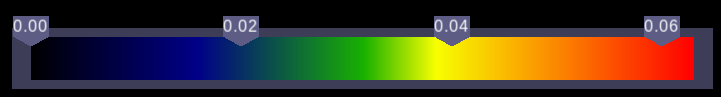 |

Supplementary Fig. 7. VBM p value findings for AD-Visuospatial compared with cognitively normal elderly controls

| 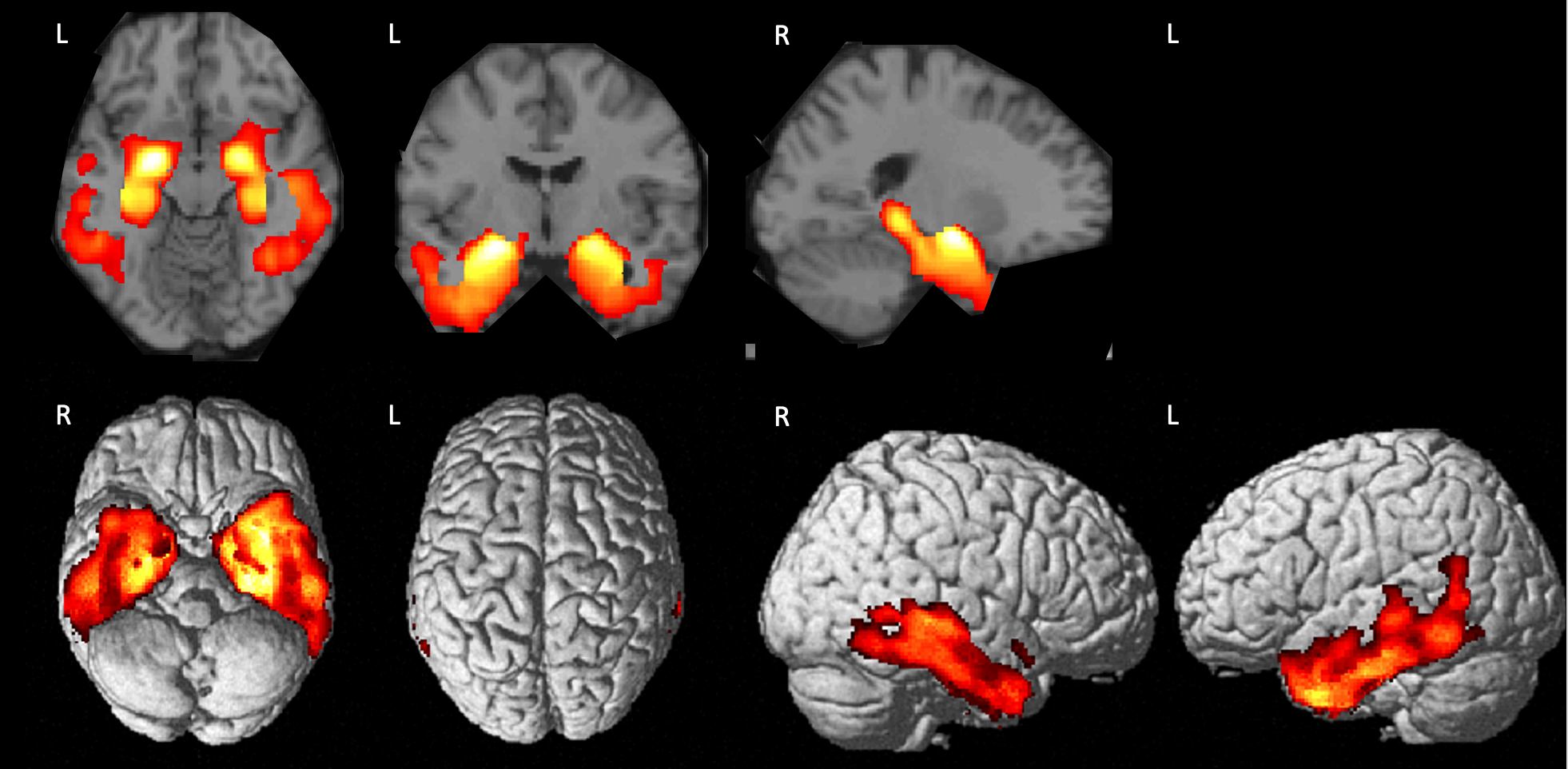 |
| --- |
| 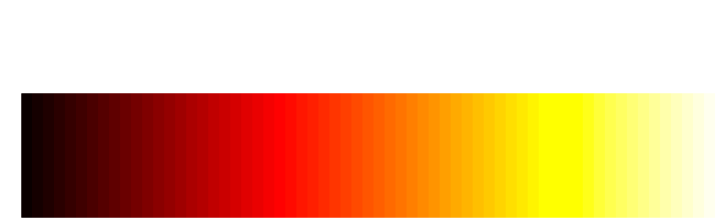 |

Supplementary Fig. 8. VBM β coefficient findings for AD-Executive compared with cognitively normal elderly controls

| 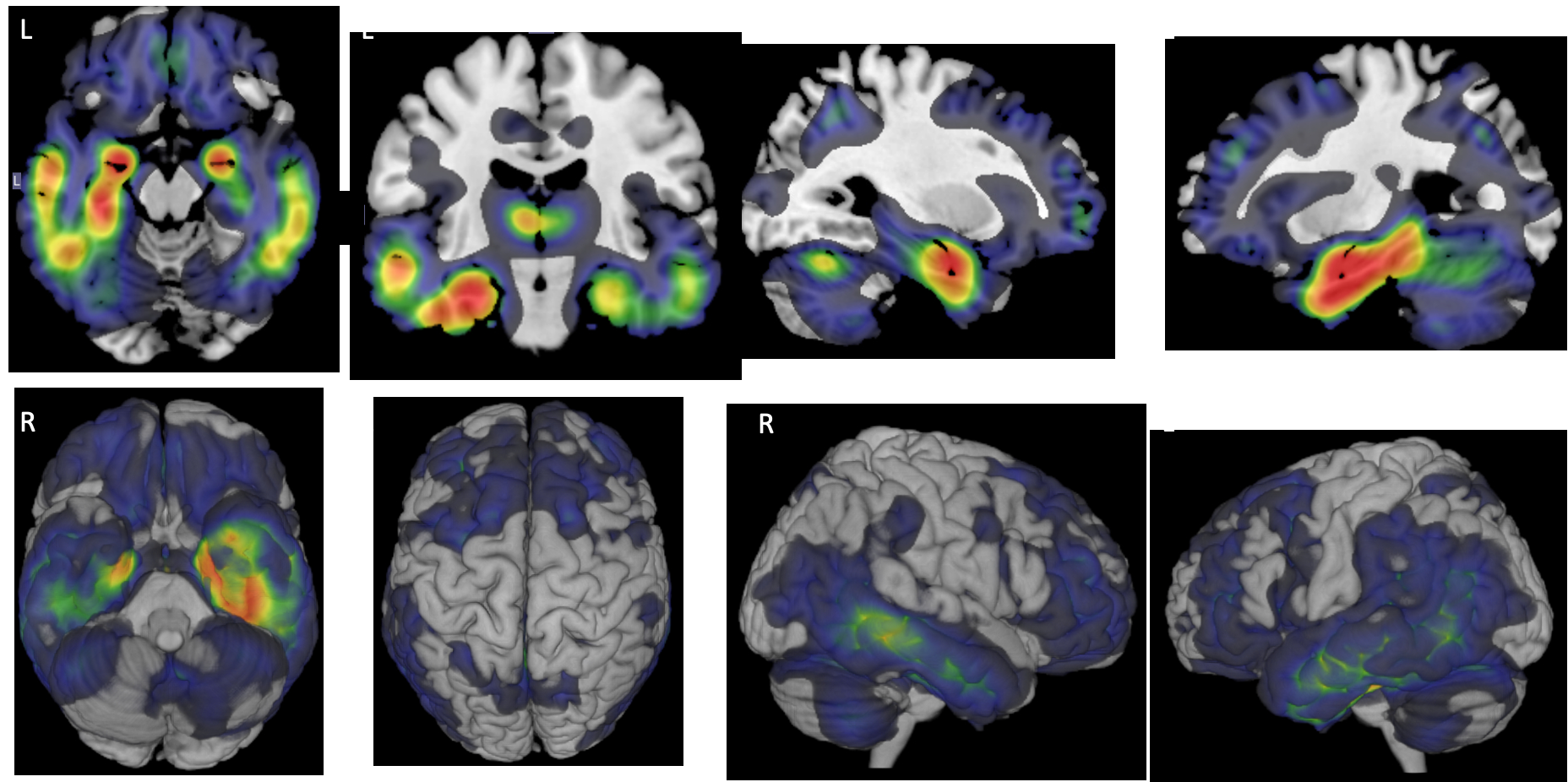 |
| --- |
| 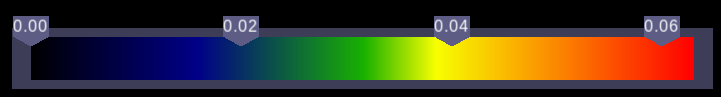 |

Supplementary Fig. 9. VBM p value findings for AD-Executive compared with cognitively normal elderly controls

| 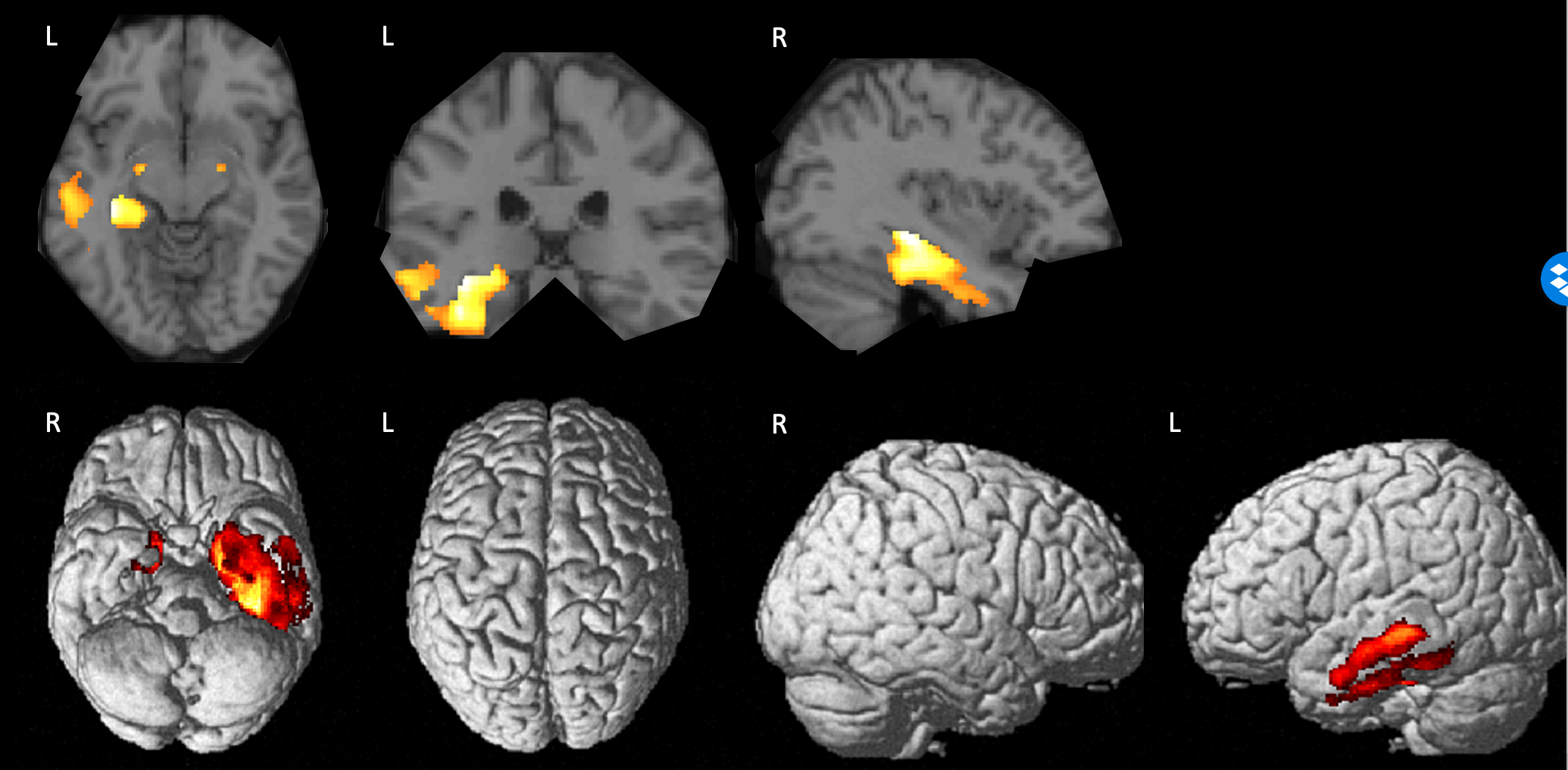 |
| --- |
| 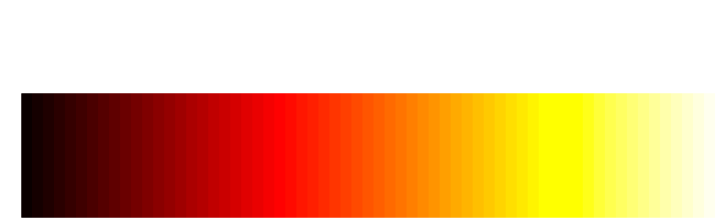 |

Supplementary Fig. 10. VBM β coefficient findings for AD-Multiple Domains compared with cognitively normal elderly controls

| 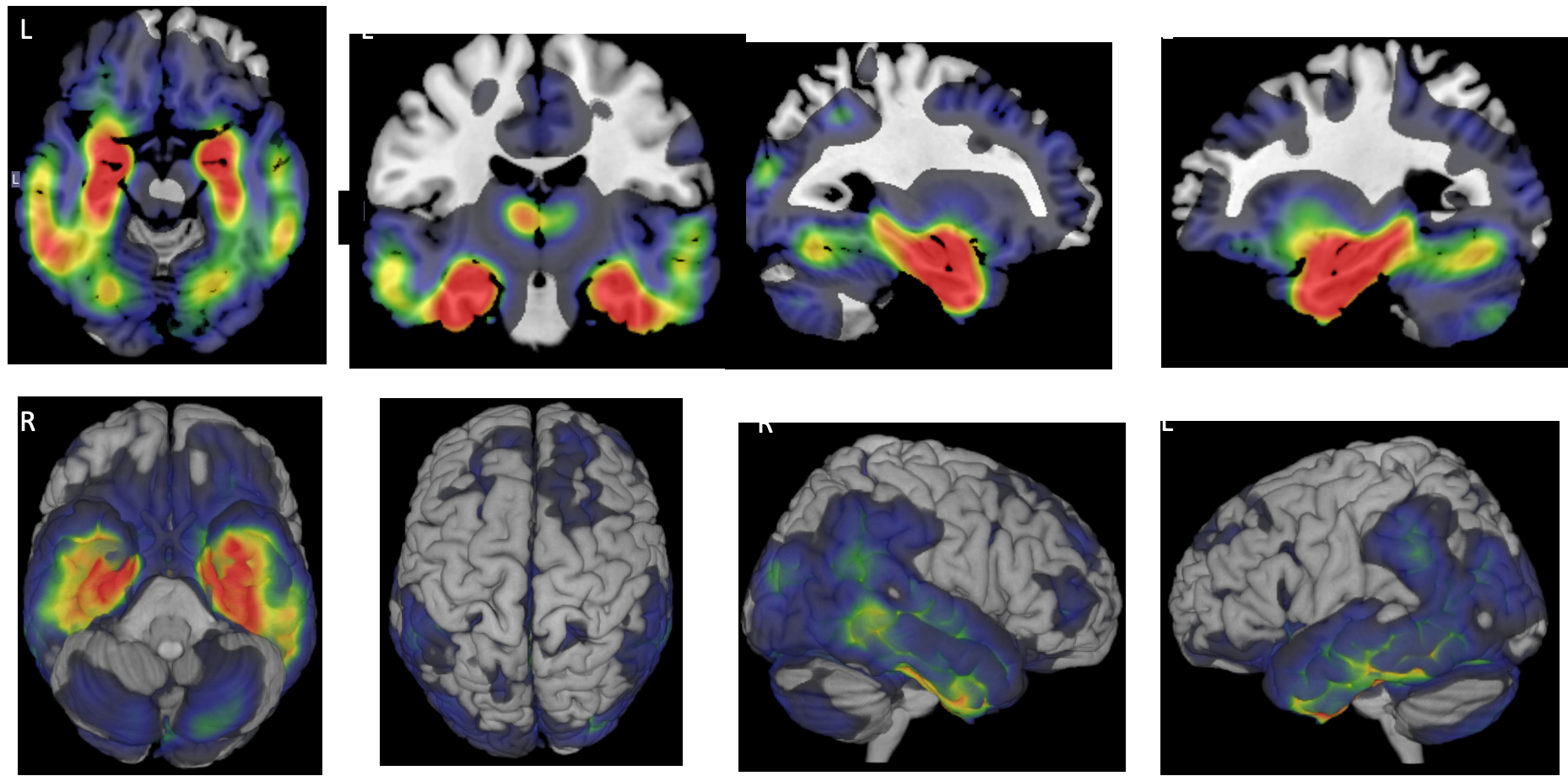 |
| --- |
| 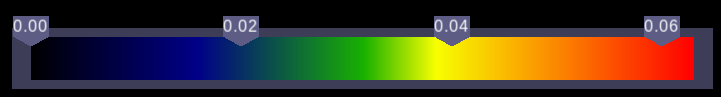 |

Supplementary Fig. 11. VBM p value findings for AD-Multiple Domains compared with cognitively normal elderly controls

| 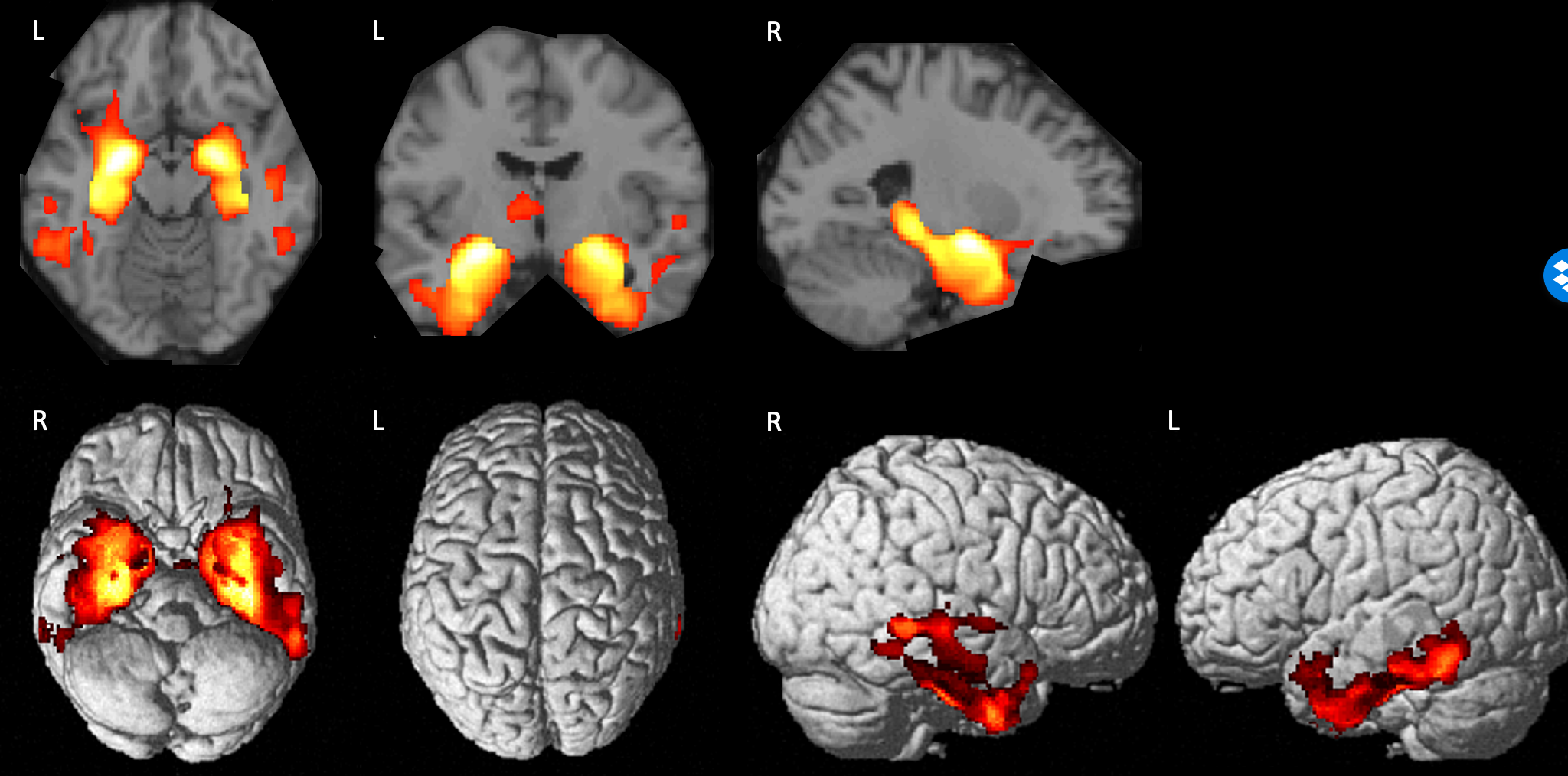 |
| --- |
| 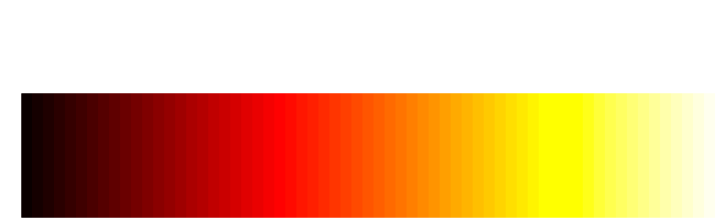 |

Supplemental Figure 12. VBM p value findings for AD-Language compared with cognitively normal elderly controls

| 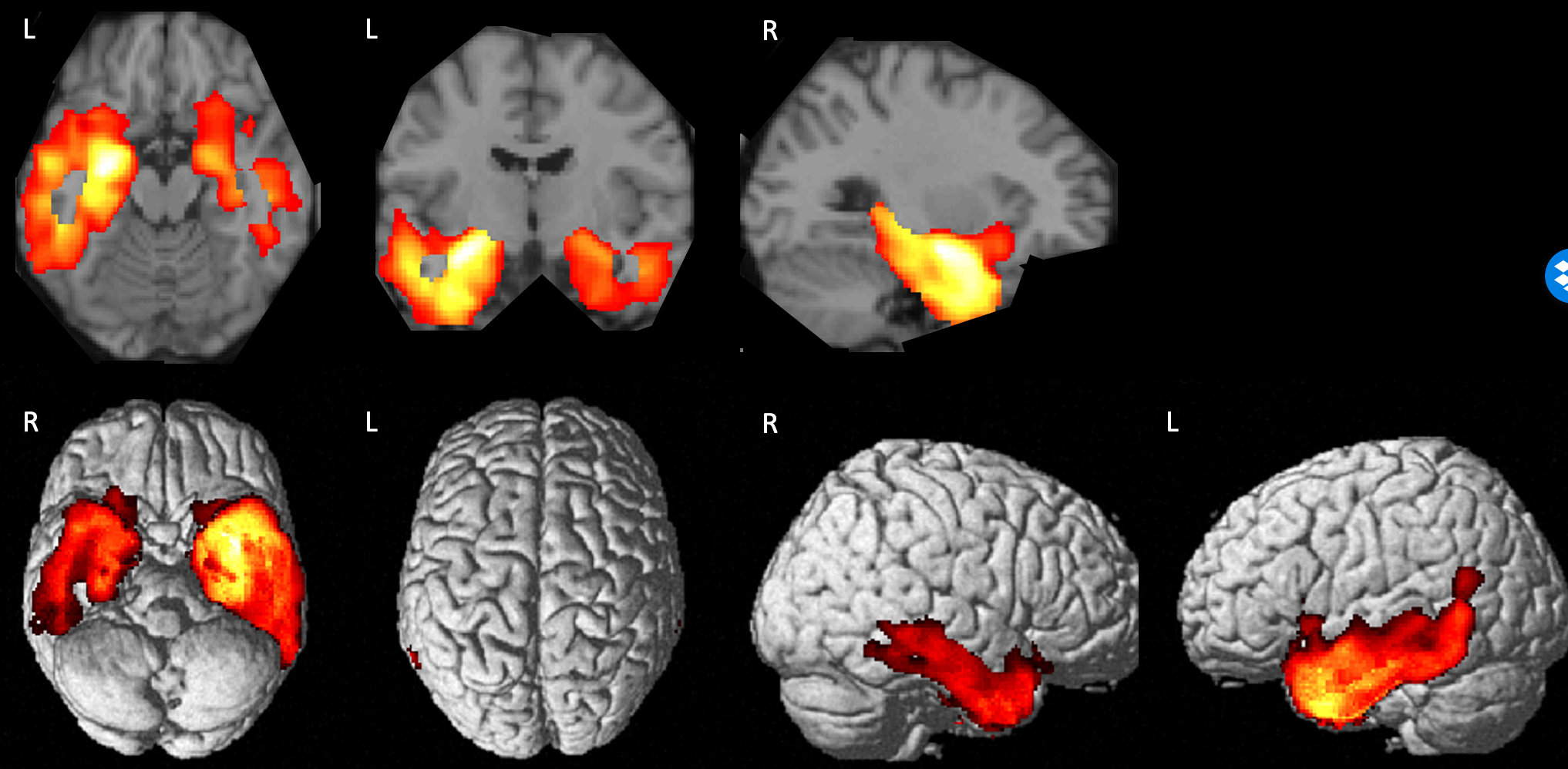 |
| --- |
| 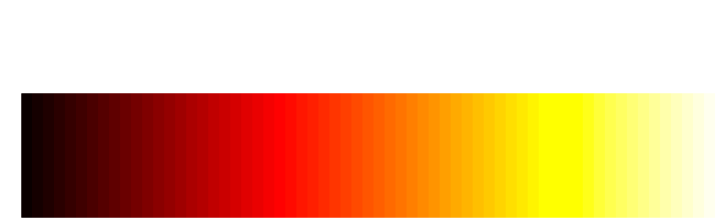 |

Supplemental Figure 13. VBM β coefficient findings for all subgroups compared to cognitively normal elderly controls


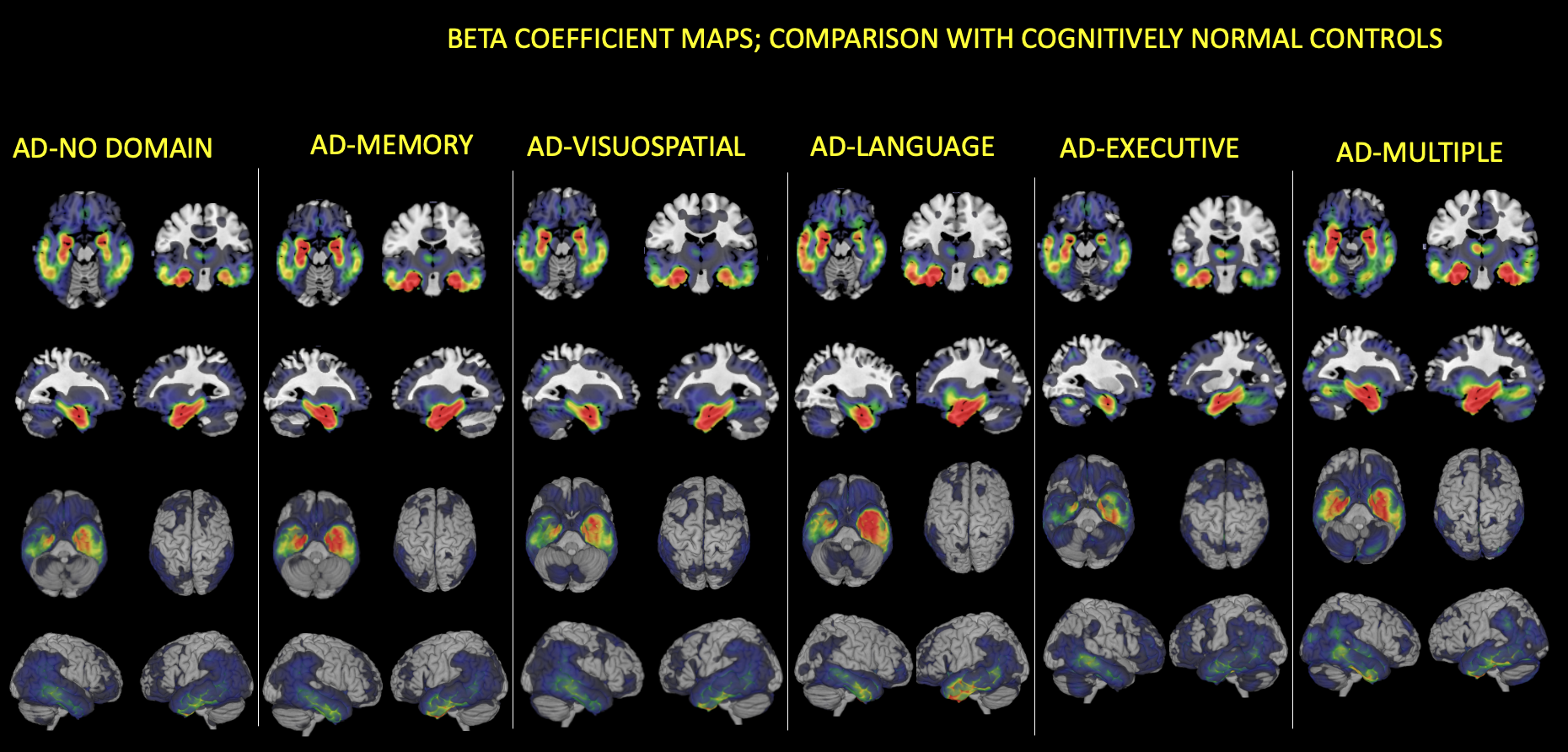


Supplementary Fig 14. VBM p value findings for all subgroups compared to cognitively normal elderly controls.


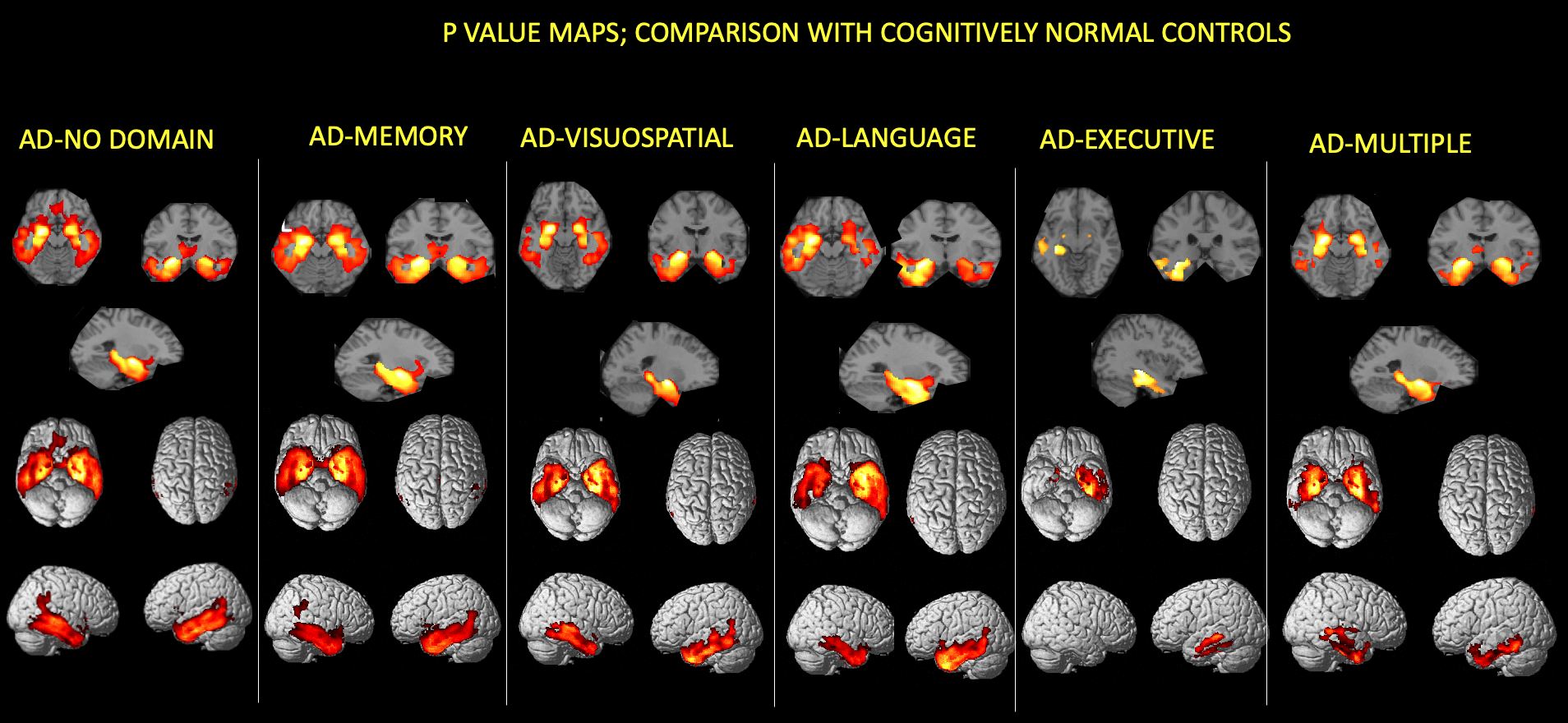


Supplementary Fig. 15. VBM p value findings for AD-No Domain compared with AD-Memory

| 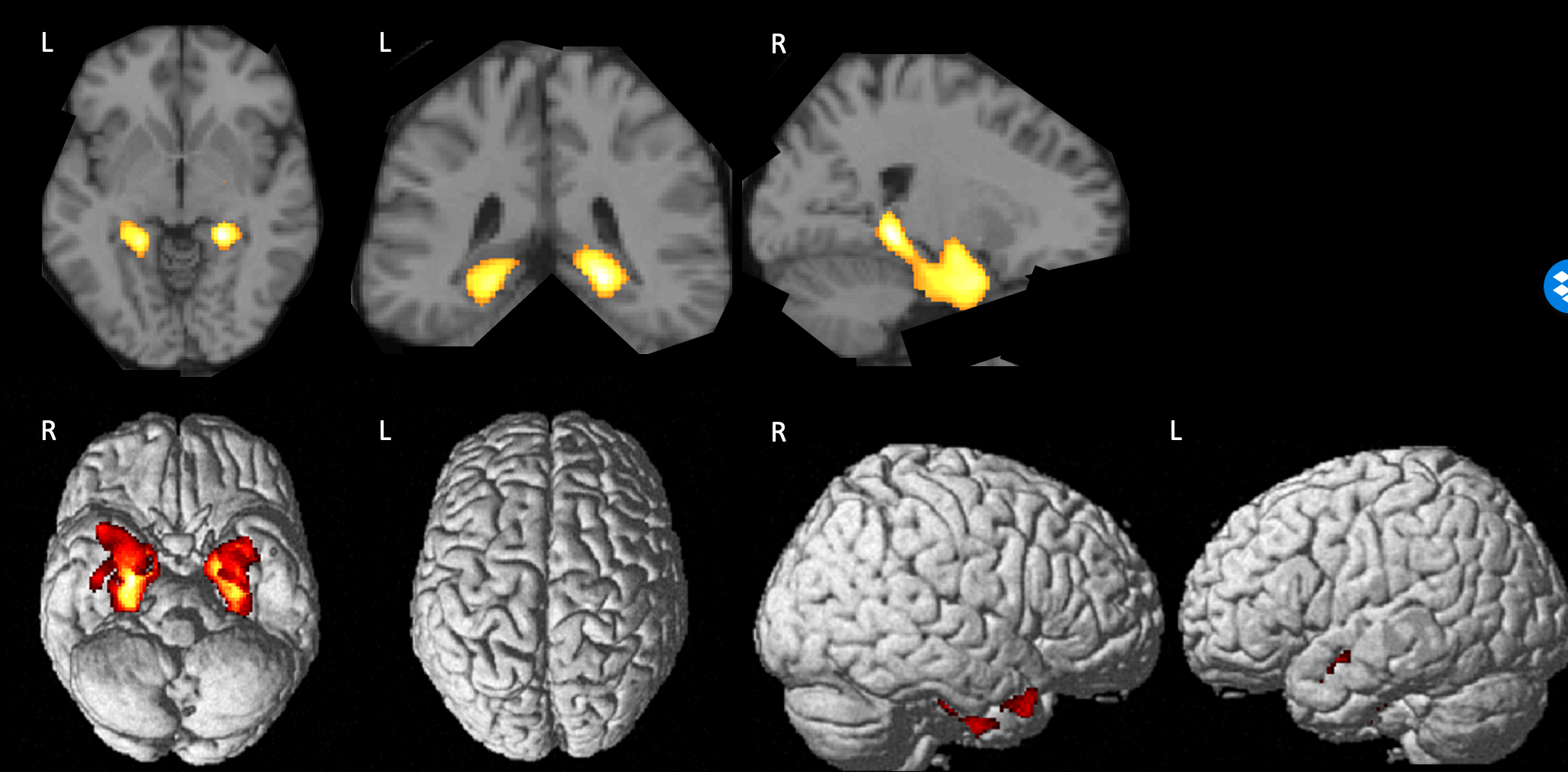 |
| --- |
| 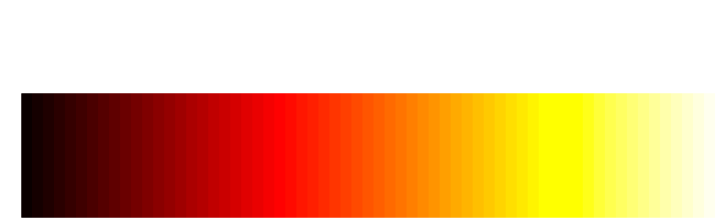 |

Supplementary Figure 16. Left sided temporal and hippocampal regions for AD-Memory and AD-Language (enlargements of β coefficient comparisons with cognitively normal elderly controls)*

| 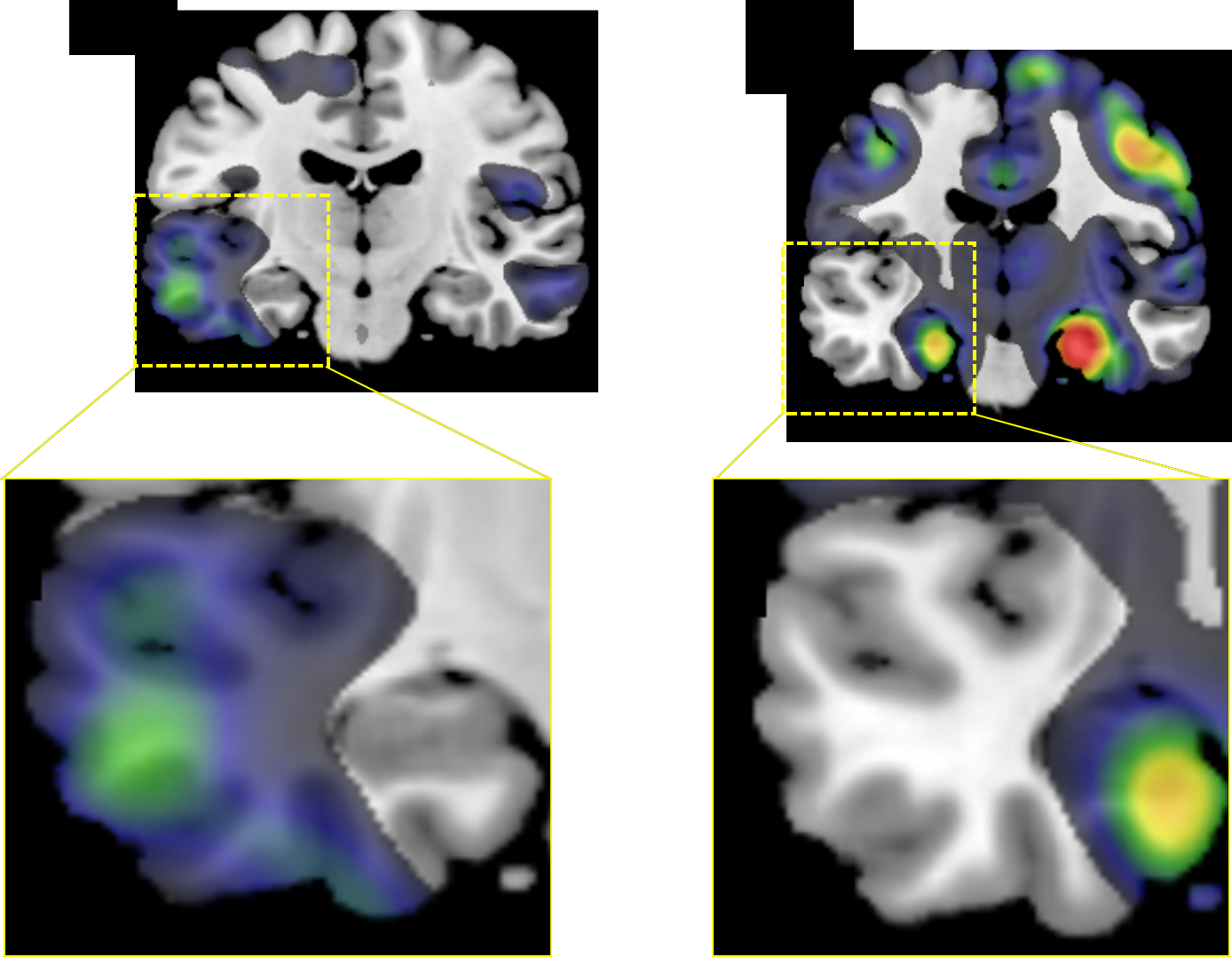 |
| --- |

* The left side shows an enlargement of voxels in which the amount of atrophy for the AD-Language group is greater than the atrophy for the AD-Memory group. The right side shows an enlargement of voxels in which the amount of atrophy for the AD-Memory group is greater than the atrophy for the AD-Language group.

Supplementary Fig. 17. VBM β coefficient findings for AD-Memory compared with AD-Visuospatial

| A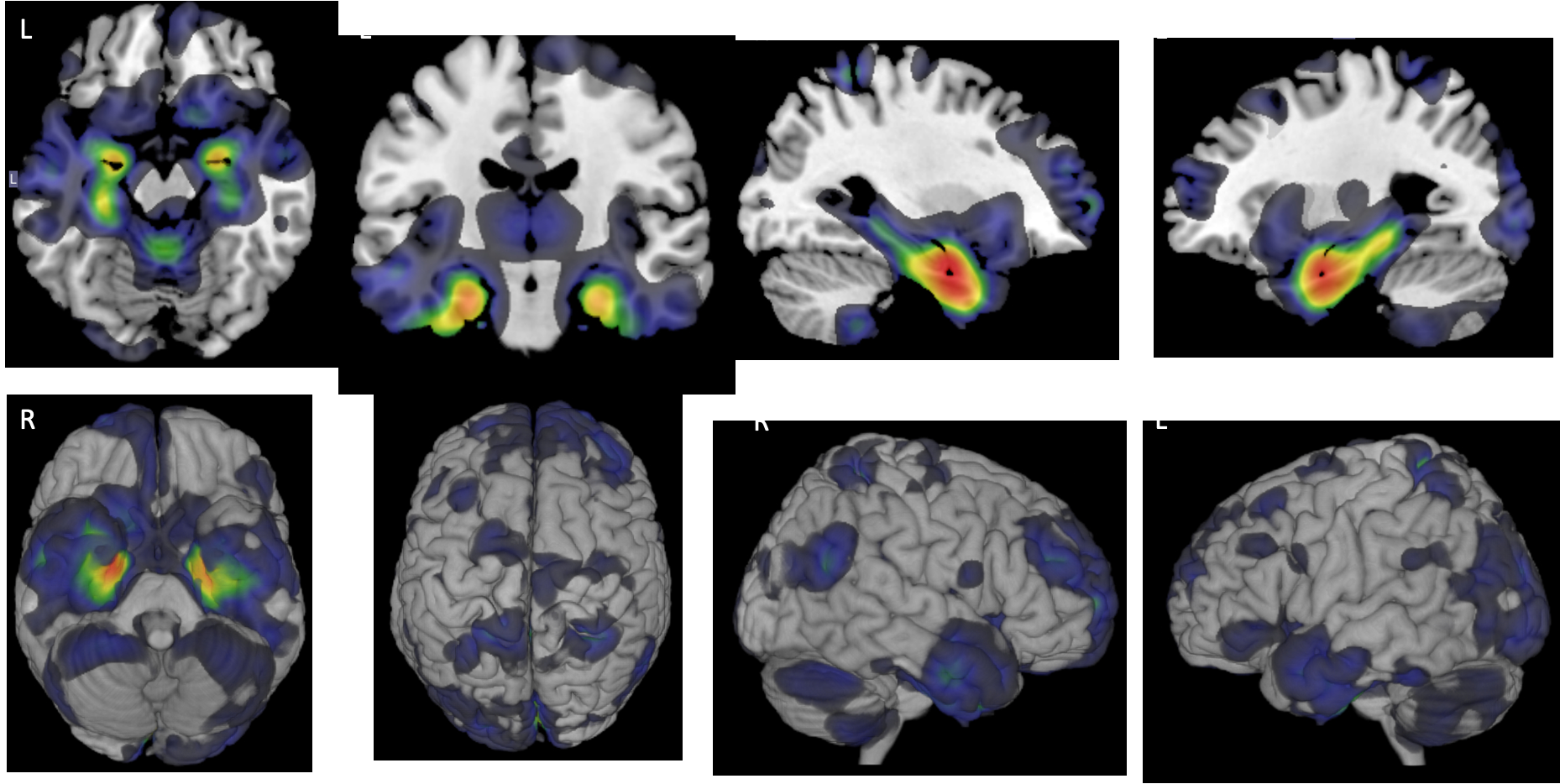 |
| --- |
| B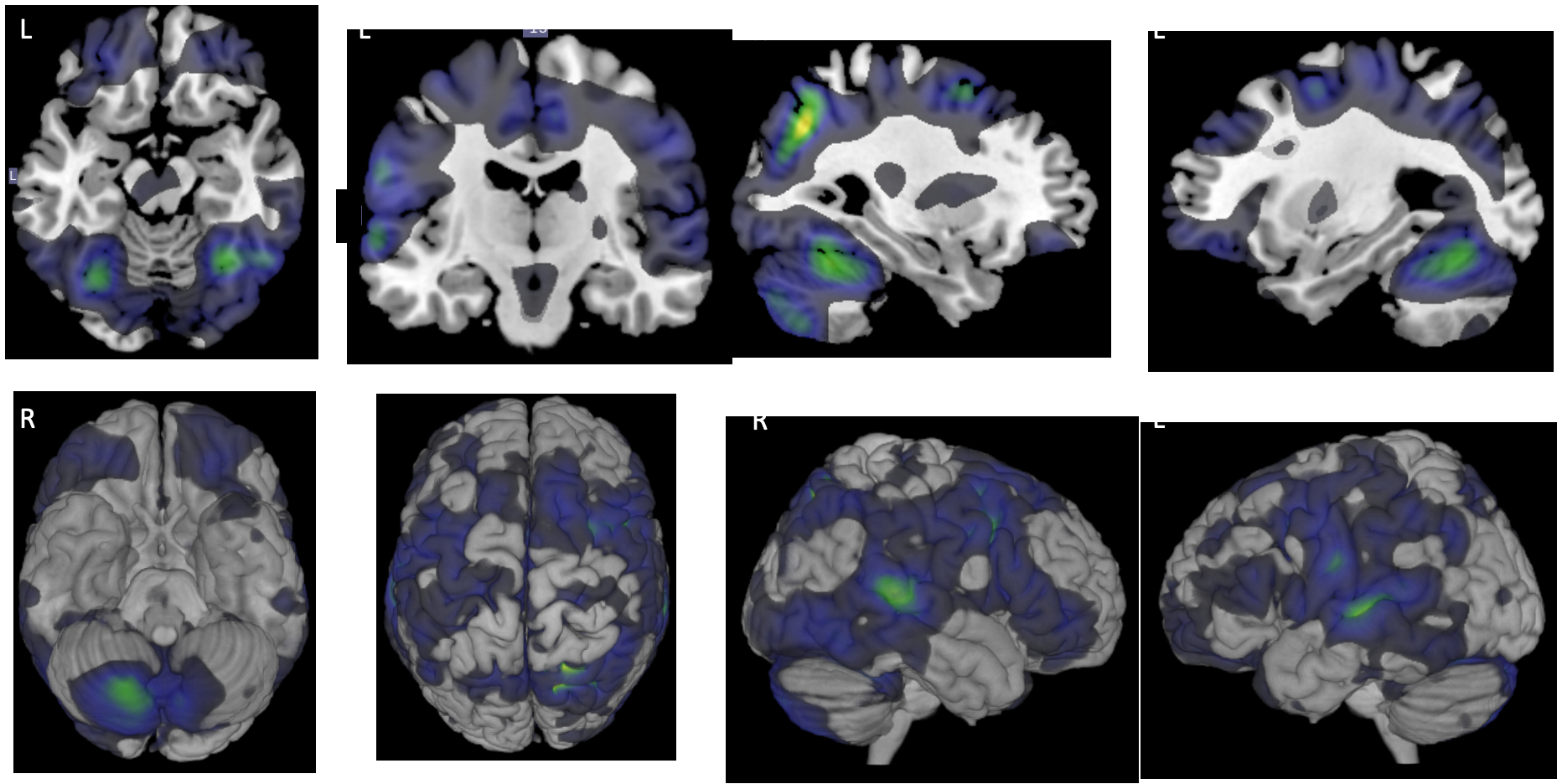 |
| 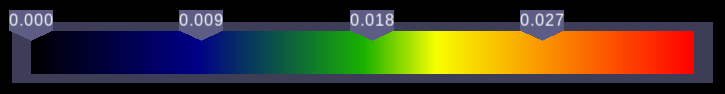 |

Supplementary Fig. 18. VBM p value findings for AD-Memory compared with AD-Visuospatial

| 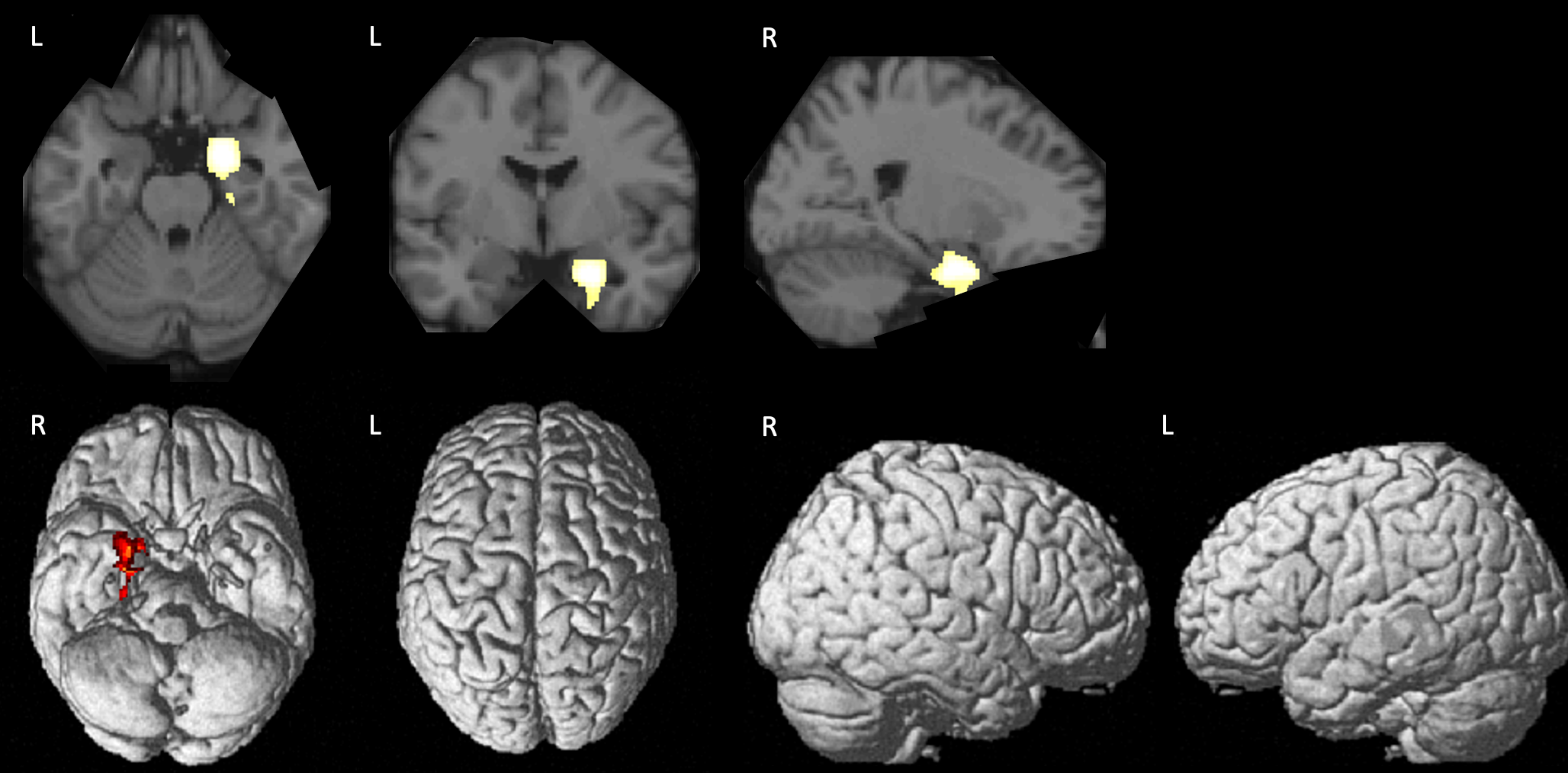 |
| --- |
| 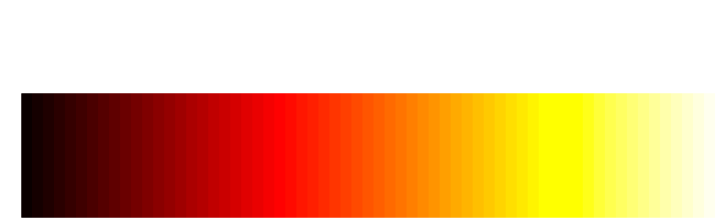 |

Supplementary Fig. 19. VBM findings for AD-Memory compared with AD-Executive*

| A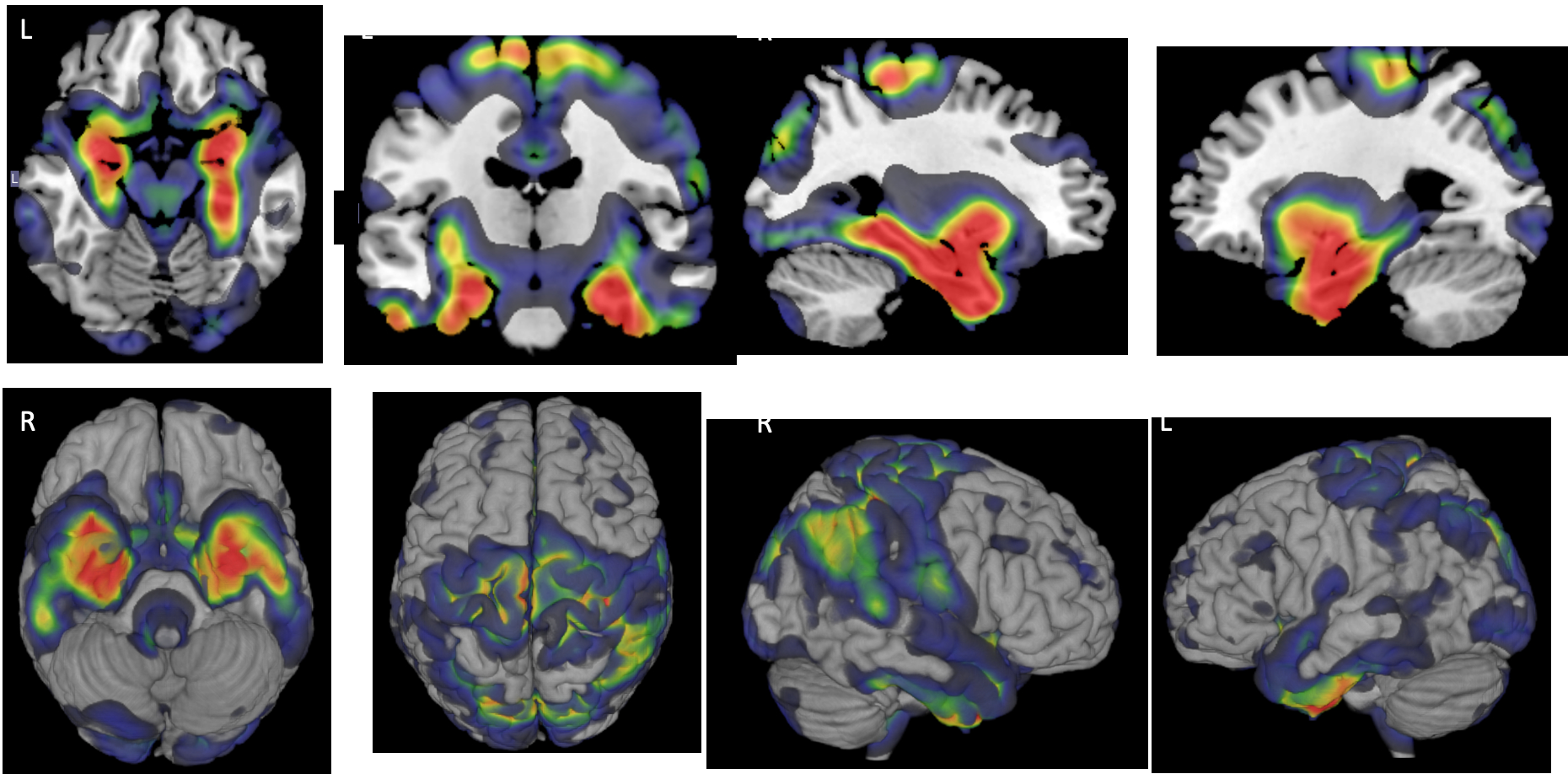 |
| --- |
| B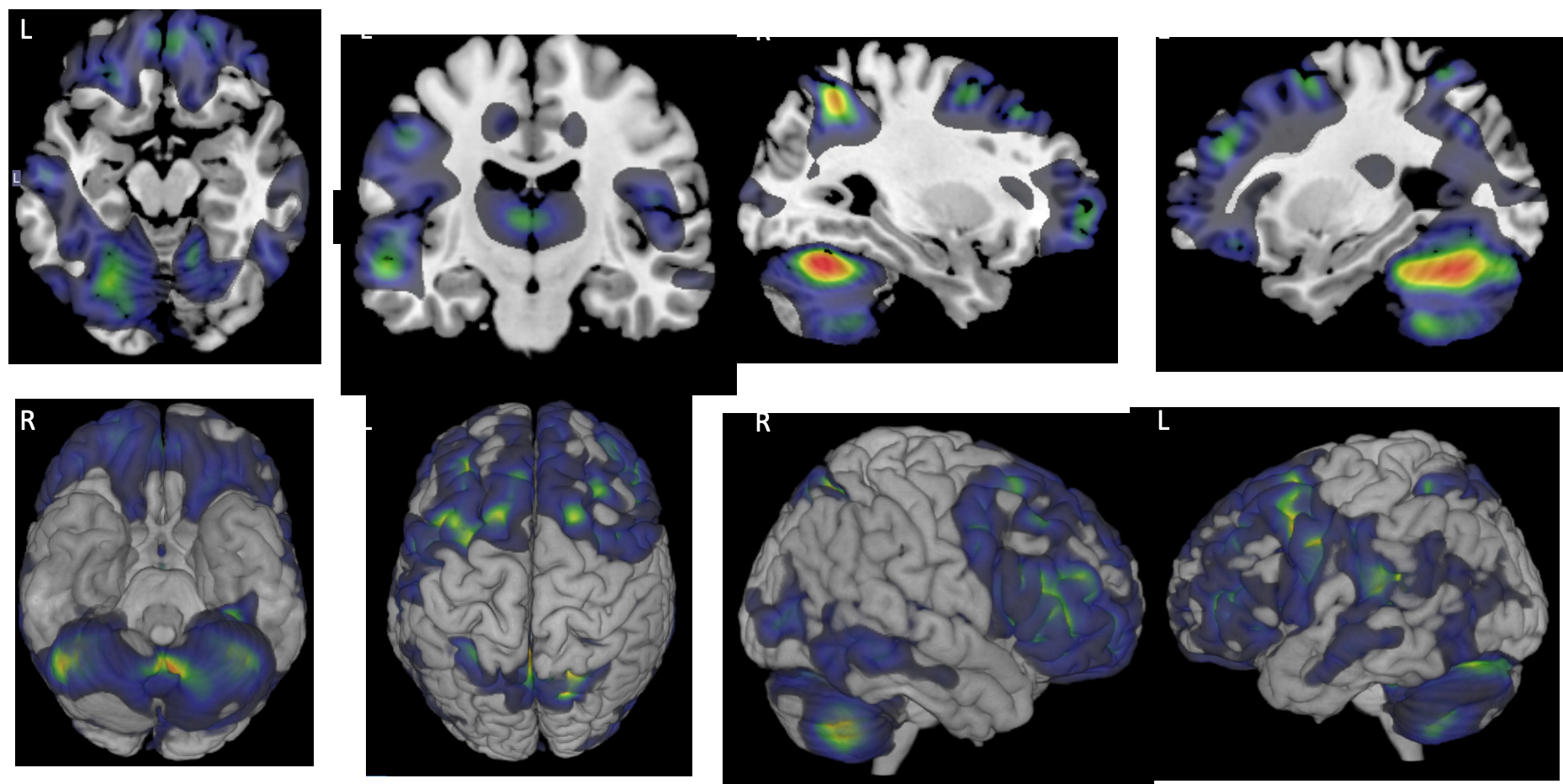 |
| 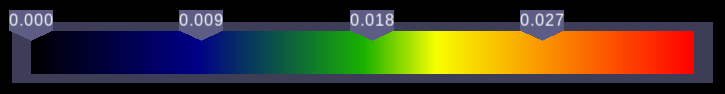 |

* There were no voxels where p values were significantly different in either direction.

Supplementary Fig. 20. VBM findings for AD-Memory compared with AD-Multiple Domains*

| A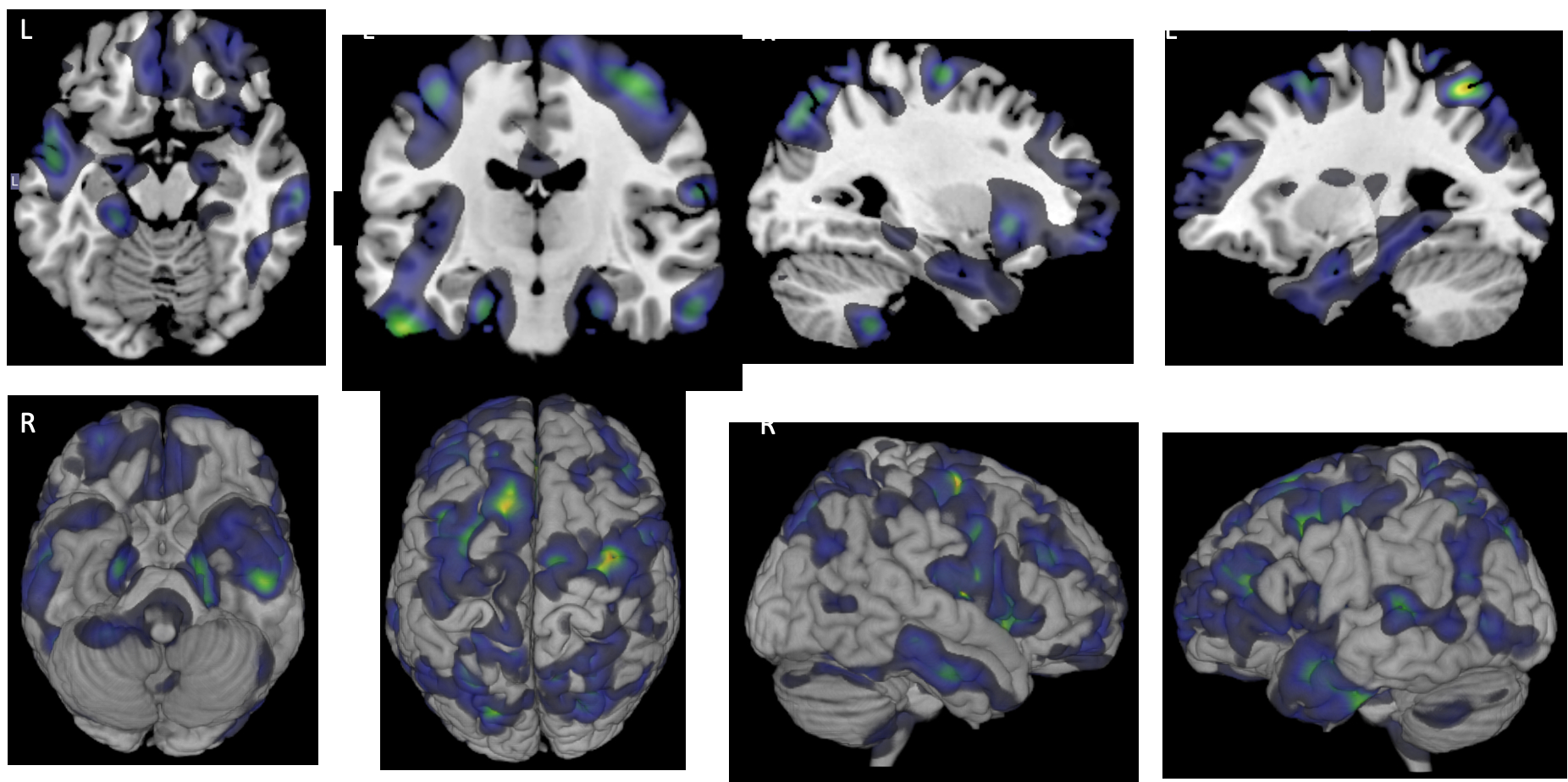 |
| --- |
| B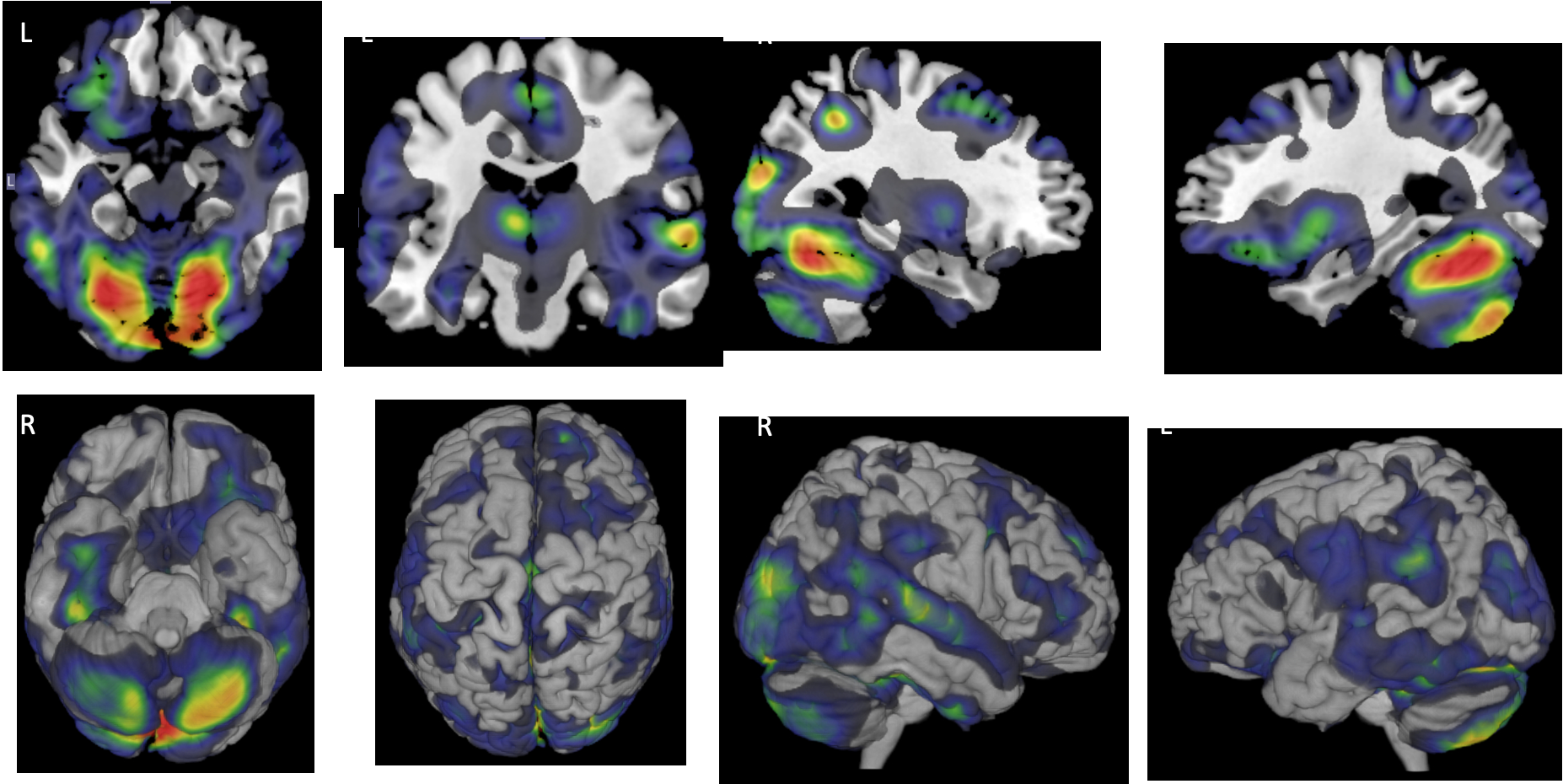 |
| 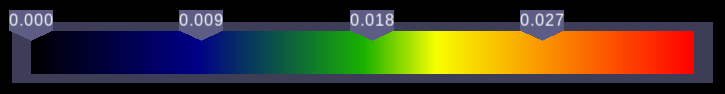 |

* There were no voxels where p values were significantly different in either direction.

Supplementary Fig. 21. VBM findings for AD-No Domains compared to cognitively normal elderly controls for ADNI and VUMC-Amsterdam late-onset AD.

We previously published similar analyses using data from a non-overlapping participant panel from the VUMC Amsterdam research cohort [3]. We have re-processed those data, limiting to older adults to facilitate direct comparison. In the Figures below we show comparisons from the present analyses in ADNI to those previously found from VUMC Amsterdam.

| A. ADNI AD-No-Domains vs. cognitively unimpaired controls, first four views  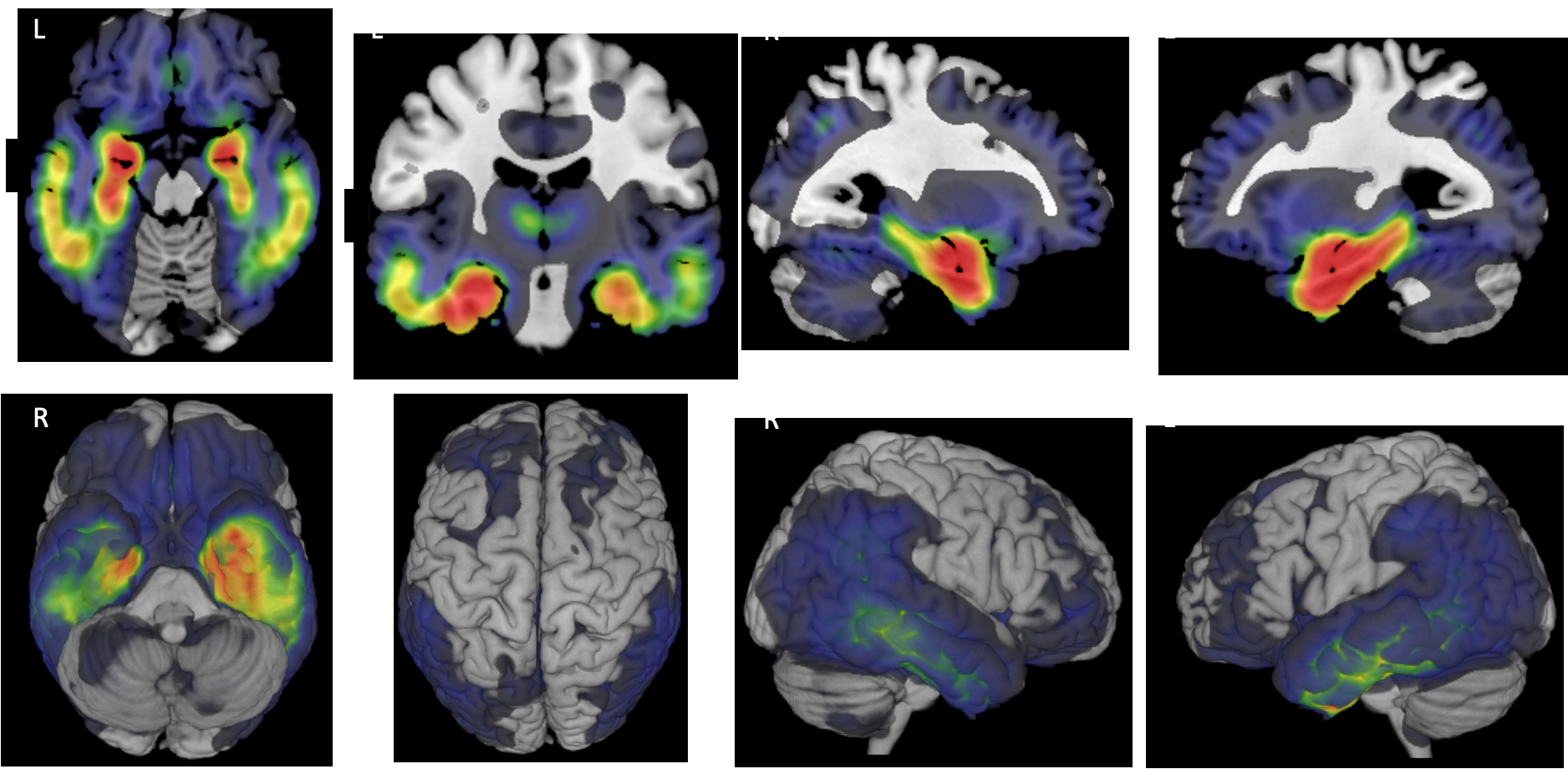 |
| --- |
| B. VUMC Amsterdam AD-No Domains vs. cognitively unimpaired controls, first four views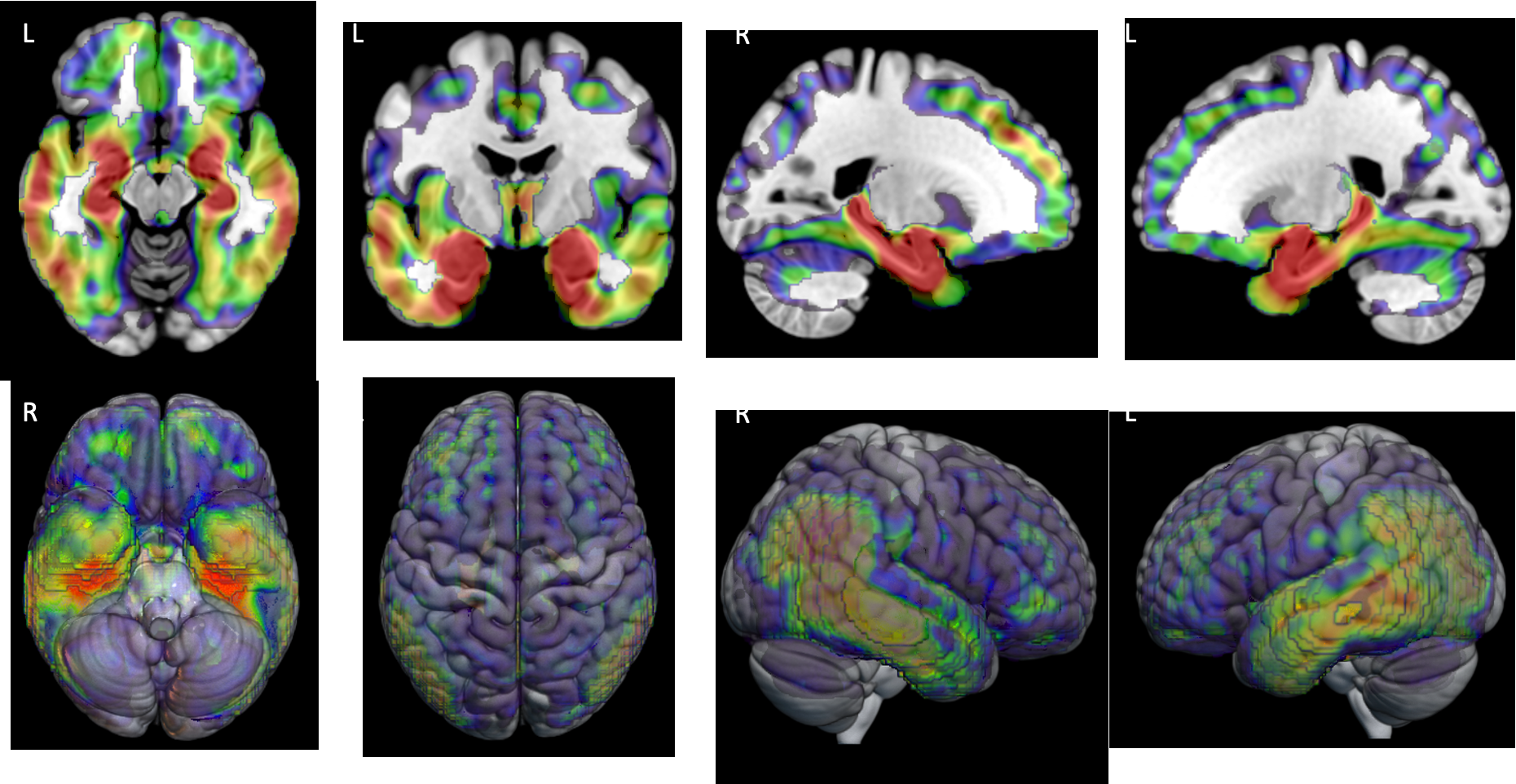 |
| C. ADNI AD-No-Domains vs. cognitively unimpaired controls, second four views 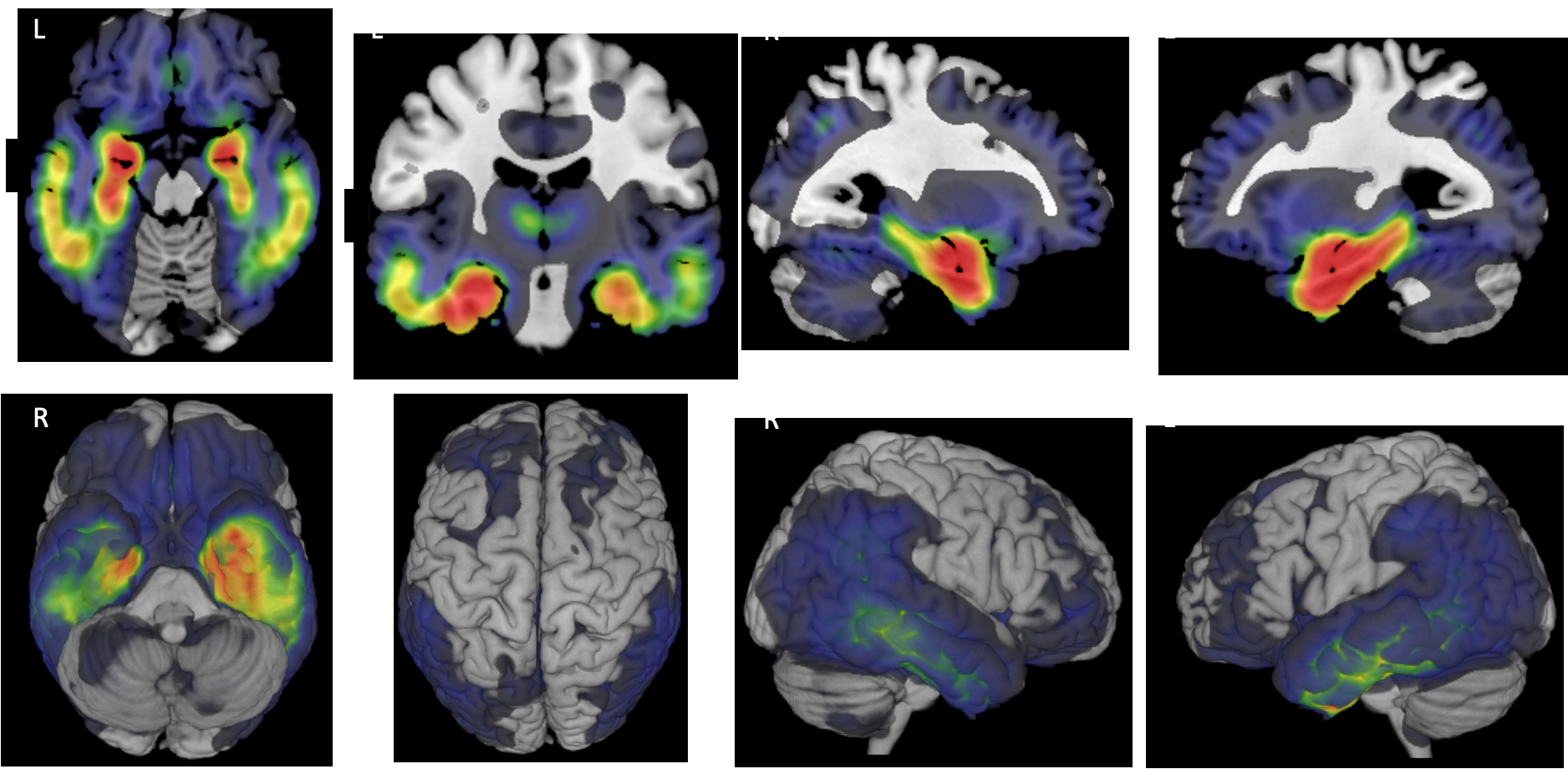 |
| D. VUMC Amsterdam AD-No Domains vs. cognitively unimpaired controls, second four views 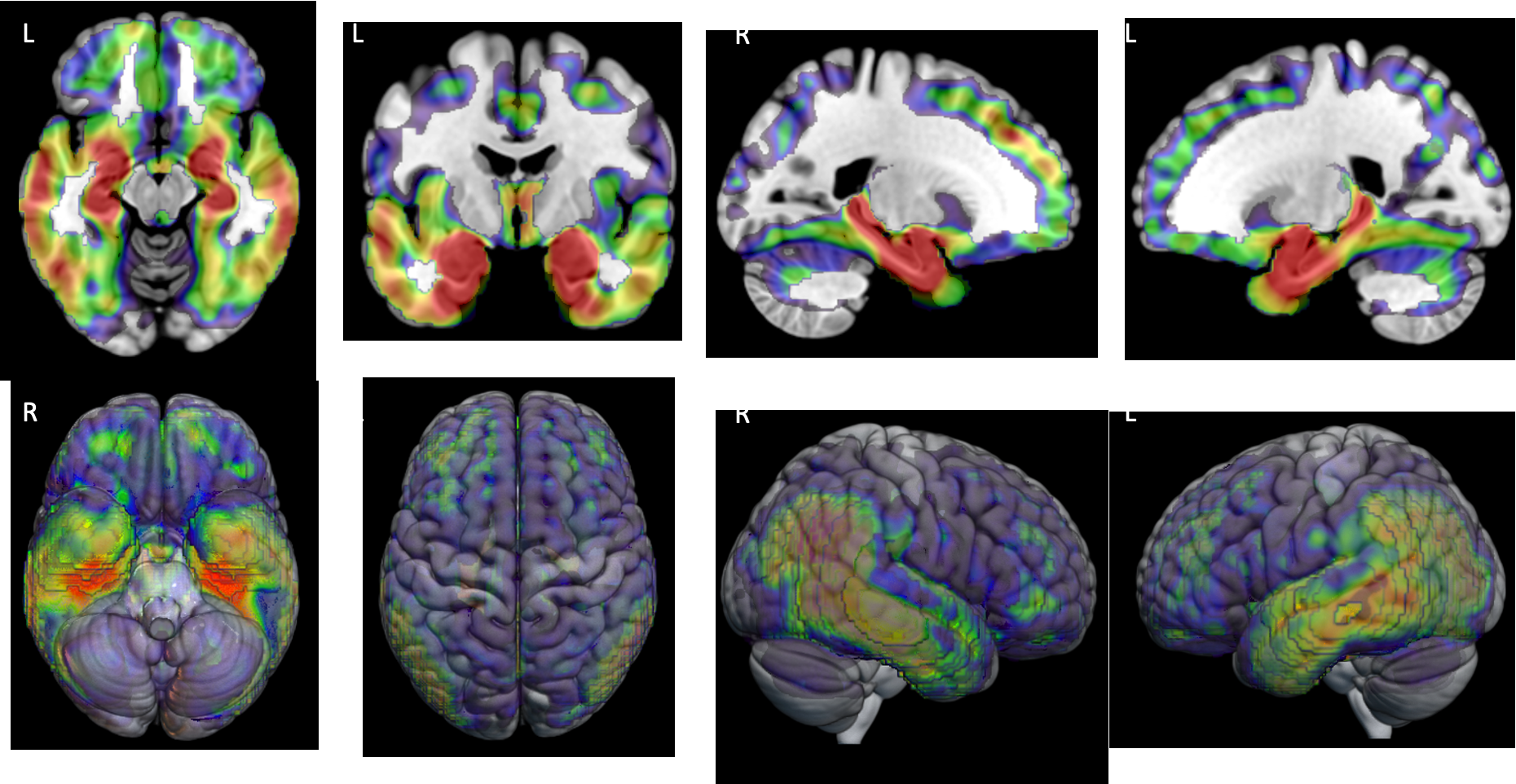 |

Supplementary Fig. 22. VBM findings for AD-Memory compared to cognitively normal elderly controls for ADNI and VUMC-Amsterdam late-onset AD

| A. ADNI AD-Memory vs. cognitively unimpaired controls, first four views 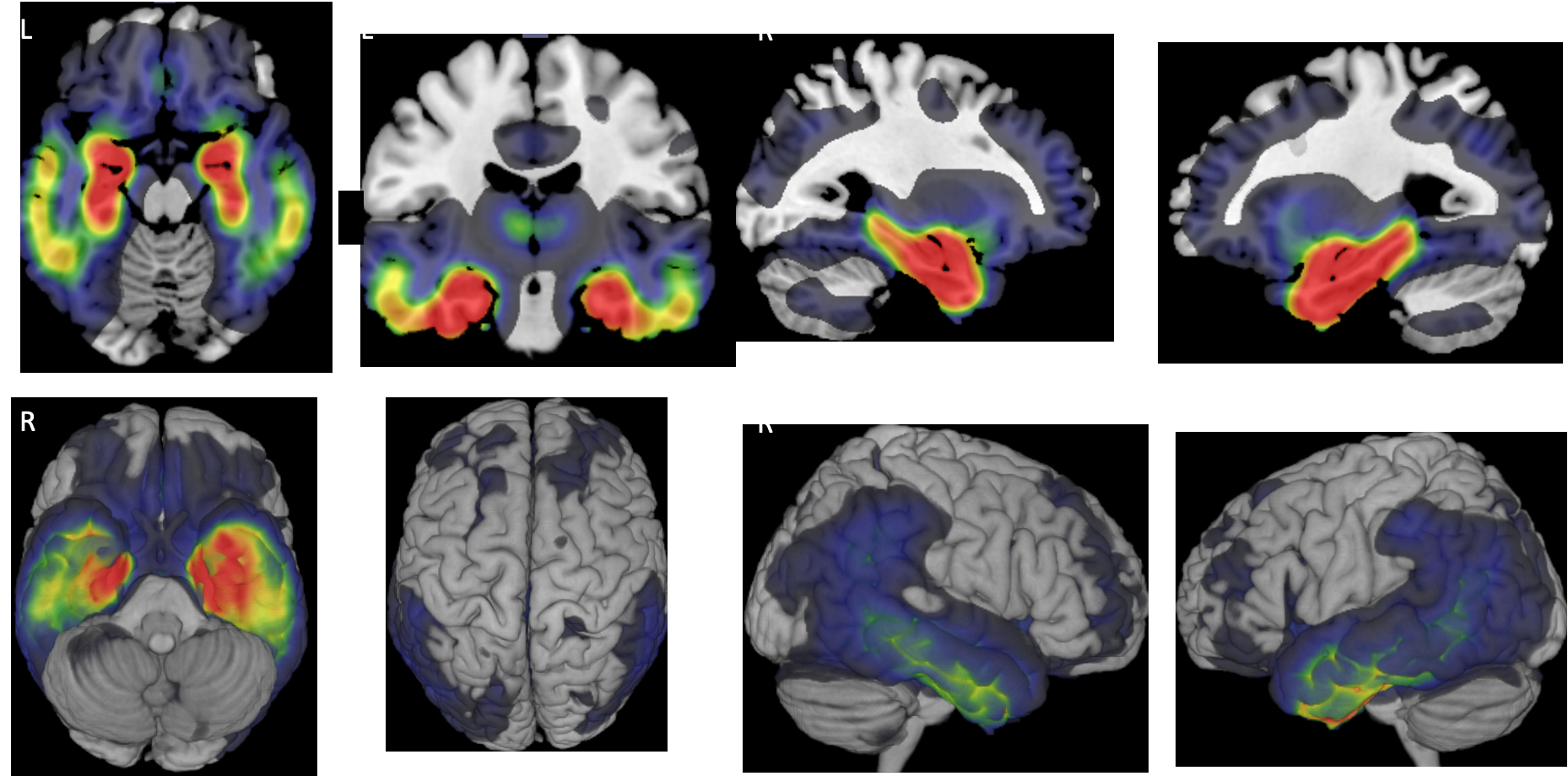 |
| --- |
| B. VUMC Amsterdam AD-Memory vs. cognitively unimpaired controls, first four views  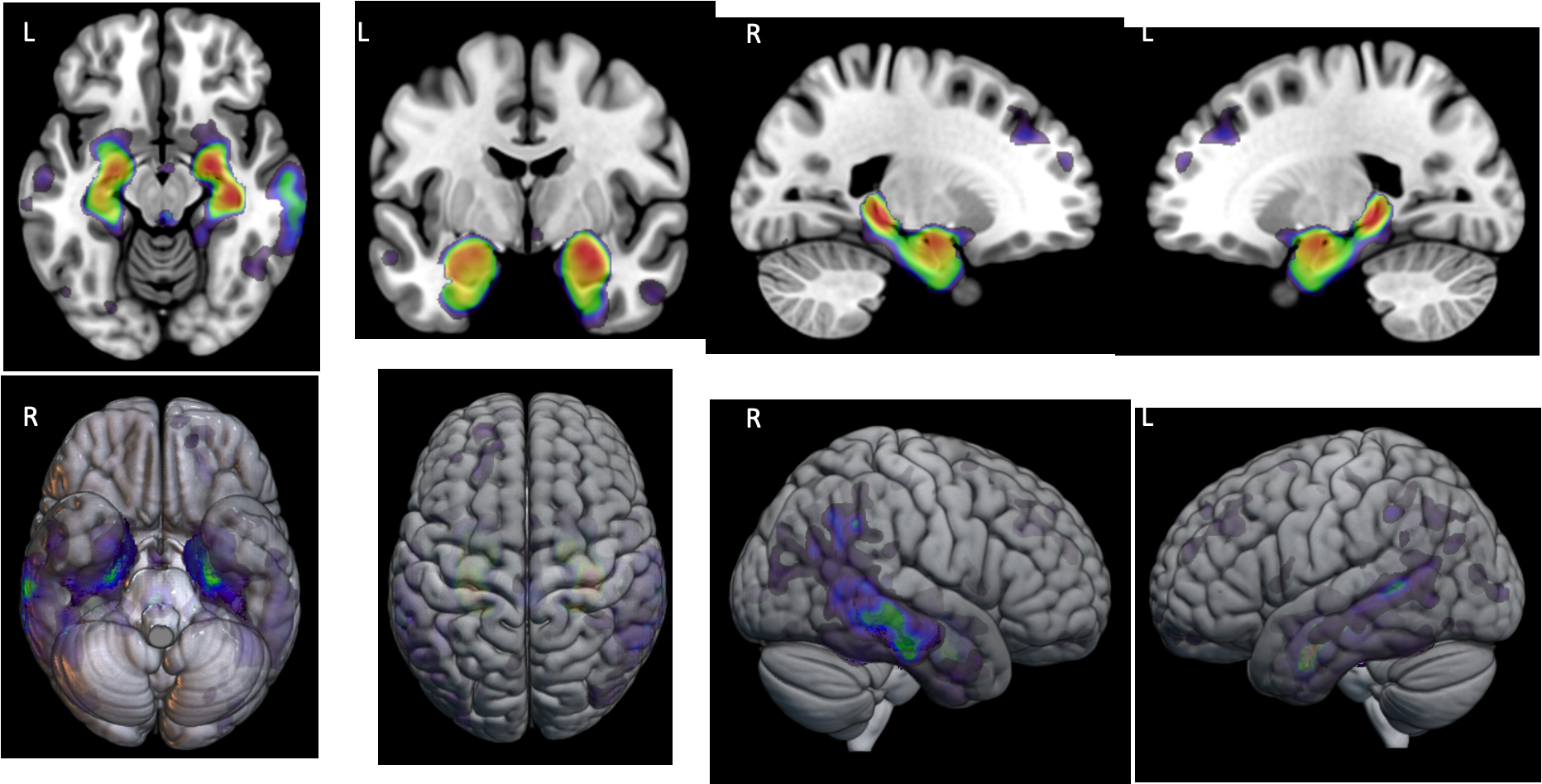 |
| C. ADNI AD-Memory vs. cognitively unimpaired controls, second four views 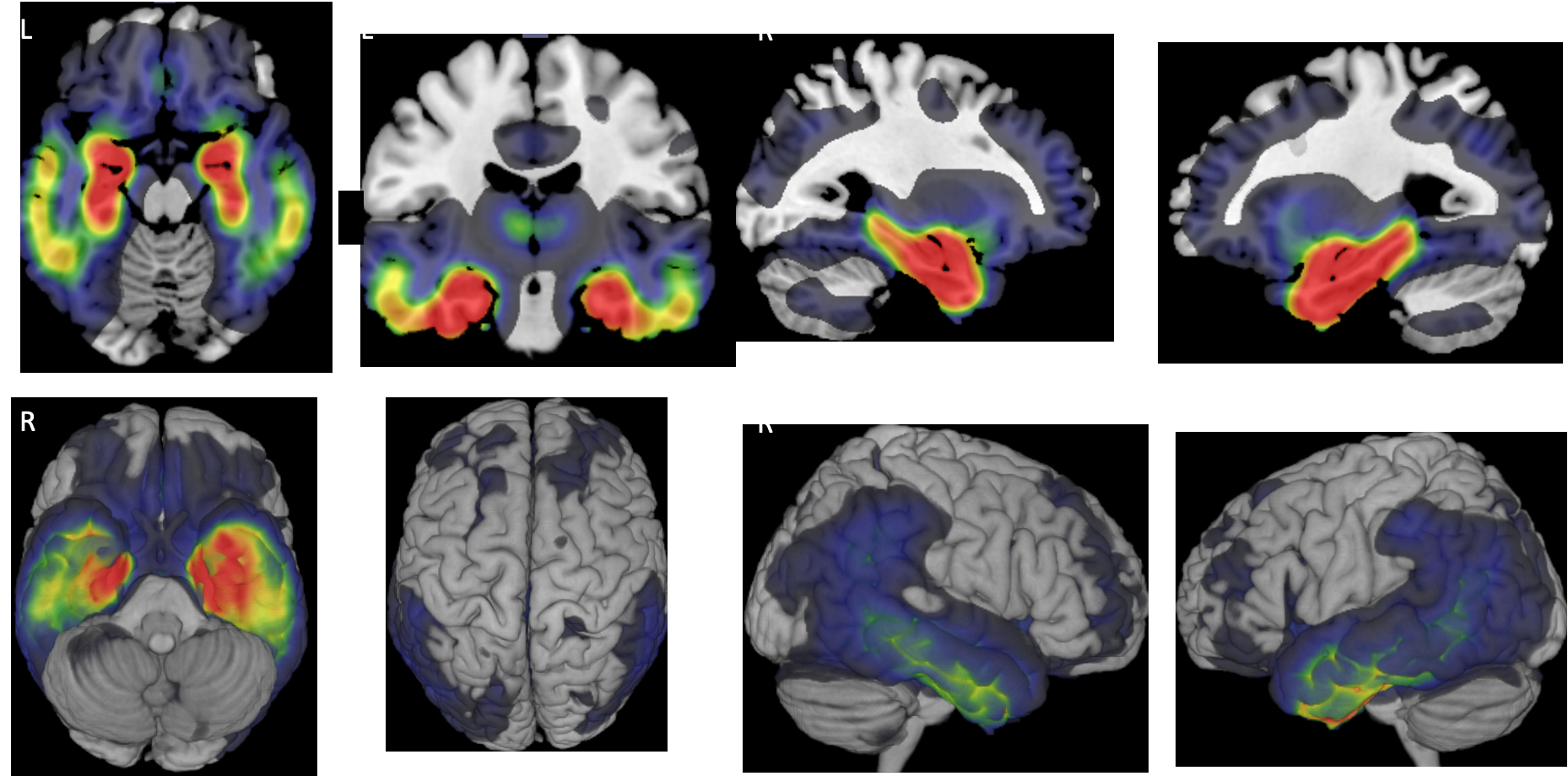 |
| D. VUMC Amsterdam AD-Memory vs. cognitively unimpaired controls, second four views  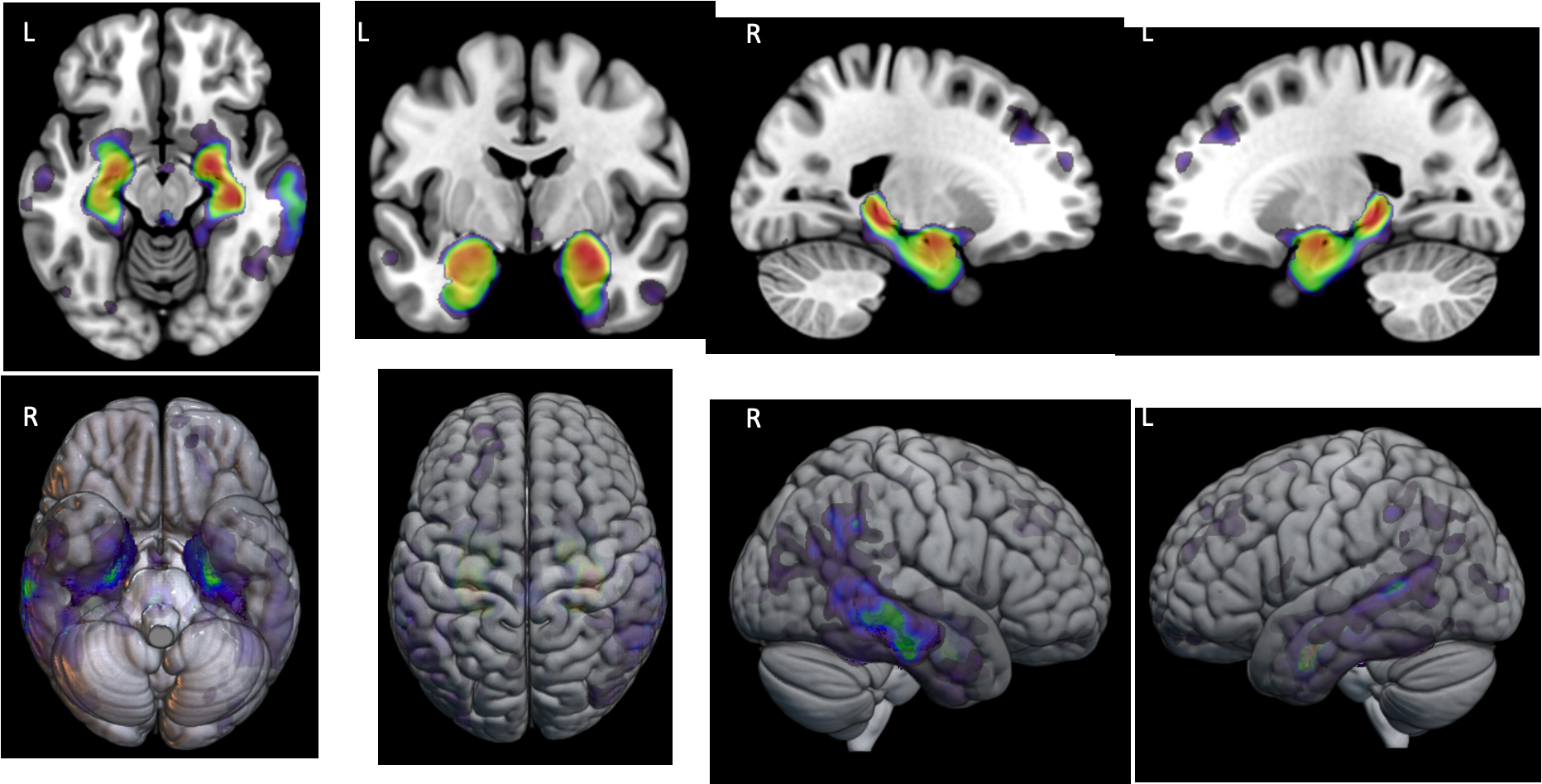 |

Supplementary Fig. 23. VBM findings for AD-Language compared to cognitively normal elderly controls for ADNI and VUMC-Amsterdam late-onset AD

| A. ADNI AD-Language vs. cognitively unimpaired controls, first four views 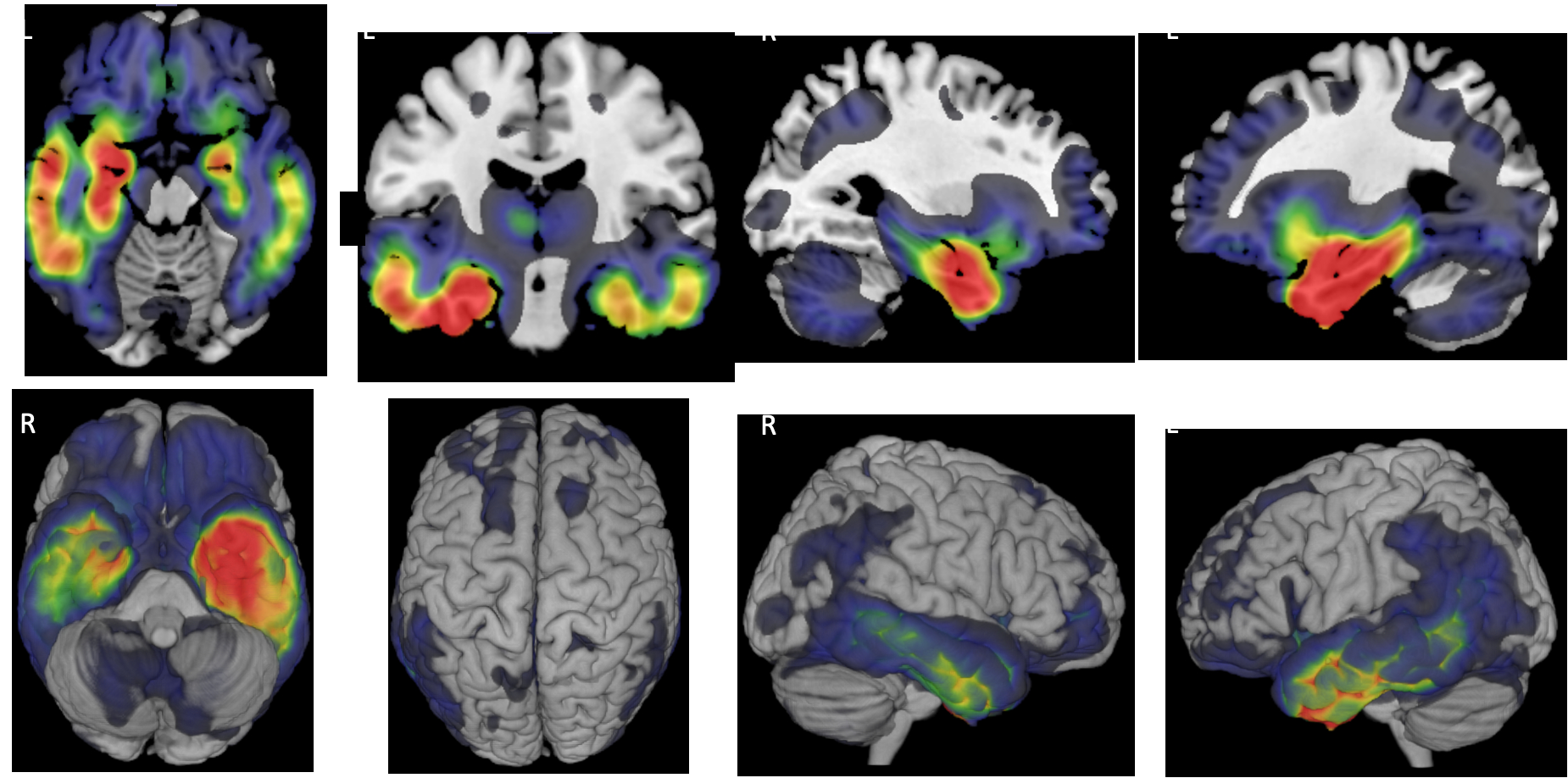 |
| --- |
| B. VUMC Amsterdam AD-Language vs. cognitively unimpaired controls, first four views  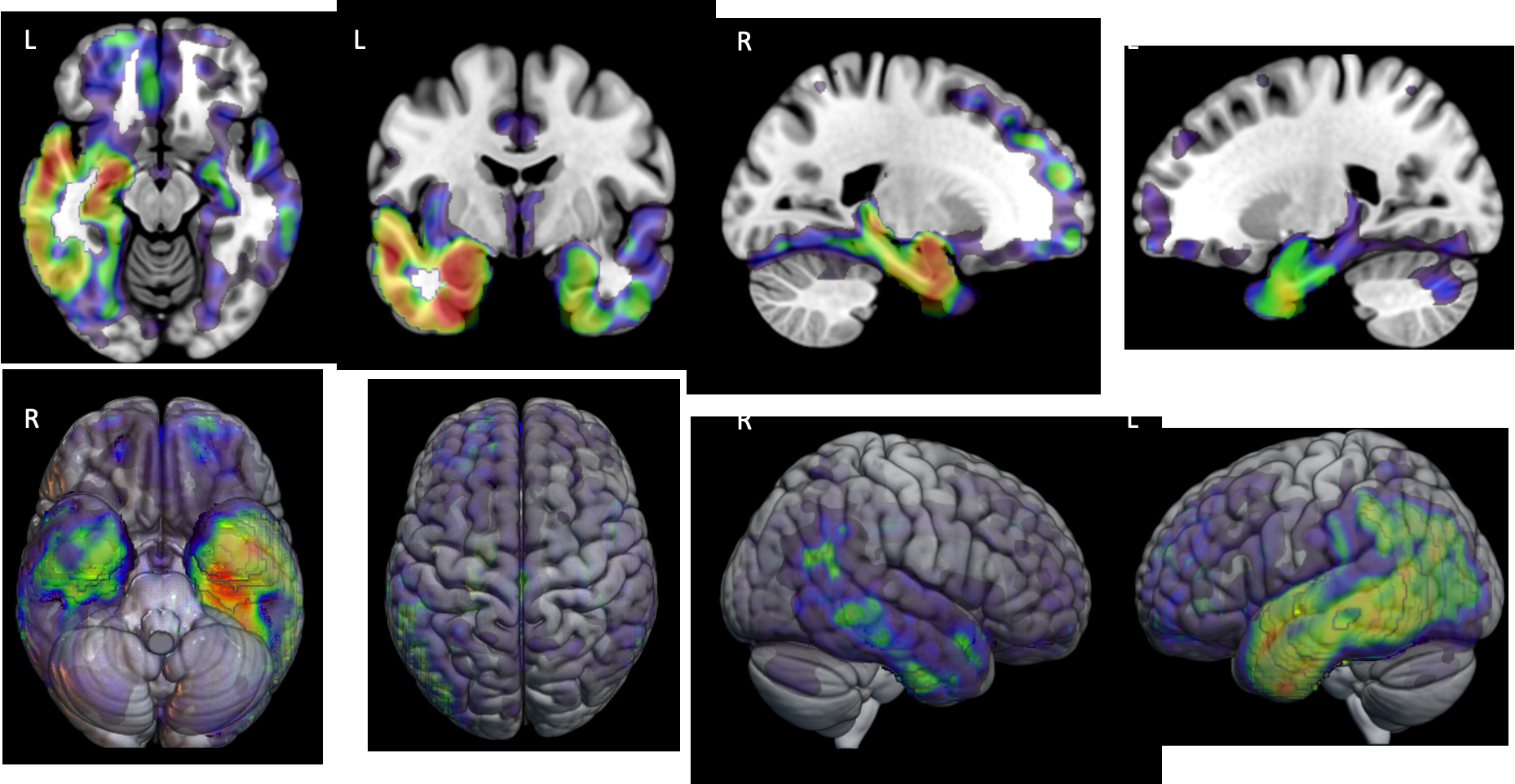 |
| C. ADNI AD-Language vs. cognitively unimpaired controls, second four views 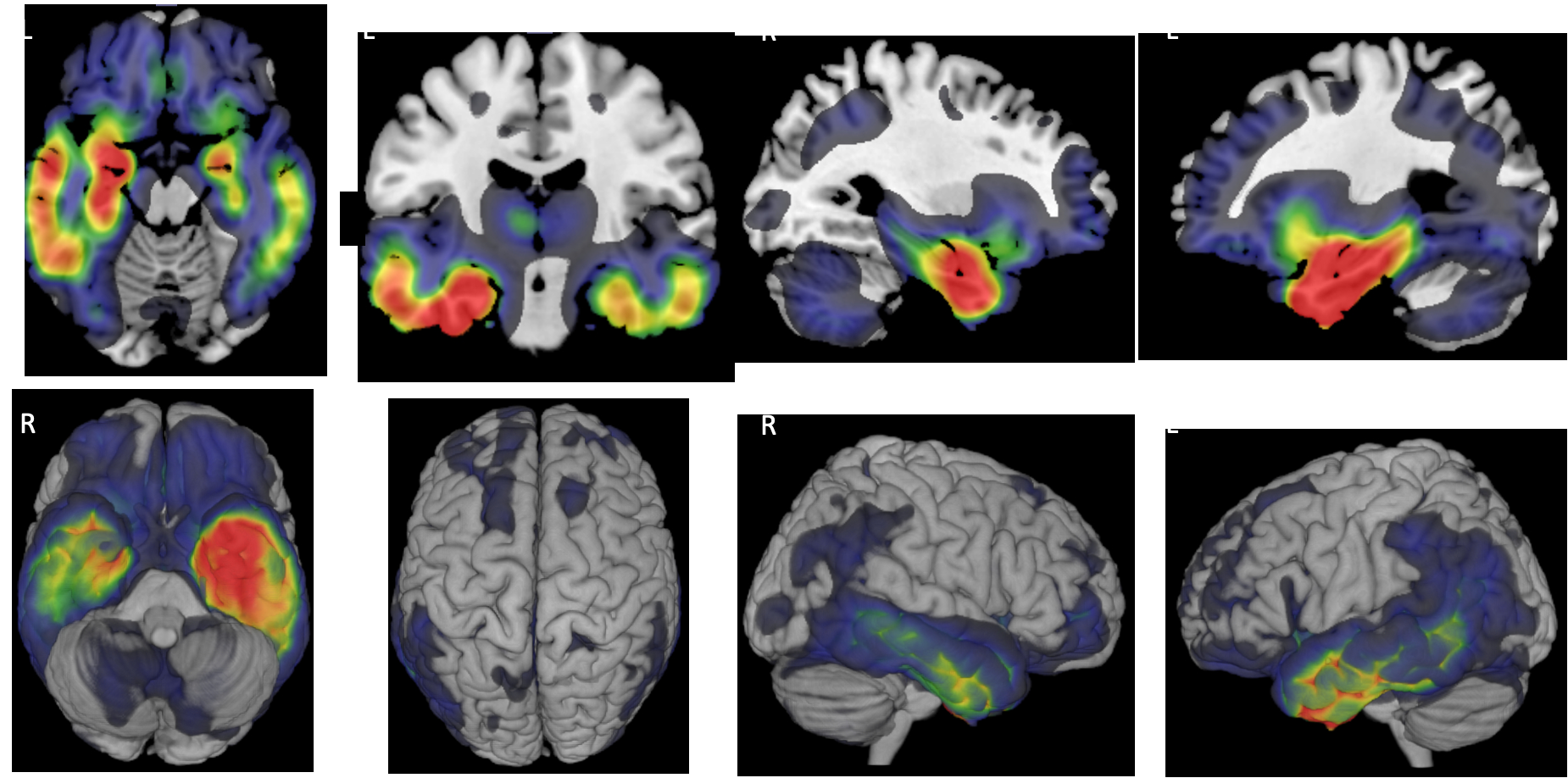 |
| D. VUMC Amsterdam AD-Language vs. cognitively unimpaired controls, second four views  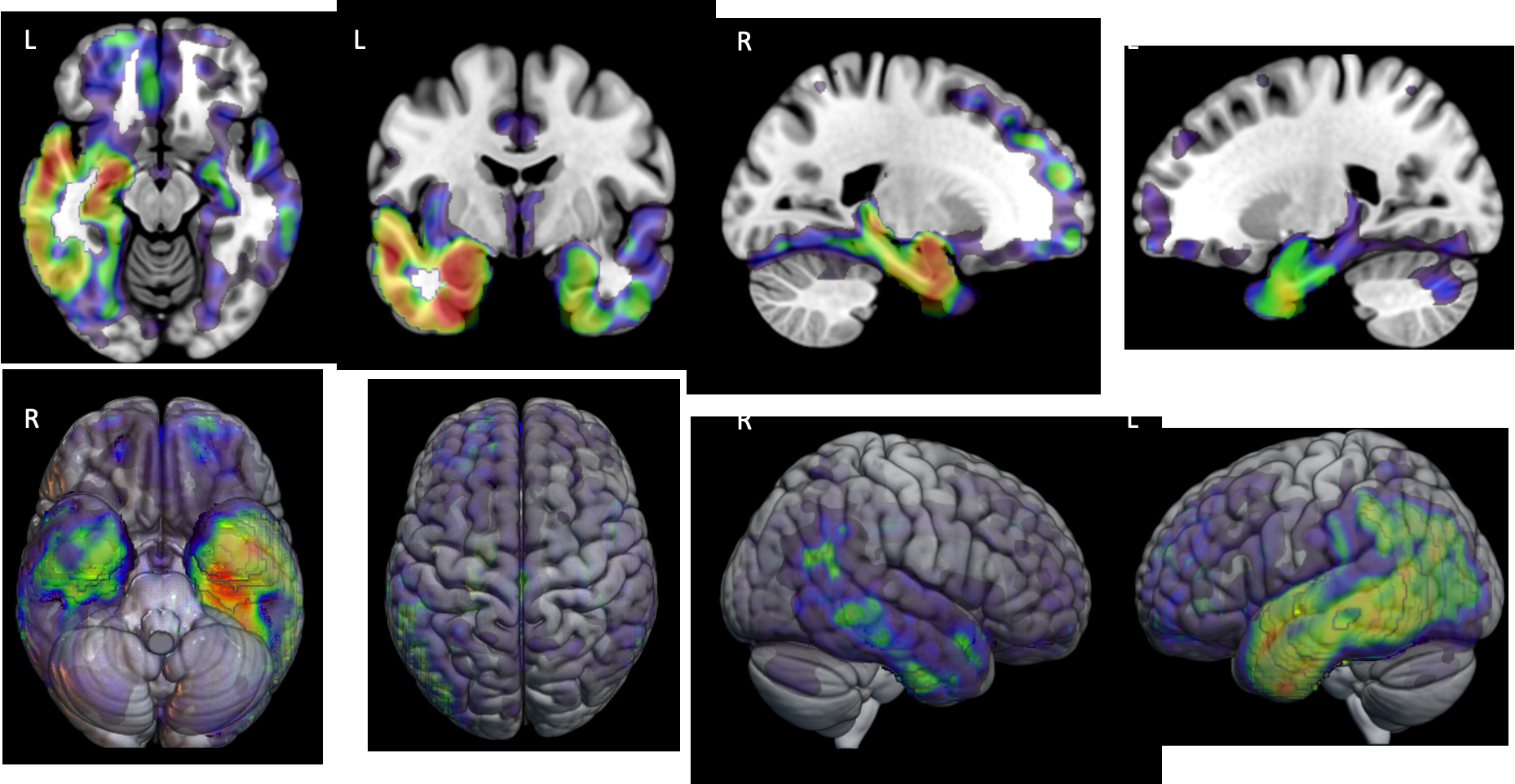 |

Supplementary Fig. 24. VBM findings for AD-Visuospatial compared to cognitively normal elderly controls for ADNI and VUMC-Amsterdam late-onset AD

| A. ADNI AD-Visuospatial vs. cognitively unimpaired controls, first four views 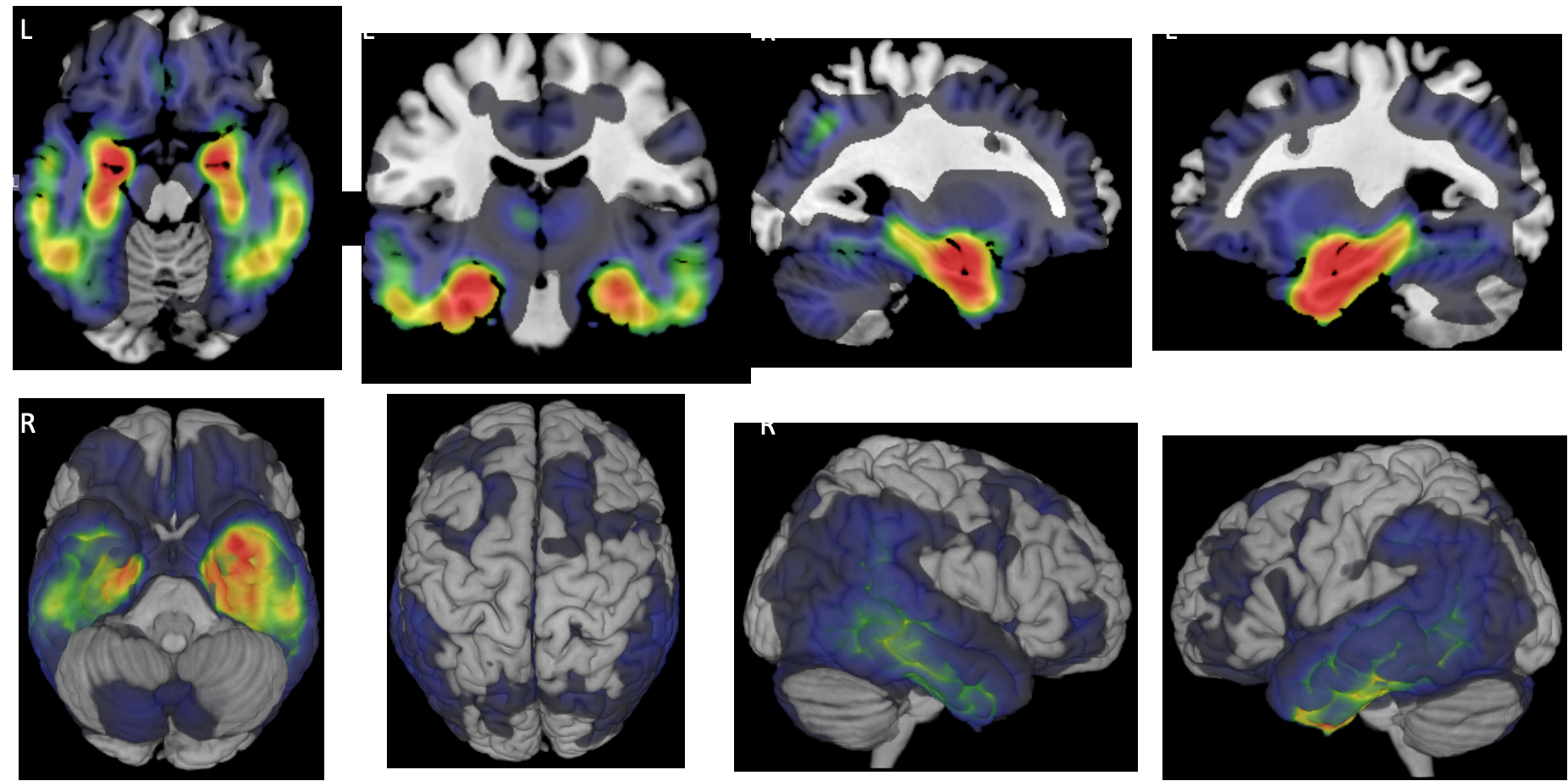 |
| --- |
| B. VUMC Amsterdam AD-Visuospatial vs. cognitively unimpaired controls, first four views  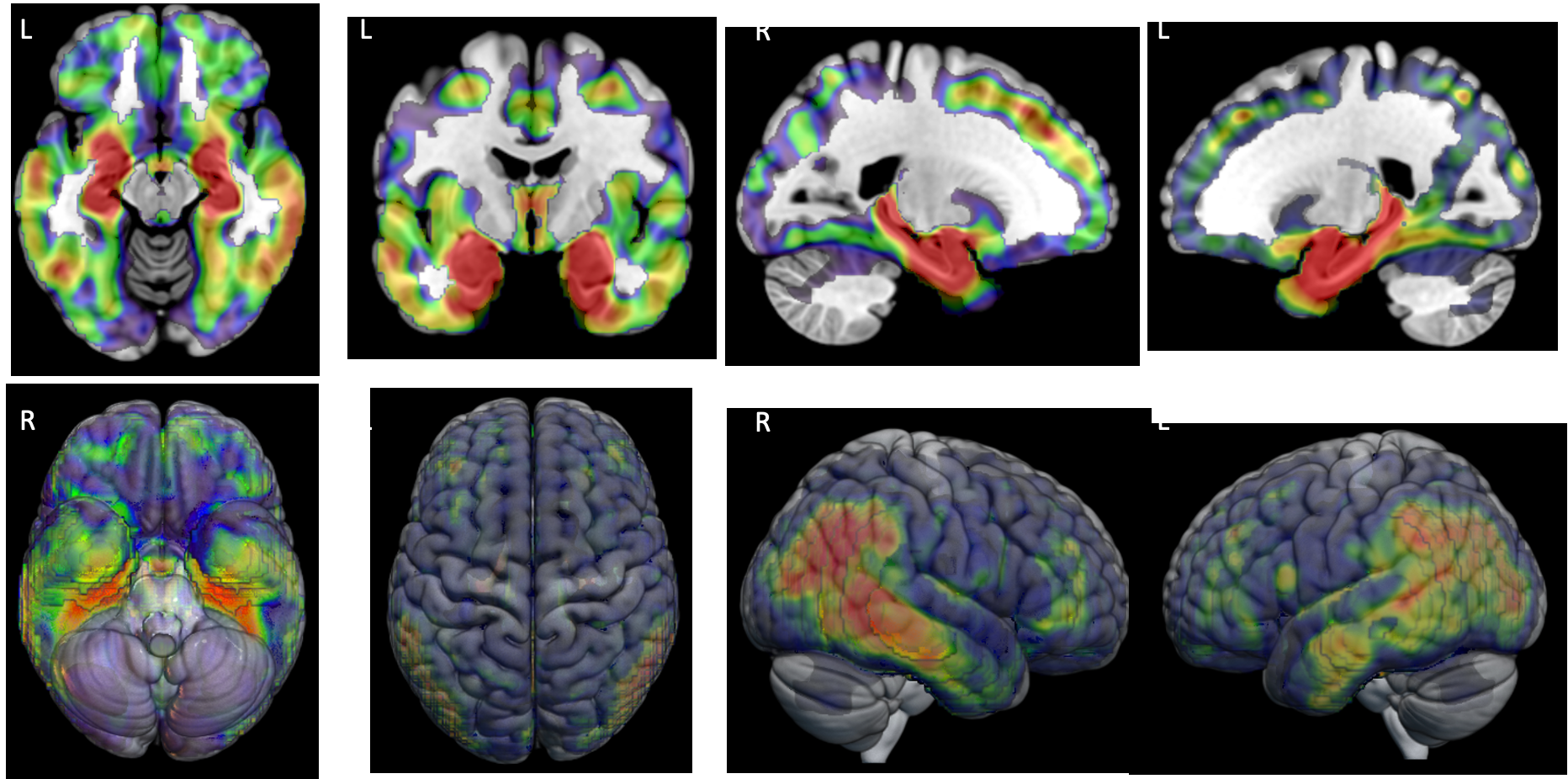 |
| C. ADNI AD-Visuospatial vs. cognitively unimpaired controls, second four views 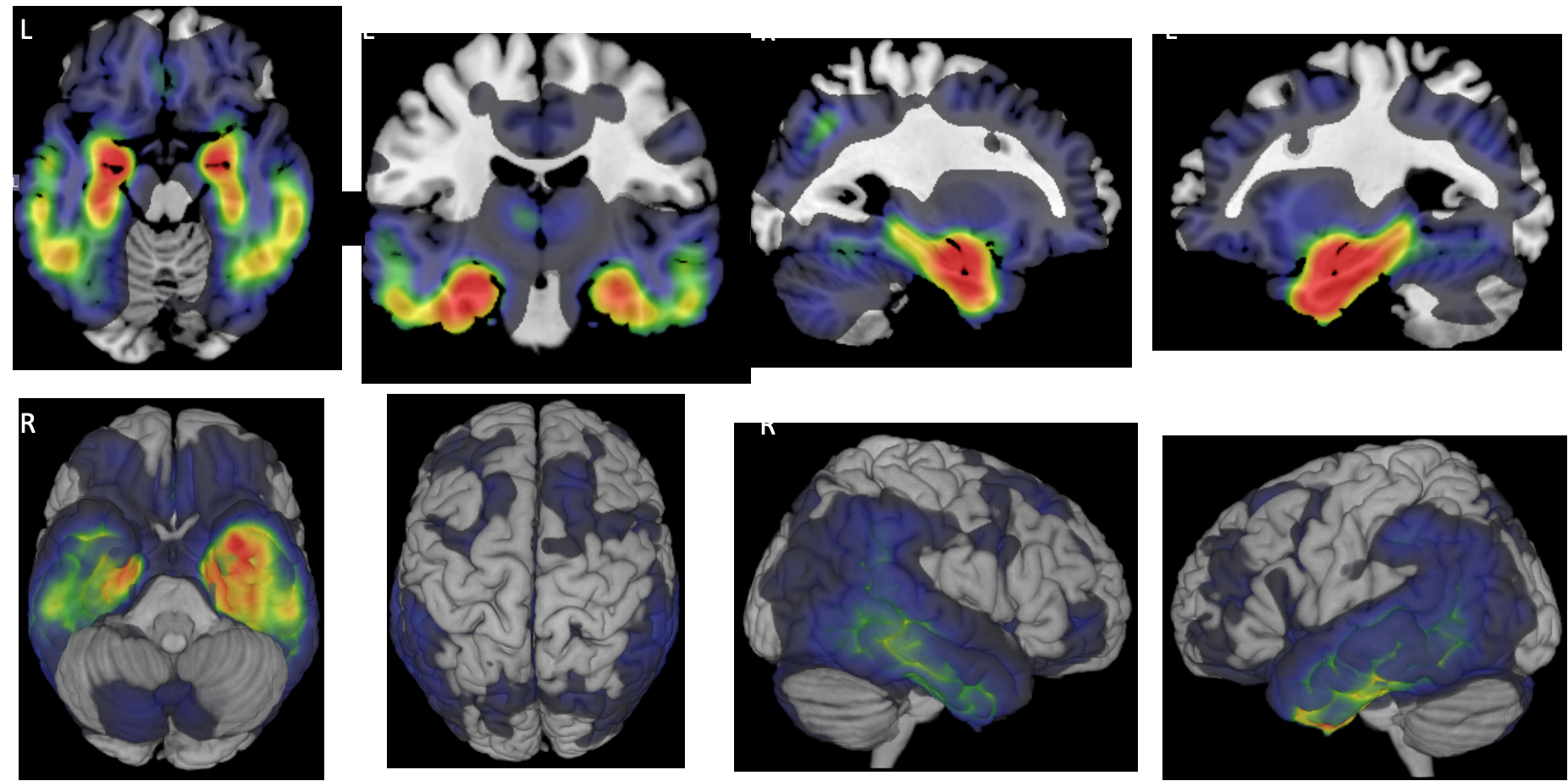 |
| D. VUMC Amsterdam AD-Visuospatial vs. cognitively unimpaired controls, second four views  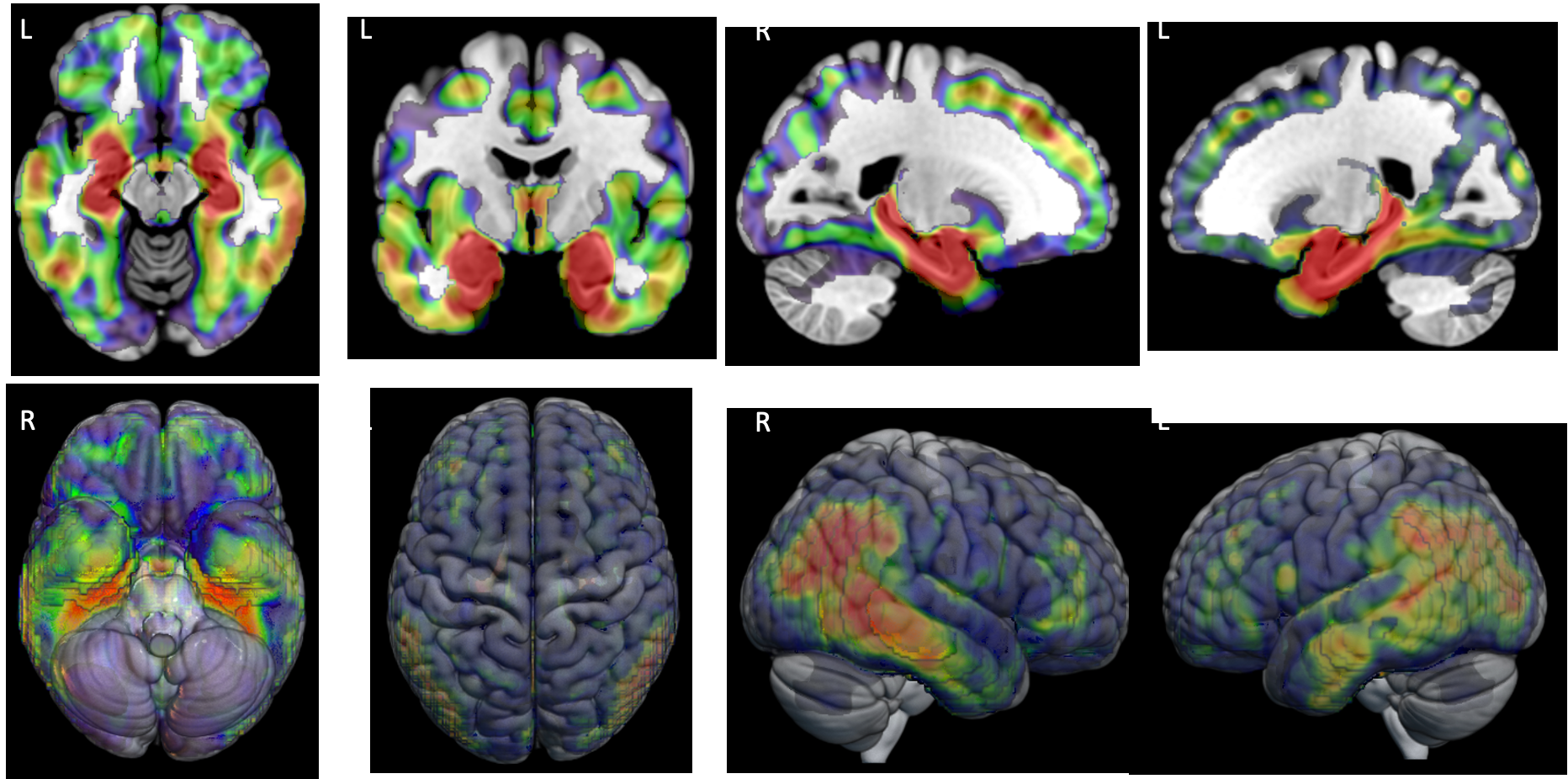 |

Supplementary Fig. 25. VBM findings for AD-Executive compared to cognitively normal elderly controls for ADNI and VUMC-Amsterdam late-onset AD

| A. ADNI AD-Executive vs. cognitively unimpaired controls, first four views 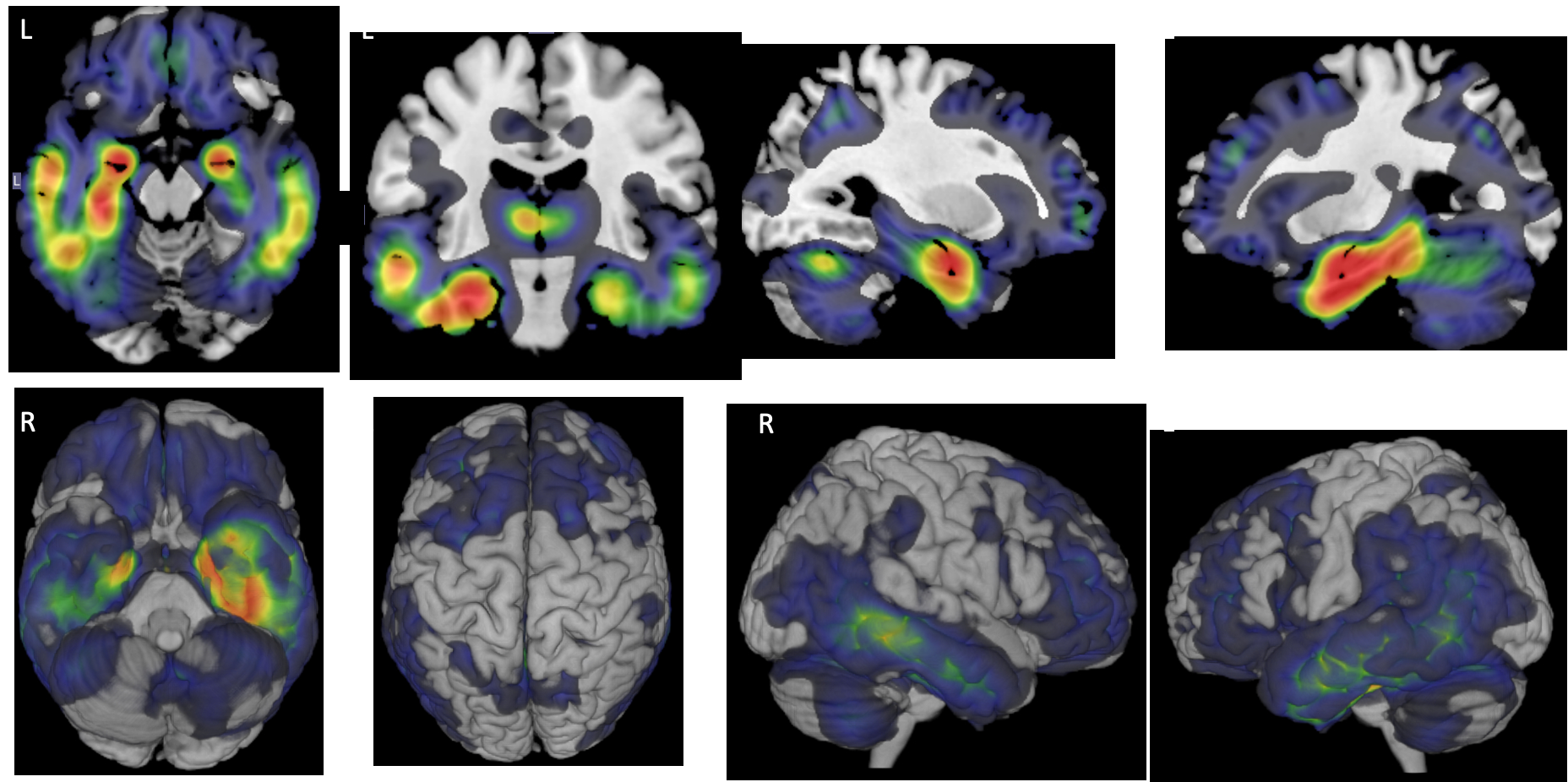 |
| --- |
| B. VUMC Amsterdam AD-Executive vs. cognitively unimpaired controls, first four views  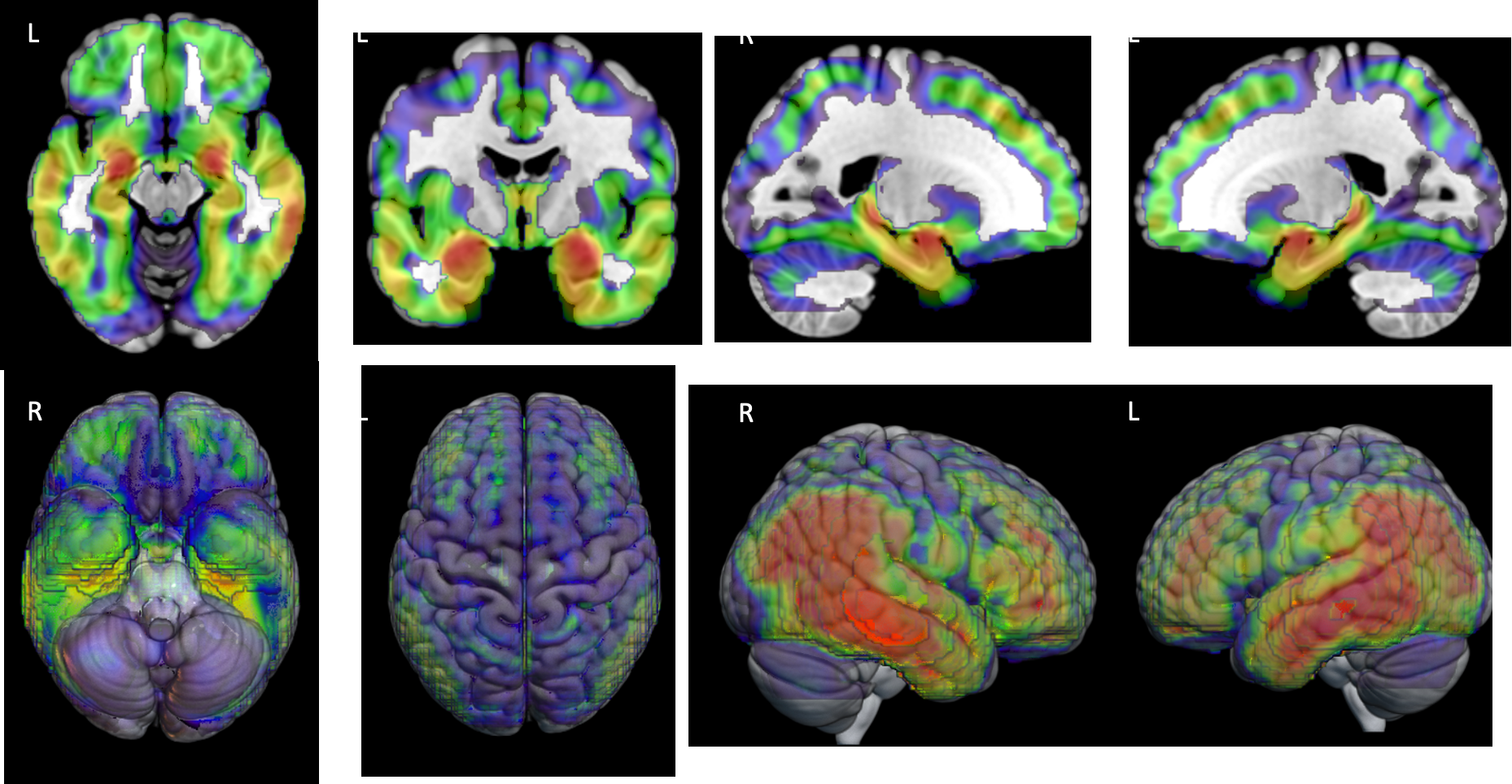 |
| C. ADNI AD-Executive vs. cognitively unimpaired controls, second four views 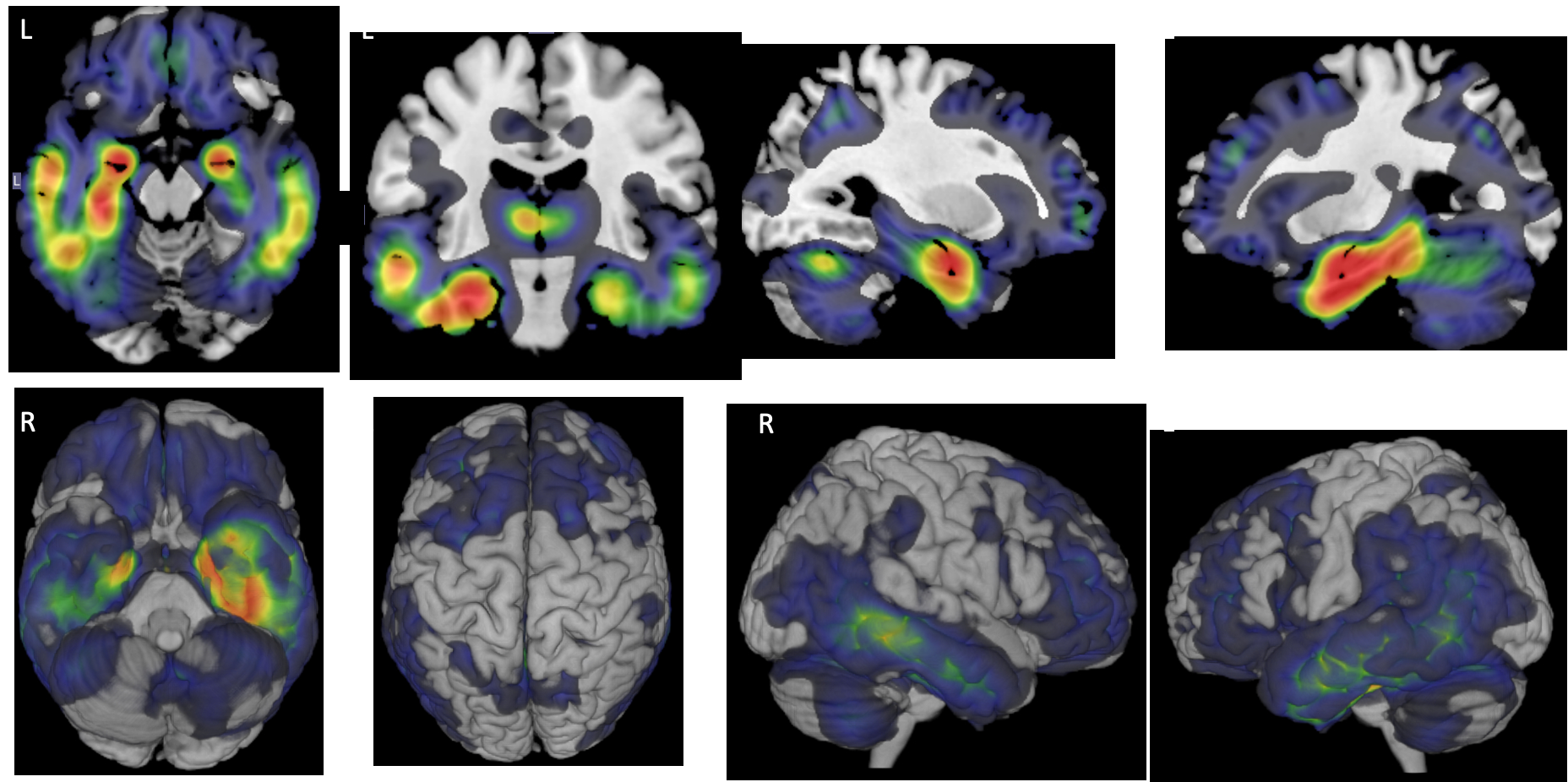 |
| D. VUMC Amsterdam AD-Executive vs. cognitively unimpaired controls, second four views  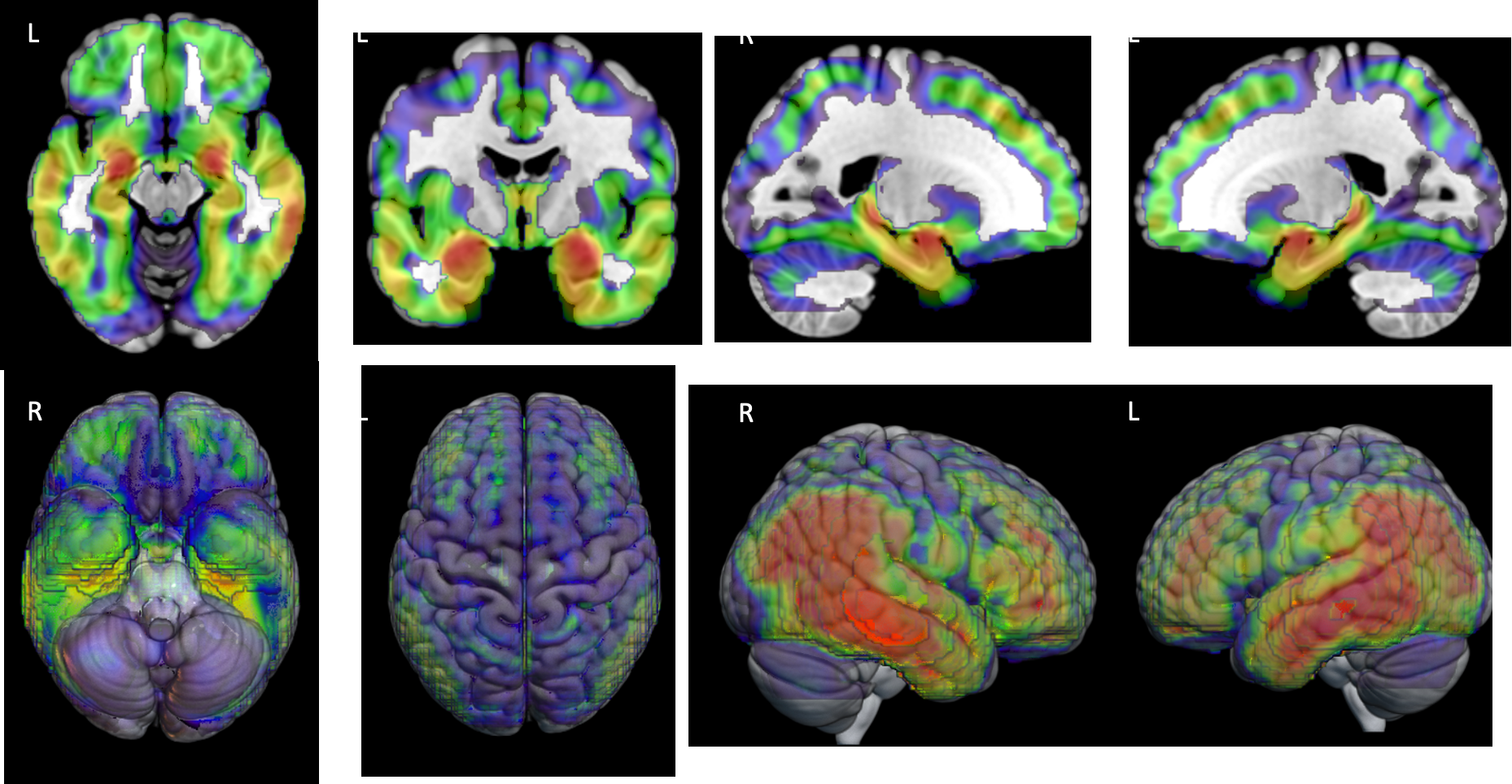 |

Supplementary Table 1: Asymmetry results in z-score units, including left-handed individuals. Negative numbers occur with lower volume on the left than the right.*

|  | **AD-No Domain** | **AD-Memory** | **AD-Language** | **AD-Visuospatial** | **AD-Executive** | **AD -Multiple Domains** |
| --- | --- | --- | --- | --- | --- | --- |
| Overall |  |  |  |  |  |  |
| Global Cortex | -1.07 | -0.97 | **-2.56** | -0.25 | -1.12 | 0.26 |
| Cerebral White Matter | -0.97 | -0.92 | **-2.66** | -0.16 | -1.20 | 0.27 |
| Frontal |  |  |  |  |  |  |
| Frontal Lobe | -1.44 | -1.46 | -1.86 | -0.74 | 0.15 | 0.16 |
| Regional Measures |  |  |  |  |  |  |
| Anterior Cingulate | -1.63 | -0.94 | 0.55 | 0.04 | 0.33 | 1.43 |
| Caudal Anterior Cingulate | -0.97 | -0.83 | 0.99 | 0.21 | 0.76 | 1.35 |
| Caudal Middle Frontal | -1.04 | -0.56 | -1.51 | 0.07 | -0.62 | 0.64 |
| Frontal Pole | 0.47 | -0.20 | 0.06 | -1.27 | 1.07 | 0.34 |
| Lateral Orbitofrontal | -0.24 | 1.40 | -0.86 | -0.64 | -0.31 | -1.75 |
| Medial Orbitofrontal | -0.40 | **-3.02** | -0.66 | -1.65 | 1.02 | -1.80 |
| Middle Frontal | **-1.97** | -1.45 | -1.78 | -1.06 | -0.02 | 0.68 |
| Pars Opercularis | -0.55 | **-2.64** | **-2.08** | -1.34 | 0.00 | -0.51 |
| Pars Orbitalis | -1.09 | -1.23 | -1.02 | 0.03 | -0.41 | **-2.03** |
| Pars Triangularis | -1.36 | **-2.62** | -1.95 | -1.20 | -0.89 | -0.83 |
| Precentral | -0.94 | 0.10 | **-1.99** | -0.34 | -0.76 | -0.38 |
| Rostral Anterior Cingulate | -1.61 | -0.67 | 0.10 | -0.15 | -0.48 | 1.32 |
| Rostral Middle Frontal | -1.52 | -1.27 | -1.79 | -1.20 | 0.43 | 0.25 |
| Superior Frontal | -0.83 | 0.22 | -1.80 | 0.31 | 0.28 | 1.80 |
| Parietal |  |  |  |  |  |  |
| Parietal Lobe | -0.41 | -0.65 | **-2.58** | 0.23 | -1.26 | 0.22 |
| Lateral Parietal | -0.32 | -0.45 | **-2.29** | 0.50 | -1.18 | 0.14 |
| Regional Measures |  |  |  |  |  |  |
| Inferior Parietal | 0.25 | -0.46 | -1.89 | 0.27 | -1.14 | 0.50 |
| Isthmus of Cingulate | -0.42 | -1.25 | **-2.20** | -1.54 | -0.59 | -0.55 |
| Paracentral | -0.24 | 0.02 | -1.30 | 1.19 | 0.61 | 0.49 |
| Posterior Cingulate | -0.72 | -1.76 | -1.20 | 0.75 | 0.06 | 0.13 |
| Postcentral | -1.36 | 0.48 | -1.90 | 0.04 | -0.91 | -0.59 |
| Precuneus | -0.28 | -0.59 | **-2.23** | 0.39 | -0.68 | 0.68 |
| Superior Parietal | -0.46 | -0.79 | -1.31 | 1.18 | -0.44 | 0.10 |
| Supramarginal | -0.86 | 0.12 | **-2.21** | -0.07 | -1.57 | -0.11 |
| Temporal |  |  |  |  |  |  |
| Temporal Lobe | -0.50 | -0.59 | **-2.41** | -0.43 | -1.66 | 0.33 |
| Lateral Temporal Lobe | -0.52 | -0.51 | **-2.21** | -0.47 | -1.32 | 0.40 |
| Medial Temporal Lobe | -0.19 | -0.22 | **-2.51** | -0.33 | **-2.06** | 0.16 |
| Regional Measures |  |  |  |  |  |  |
| Banks Super Temp Sulcus | 0.24 | -0.65 | **-2.61** | 0.18 | -1.21 | 1.20 |
| Entorhinal Cortex | -0.60 | -0.05 | -1.71 | -1.00 | -1.00 | 0.00 |
| Fusiform | 0.33 | 0.10 | **-2.27** | 0.17 | -1.96 | 0.14 |
| Inferior Temporal | -0.57 | -0.24 | -1.83 | -0.25 | -0.89 | -0.10 |
| Middle Temporal | -0.94 | -0.88 | **-2.13** | -0.62 | -1.05 | 0.05 |
| Parahippocampal | **-3.01** | **-2.32** | **-3.72** | -1.22 | **-2.37** | -0.59 |
| Superior Temporal | -0.31 | -0.37 | **-2.12** | -0.49 | **-2.05** | 1.17 |
| Temporal Pole | -0.32 | 0.58 | -1.82 | -1.59 | -1.14 | 0.02 |
| Transverse Temporal Pole | 0.53 | -0.15 | -1.23 | 0.63 | -1.20 | 1.74 |
| Occipital |  |  |  |  |  |  |
| Occipital Lobe | 0.47 | -0.51 | -1.39 | 0.72 | -0.92 | 0.71 |
| Regional Measures |  |  |  |  |  |  |
| Cuneus | 0.44 | 0.44 | -0.56 | 0.59 | 0.17 | 0.57 |
| Lateral Occipital | 0.26 | -0.52 | -0.79 | 1.21 | -0.74 | 0.69 |
| Lingual | -0.25 | -0.22 | **-2.15** | -0.17 | -1.43 | 0.47 |
| Pericalcarine | 0.37 | 0.23 | -0.74 | 0.87 | -0.20 | 0.08 |
| Insula | 1.63 | 0.50 | -1.24 | 0.12 | -0.15 | -0.61 |
| Cingulate | -1.39 | -1.68 | -0.97 | -0.27 | -0.03 | 0.89 |
| Sensorimotor | -1.26 | 0.32 | **-1.98** | 0.08 | -0.85 | -0.36 |
| Cerebellum |  |  |  |  |  |  |
| Cerebellar White Matter | 1.72 | 1.00 | 0.37 | 1.75 | -0.59 | -0.21 |
| Cerebellar Gray Mater | 1.03 | 0.13 | -0.38 | 1.01 | -1.49 | -0.84 |
| Deep structures |  |  |  |  |  |  |
| Accumbens | 0.78 | 1.38 | -0.32 | 0.39 | 1.80 | 0.57 |
| Amygdala | -0.51 | -0.58 | **-2.00** | -0.81 | -0.57 | -0.16 |
| Caudate | -0.22 | -0.60 | -1.95 | 0.75 | 0.15 | 0.91 |
| Hippocampus | -1.94 | -1.59 | **-2.59** | -0.77 | -1.42 | -0.71 |
| Pallidum | 0.73 | 0.93 | -1.61 | -0.05 | 0.31 | -0.48 |
| Putamen | 1.88 | 0.89 | -1.58 | 0.50 | 0.49 | -0.06 |
| Thalamus | -1.82 | **-2.33** | -1.37 | -0.70 | -1.06 | -1.34 |

* **Bold font** indicates observations with p<0.05.

Supplementary Table 2: Asymmetry results in z-score units, limited to right-handed people. Negative numbers occur with lower volume on the left than the right.*

|  | **AD-No Domain** | **AD-Memory** | **AD-Language** | **AD-Visuospatial** | **AD-Executive** | **AD -Multiple Domains** |
| --- | --- | --- | --- | --- | --- | --- |
| Overall |  |  |  |  |  |  |
| Global Cortex | -0.23 | -0.23 | **-2.51** | -0.27 | -0.69 | 0.08 |
| Cerebral White Matter | -0.21 | -0.21 | **-2.54** | -0.23 | -0.71 | 0.07 |
| Frontal |  |  |  |  |  |  |
| Frontal Lobe | -0.21 | -0.18 | -1.13 | -0.28 | 0.25 | -0.17 |
| Regional Measures |  |  |  |  |  |  |
| Anterior Cingulate | -0.17 | -0.08 | 0.05 | 0.05 | 0.01 | 0.39 |
| Caudal Anterior Cingulate | -0.10 | -0.09 | 0.13 | 0.07 | 0.18 | 0.43 |
| Caudal Middle Frontal | -0.17 | -0.;06 | -0.56 | -0.05 | -0.23 | 0.18 |
| Frontal Pole | 0.03 | -0.09 | -0.05 | -0.29 | 0.37 | -0.06 |
| Lateral Orbitofrontal | -0.01 | 0.19 | -0.41 | -0.15 | -0.05 | -0.78 |
| Medial Orbitofrontal | 0.00 | -0.46 | -0.46 | -0.29 | 0.40 | -0.64 |
| Middle Frontal | -0.26 | -0.12 | -0.71 | -0.29 | 0.08 | 0.14 |
| Pars Opercularis | -0.08 | -0.33 | -0.58 | -0.27 | 0.18 | -0.22 |
| Pars Orbitalis | -0.07 | -0.13 | -0.22 | 0.06 | 0.10 | -0.61 |
| Pars Triangularis | -0.16 | -0.32 | -0.52 | -0.24 | -0.10 | -0.45 |
| Precentral | -0.12 | -0.13 | -0.68 | -0.10 | -0.12 | -0.11 |
| Rostral Anterior Cingulate | -0.17 | -0.03 | 0.01 | 0.01 | 0.23 | 0.29 |
| Rostral Middle Frontal | -0.15 | -0.09 | -0.52 | -0.25 | 0.23 | -0.03 |
| Superior Frontal | -0.14 | 0.00 | -0.63 | 0.01 | 0.02 | 0.36 |
| Parietal |  |  |  |  |  |  |
| Parietal Lobe | -0.10 | -0.09 | -1.19 | -0.06 | -0.41 | 0.15 |
| Lateral Parietal | -0.08 | -0.05 | -1.04 | 0.03 | -0.34 | 0.09 |
| Regional Measures |  |  |  |  |  |  |
| Inferior Parietal | 0.02 | -0.06 | -0.70 | -0.04 | -0.26 | 0.21 |
| Isthmus of Cingulate | -0.12 | -0.19 | -0.51 | -0.41 | -0.22 | -0.27 |
| Paracentral | -0.04 | -0.02 | -0.47 | 0.21 | 0.21 | 0.07 |
| Posterior Cingulate | -0.11 | -0.24 | -0.39 | 0.12 | 0.13 | 0.02 |
| Postcentral | -0.16 | 0.06 | -0.67 | 0.01 | -0.25 | -0.26 |
| Precuneus | -0.11 | -0.13 | -0.68 | -0.01 | 0.01 | 0.30 |
| Superior Parietal | -0.11 | -0.03 | -1.31 | -0.14 | -0.95 | 0.57 |
| Supramarginal | -0.09 | 0.07 | -0.65 | -0.05 | -0.48 | -0.03 |
| Temporal |  |  |  |  |  |  |
| Temporal Lobe | -0.05 | -0.17 | **-2.35** | -0.27 | -1.11 | 0.16 |
| Lateral Temporal Lobe | -0.03 | -0.12 | **-2.05** | -0.24 | -0.83 | 0.20 |
| Medial Temporal Lobe | -0.01 | -0.10 | -1.67 | -0.19 | -0.89 | 0.01 |
| Regional Measures |  |  |  |  |  |  |
| Banks Super Temp Sulcus | 0.05 | -0.08 | -0.88 | 0.01 | -0.28 | 0.43 |
| Entorhinal Cortex | -0.06 | -0.06 | -0.95 | -0.29 | -0.54 | -0.10 |
| Fusiform | 0.06 | -0.04 | -1.29 | -0.04 | -0.70 | 0.02 |
| Inferior Temporal | -0.02 | -0.07 | -1.33 | -0.13 | -0.36 | -0.18 |
| Middle Temporal | -0.11 | -0.18 | -1.59 | -0.24 | -0.58 | -0.07 |
| Parahippocampal | -0.40 | -0.38 | -1.54 | -0.35 | -1.25 | -0.33 |
| Superior Temporal | -0.02 | -0.03 | -1.31 | -0.14 | -0.95 | 0.57 |
| Temporal Pole | 0.02 | 0.09 | -0.99 | -0.40 | -0.45 | -0.08 |
| Transverse Temporal Pole | 0.03 | -0.03 | -0.47 | 0.09 | -0.18 | 0.47 |
| Occipital |  |  |  |  |  |  |
| Occipital Lobe | 0.02 | -0.10 | -0.71 | 0.11 | -0.32 | 0.46 |
| Regional Measures |  |  |  |  |  |  |
| Cuneus | 0.06 | 0.04 | -0.22 | 0.10 | -0.01 | 0.28 |
| Lateral Occipital | -0.02 | -0.09 | -0.50 | 0.19 | -0.23 | 0.31 |
| Lingual | -0.02 | 0.00 | -0.61 | -0.04 | -0.53 | 0.33 |
| Pericalcarine | 0.03 | 0.02 | -0.24 | 0.17 | -0.05 | 0.08 |
| Insula | -0.02 | -0.07 | -1.33 | -0.13 | -0.36 | -0.18 |
| Cingulate | -0.18 | -0.20 | -0.30 | -0.07 | -0.02 | 0.25 |
| Sensorimotor | -0.16 | 0.04 | -0.80 | -0.01 | -0.15 | -0.16 |
| Cerebellum |  |  |  |  |  |  |
| Cerebellar White Matter | 0.16 | 0.09 | 0.10 | 0.30 | -0.02 | -0.13 |
| Cerebellar Gray Matter | 0.09 | -0.02 | -0.10 | 0.13 | -0.29 | -0.30 |
| Deep structures |  |  |  |  |  |  |
| Accumbens | 0.15 | 0.16 | -0.18 | 0.06 | 0.58 | 0.16 |
| Amygdala | -0.07 | -0.15 | -0.99 | -0.22 | -0.41 | -0.16 |
| Caudate | 0.00 | -0.30 | -1.53 | -0.25 | -1.55 | -0.51 |
| Hippocampus | -0.30 | -0.30 | -1.53 | -0.25 | -1.55 | -0.51 |
| Pallidum | 0.13 | 0.09 | -0.51 | -0.08 | 0.41 | -0.14 |
| Putamen | 0.30 | 0.10 | -0.77 | -0.01 | 0.38 | -0.02 |
| Thalamus | -0.24 | -0.28 | -0.62 | -0.11 | -0.24 | -0.61 |

* **Bold font** indicates observations with p<0.05.

Supplementary Table 3. Thresholds between quartiles of overall atrophy defined by the count of voxels with W-scores less than -1.5 for each subgroup*

| **Threshold** | **Cognitively Normal Controls** | **AD-No Domain** | **AD-Memory** | **AD-Visuospatial** | **AD-Language** | **AD-Executive** | **AD-Multiple Domains** |
| --- | --- | --- | --- | --- | --- | --- | --- |
| 25% | 5 | 25 | 20 | 22 | 22 | 34 | 19 |
| 50% | 10 | 48 | 35 | 44 | 55 | 70 | 52 |
| 75% | 21 | 90 | 65 | 78 | 116 | 122 | 78 |

* Voxel counts are rounded to the nearest thousand

Supplementary Table 4. Cognitively-defined and anatomically-defined subgroups as in Risacher et al. [1]

| **Poulakis et al. subgroup** | **AD-No Domain** | **AD-Memory** | **AD-Visuospatial** | **AD-Language** | **AD-Executive** | **AD-Multiple Domains** | **Totals (%)** |
| --- | --- | --- | --- | --- | --- | --- | --- |
| Typical AD | 174 | 130 | 48 | 28 | 13 | 10 | 403 (69%) |
| Limbic Predominant | 27 | 51 | 10 | 6 | 0 | 6 | 100 (17%) |
| Hippocampal Sparing | 42 | 11 | 7 | 8 | 7 | 4 | 79 (14%) |
| Total (%) | 243 (42%) | 192 (33%) | 65 (11%) | 42 (7%) | 20 (3%) | 20 (3%) | 582 (100%) |

We compared these categorizations using a chi squared test; Chi squared with 10 degrees of freedom was 42.19 and a p value of 6.94 x 10^-6^. Given the 0 cell we considered the data with AD-Executive removed. The chi squared with 8 degrees of freedom was 31.74 and the p value was 1.03 x 10^-4^. Fisher’s exact test for the comparison with ED-Executive removed showed a p = 3.78x10^-5^.

Supplementary Fig. 26. Comparison of cognitively-defined subgroups and anatomically-defined subgroups.*

| 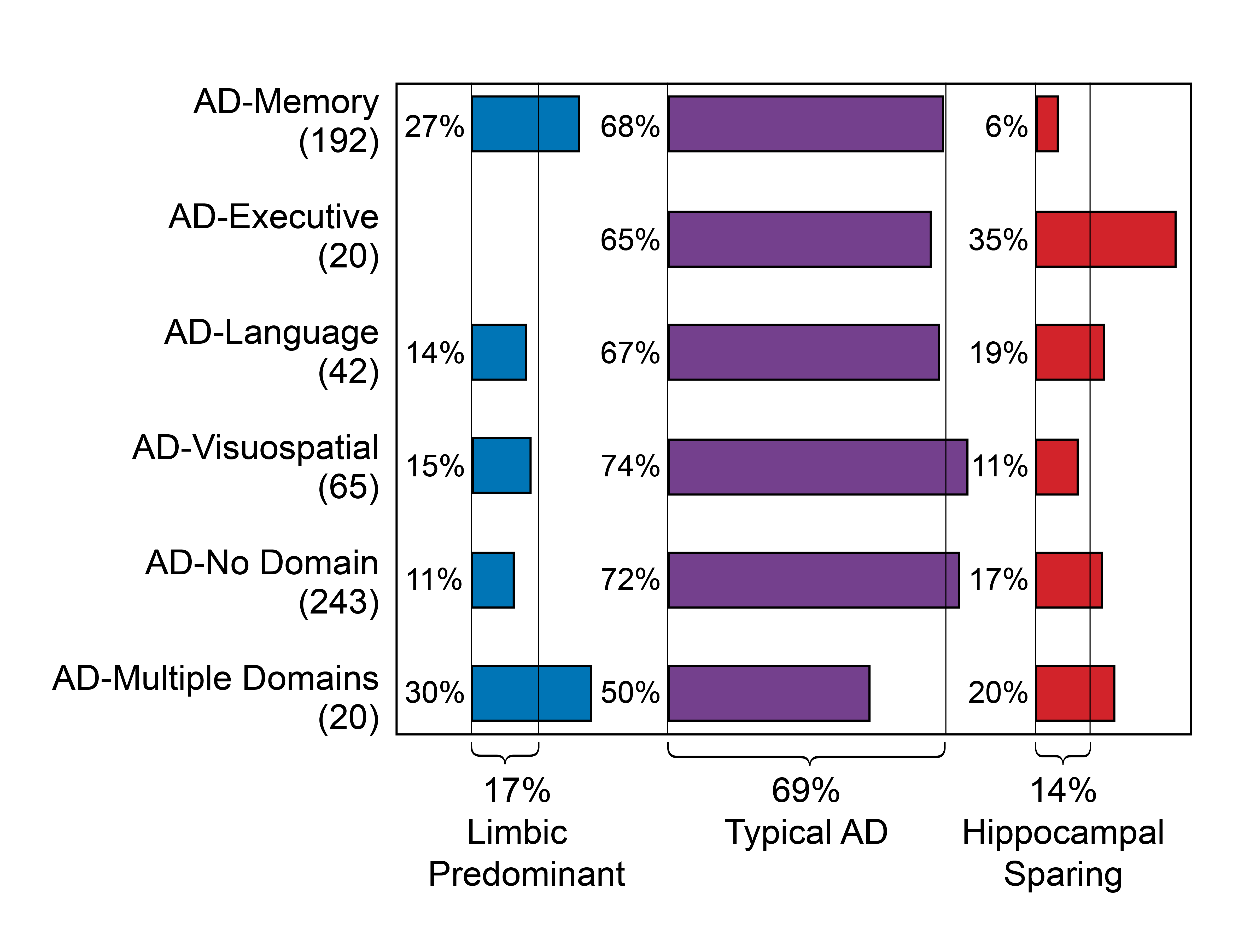 |
| --- |

* This Figure presents the same data as in Supplemental Table 2.

We used multinomial logistic regression to determine whether there was a difference in relative risk ratio for limbic predominant vs. typical AD and hippocampal sparing vs. typical AD. Those results are shown in Supplementary Table 5:

Supplementary Table 5: Multinomial logistic regression results of relative risk ratios associated with limbic predominant and hippocampal sparing vs. typical AD

| **Poulakis et al. subgroup** | **Typical Alzheimer’s Disease (Reference)** | **Limbic Predominant RRR (95% CI)** | **Limbic Predominant p value** | **Hippocampal Sparing RRR (95% CI)** | **Hippocampal Sparing p value** |
| --- | --- | --- | --- | --- | --- |
| AD-No Domain | Reference | Reference | Reference | Reference | Reference |
| AD-Memory | 1 | 2.53 (1.50, 4.25) | 4.6x10^-4^ | 0.35 (0.17, 0.71) | 0.0034 |
| AD-Visuospatial | 1 | 1.34 (0.61, 2.97) | 0.45 | 0.60 (0.26, 1.43) | 0.25 |
| AD-Language | 1 | 1.38 (0.52, 3.64) | 0.51 | 1.18 (0.50, 2.78) | 0.70 |
| AD-Executive | 1 | - | - | - | - |
| AD-Multiple domains | 1 | 3.87 (1.30, 11.50) | 0.02 | 1.66 (0.50, 5.54) | 0.41 |

Supplementary Fig. 27. Stability of atrophy-defined subgroups from study entry to Alzheimer’s dementia diagnosis for people with incident Alzheimer’s dementia (n=287)

| 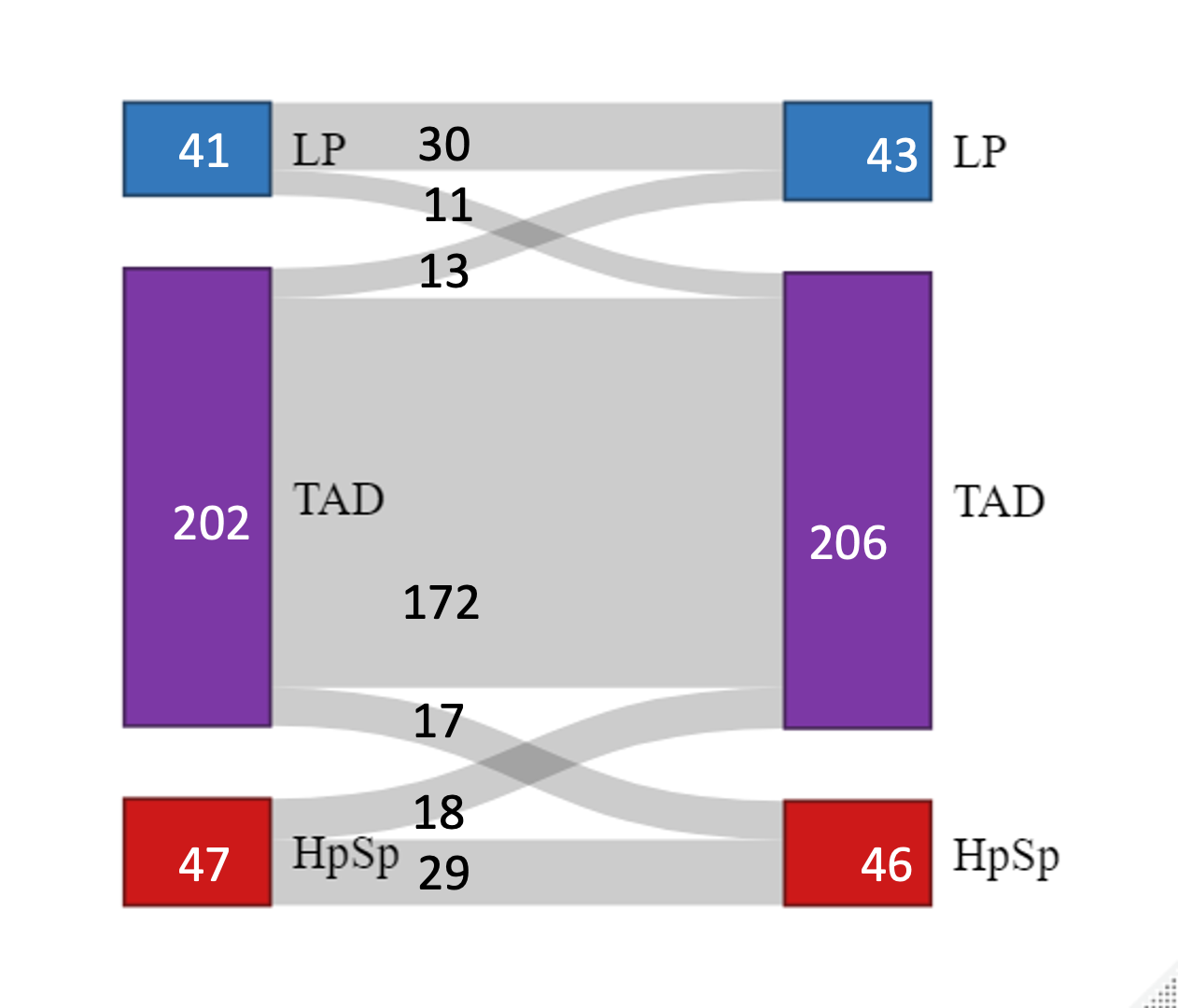 |
| --- |

Supplementary Fig. 28. Stability of atrophy-defined subgroups from Alzheimer’s dementia diagnosis to the most recent scan for people with prevalent or incident Alzheimer’s dementia (n=416)

| 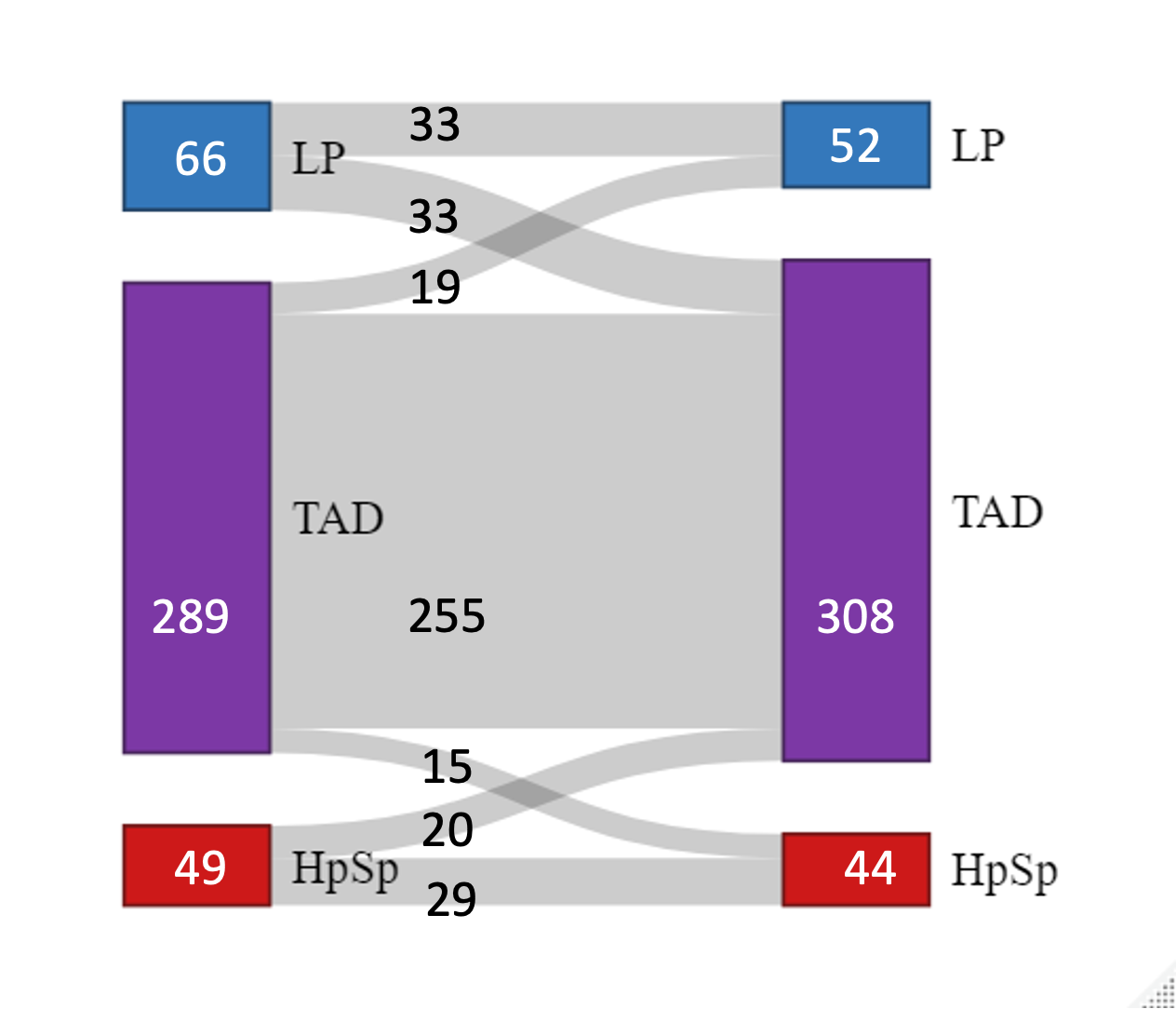 |
| --- |

Supplementary Table 6. Cognitively-defined subgroups and subgroups as defined by Poulakis et al. [2]

| **Poulakis et al. subgroup** | **AD-No Domain** | **AD-Memory** | **AD-Visuospatial** | **AD-Language** | **AD-Executive** | **AD-Multiple Domains** | **Totals (%)** |
| --- | --- | --- | --- | --- | --- | --- | --- |
| Hippocampal Sparing | 2 | 0 | 0 | 0 | 0 | 0 | 2 (1%) |
| Limbic Predominant | 37 | 25 | 9 | 6 | 3 | 5 | 85 (34%) |
| Limbic Predominant Plus | 7 | 3 | 1 | 2 | 0 | 0 | 13 (5%) |
| Minimal Atrophy | 56 | 51 | 19 | 6 | 4 | 8 | 144 (58%) |
| Diffuse Atrophy | 3 | 0 | 1 | 0 | 0 | 0 | 4 (2%) |
| Total (%) | 105 (42%) | 79 (32^) | 30 (12%) | 14 (6%) | 7 (3%) | 13 (5%) | 248 |

We compared these categorizations using a chi squared test; Chi squared with 20 degrees of freedom was 12.84 and a p value of 0.88. Given the large number of 0 cells we also used Fisher’s exact test, which gave a similar p value of 0.85. Two categories defined by Poulakis et al. dominated – limbic predominant and minimal atrophy. When we omitted the other groups we used multinomial logistic regression to determine whether there was a difference in relative risk ratio for minimal atrophy vs. limbic predominant. Those results are shown in Supplementary Table 3:

Supplementary Table 7: Multinomial logistic regression results of relative risk ratios associated with minimal atrophy vs. limbic predominant

| **Poulakis et al. subgroup** | **Limbic predominant (Reference)** | **Minimal atrophy RRR (95% CI)** | **Minimal atrophy p value** |
| --- | --- | --- | --- |
| AD-No Domain | Reference | Reference | Reference |
| AD-Memory | 1 | 1.43 (0.78, 2.63) | 0.25 |
| AD-Visuospatial | 1 | 1.49 (0.63, 3.53) | 0.36 |
| AD-Language | 1 | 0.59 (0.19, 1.82) | 0.36 |
| AD-Executive | 1 | 1.05 (0.22, 4.93) | 0.95 |
| AD-Multiple domains | 1 | 1.26 (0.38, 4.11) | 0.38 |

**Supplemental Text 1. More detailed discussion of asymmetry literature**

Sarica et al. [4] reviewed studies addressing asymmetry in people with Alzheimer’s disease compared with controls, including cortical thickness [5], cortical volumes [6-12], cortical surface area [13-15], as well as white matter properties [6, 16-20] and functional connectivity [21]. Most of these studies showed that structures on the left side had more atrophy than the right [6, 12-14, 16, 19, 22]. Other modalities that have been studied include amyloid deposition [23] and cerebral perfusion and metabolism [24-26].

References

[1] Risacher SL, Anderson WH, Charil A, Castelluccio PF, Shcherbinin S, Saykin AJ, et al. Alzheimer disease brain atrophy subtypes are associated with cognition and rate of decline. Neurology. 2017;89:2176-86.

[2] Poulakis K, Pereira JB, Muehlboeck JS, Wahlund LO, Smedby O, Volpe G, et al. Multi-cohort and longitudinal Bayesian clustering study of stage and subtype in Alzheimer's disease. Nat Commun. 2022;13:4566.

[3] Groot C, Grothe MJ, Mukherjee S, Jelistratova I, Jansen I, van Loenhoud AC, et al. Differential patterns of gray matter volumes and associated gene expression profiles in cognitively-defined Alzheimer's disease subgroups. Neuroimage Clin. 2021;30:102660.

[4] Sarica A, Vasta R, Novellino F, Vaccaro MG, Cerasa A, Quattrone A. MRI asymmetry index of hippocampal subfields increases through the continuum from the mild cognitive impairment to the Alzheimer's disease. Front Neurosci. 2018;12:576.

[5] Kim JH, Lee JW, Kim GH, Roh JH, Kim MJ, Seo SW, et al. Cortical asymmetries in normal, mild cognitive impairment, and Alzheimer's disease. Neurobiol Aging. 2012;33:1959-66.

[6] Muller MJ, Greverus D, Dellani PR, Weibrich C, Wille PR, Scheurich A, et al. Functional implications of hippocampal volume and diffusivity in mild cognitive impairment. Neuroimage. 2005;28:1033-42.

[7] Pennanen C, Testa C, Laakso MP, Hallikainen M, Helkala EL, Hanninen T, et al. A voxel based morphometry study on mild cognitive impairment. J Neurol Neurosurg Psychiatry. 2005;76:11-4.

[8] Shi F, Liu B, Zhou Y, Yu C, Jiang T. Hippocampal volume and asymmetry in mild cognitive impairment and Alzheimer's disease: Meta-analyses of MRI studies. Hippocampus. 2009;19:1055-64.

[9] Cherbuin N, Reglade-Meslin C, Kumar R, Sachdev P, Anstey KJ. Mild cognitive disorders are associated with different patterns of brain asymmetry than normal aging: the PATH through Life study. Front Psychiatry. 2010;1:11.

[10] Derflinger S, Sorg C, Gaser C, Myers N, Arsic M, Kurz A, et al. Grey-matter atrophy in Alzheimer's disease is asymmetric but not lateralized. J Alzheimers Dis. 2011;25:347-57.

[11] Dhikav V, Duraisamy S, Anand KS, Garga UC. Hippocampal volumes among older Indian adults: Comparison with Alzheimer's disease and mild cognitive impairment. Annals of Indian Academy of Neurology. 2016;19:195-200.

[12] Li B, Shi J, Gutman BA, Baxter LC, Thompson PM, Caselli RJ, et al. Influence of APOE genotype on hippocampal atrophy over time - an N=1925 surface-based ADNI study. PLoS One. 2016;11:e0152901.

[13] Thompson PM, Hayashi KM, de Zubicaray G, Janke AL, Rose SE, Semple J, et al. Dynamics of gray matter loss in Alzheimer's disease. J Neurosci. 2003;23:994-1005.

[14] Thompson PM, Hayashi KM, Dutton RA, Chiang MC, Leow AD, Sowell ER, et al. Tracking Alzheimer's disease. Ann N Y Acad Sci. 2007;1097:183-214.

[15] Long X, Zhang L, Liao W, Jiang C, Qiu B. Distinct laterality alterations distinguish mild cognitive impairment and Alzheimer's disease from healthy aging: statistical parametric mapping with high resolution MRI. Hum Brain Mapp. 2013;34:3400-10.

[16] Damoiseaux JS, Smith SM, Witter MP, Sanz-Arigita EJ, Barkhof F, Scheltens P, et al. White matter tract integrity in aging and Alzheimer's disease. Hum Brain Mapp. 2009;30:1051-9.

[17] Stricker NH, Schweinsburg BC, Delano-Wood L, Wierenga CE, Bangen KJ, Haaland KY, et al. Decreased white matter integrity in late-myelinating fiber pathways in Alzheimer's disease supports retrogenesis. Neuroimage. 2009;45:10-6.

[18] Liu Y, Spulber G, Lehtimaki KK, Kononen M, Hallikainen I, Grohn H, et al. Diffusion tensor imaging and tract-based spatial statistics in Alzheimer's disease and mild cognitive impairment. Neurobiol Aging. 2011;32:1558-71.

[19] Wessa M, King AV, Meyer P, Frolich L, Flor H, Poupon C, et al. Impaired and preserved aspects of feedback learning in aMCI: contributions of structural connectivity. Brain structure & function. 2016;221:2831-46.

[20] Yang C, Zhong S, Zhou X, Wei L, Wang L, Nie S. The abnormality of topological asymmetry between hemispheric brain white matter networks in Alzheimer's disease and mild cognitive impairment. Frontiers in aging neuroscience. 2017;9:261.

[21] Wang Z, Wang J, Zhang H, McHugh R, Sun X, Li K, et al. Interhemispheric functional and structural disconnection in Alzheimer's disease: a combined resting-state fMRI and DTI study. PLoS One. 2015;10:e0126310.

[22] Janke AL, de Zubicaray G, Rose SE, Griffin M, Chalk JB, Galloway GJ. 4D deformation modeling of cortical disease progression in Alzheimer's dementia. Magn Reson Med. 2001;46:661-6.

[23] Raji CA, Becker JT, Tsopelas ND, Price JC, Mathis CA, Saxton JA, et al. Characterizing regional correlation, laterality and symmetry of amyloid deposition in mild cognitive impairment and Alzheimer's disease with Pittsburgh Compound B. Journal of neuroscience methods. 2008;172:277-82.

[24] Friedland RP, Budinger TF, Koss E, Ober BA. Alzheimer's disease: anterior-posterior and lateral hemispheric alterations in cortical glucose utilization. Neurosci Lett. 1985;53:235-40.

[25] Loewenstein DA, Barker WW, Chang JY, Apicella A, Yoshii F, Kothari P, et al. Predominant left hemisphere metabolic dysfunction in dementia. Arch Neurol. 1989;46:146-52.

[26] Volkow ND, Zhu W, Felder CA, Mueller K, Welsh TF, Wang GJ, et al. Changes in brain functional homogeneity in subjects with Alzheimer's disease. Psychiatry research. 2002;114:39-50.
